# Celastrol Alleviates Autoimmune Hepatitis Through the PI3K/AKT Signaling Pathway Based on Network Pharmacology and Experiments

**DOI:** 10.3389/fphar.2022.816350

**Published:** 2022-03-10

**Authors:** Shuhui Wang, Zheng Huang, Yu Lei, Xu Han, Dean Tian, Jin Gong, Mei Liu

**Affiliations:** Department of Gastroenterology, Tongji Hospital of Tongji Medical College, Huazhong University of Science and Technology, Wuhan, China

**Keywords:** celastrol, network pharmacology, autoimmune hepatitis, immunological disorders, PI3K/akt signaling pathway

## Abstract

**Objective:** This work aims to explore the potential targets and underlying therapeutic mechanisms of celastrol in autoimmune hepatitis (AIH) through network pharmacology and experiments on Laboratory Animals.

**Methods:** A drug-target interaction network was constructed to predict the possible targets of celastrol and their potential relationship with the drug; docking studies were also performed for validation. This study used both acute and chronic rodent models of autoimmune hepatitis. Gross appearance of liver and spleen were obtained from murine models, hematoxylin-eosin staining and Sirius red staining were performed to examine hepatic inflammation and fibrosis respectively. By combining molecular docking and enrichment analysis results, the most prominent signaling pathway was selected and further confirmed by Western blot in AIH models administered with celastrol.

**Results:** In total, 82 common targets of celastrol and AIH were obtained from databases, identified by network pharmacology, and adequately enriched. Among them, PIK3R1, SRC, MAPK1, AKT1, and HRAS were selected as the top 5 closely related targets to celastrol. They all performed effectively in molecular docking, with AKT1 and PIK3R1 exhibiting more-prominent binding energy. Subsequently, celastrol administration significantly ameliorated hepatitis and liver fibrosis by reducing AKT1 and PI3K phosphorylation in both acute liver injury and chronic models of autoimmune hepatitis.

**Conclusion:** In summary, celastrol significantly attenuates autoimmune hepatitis by suppressing the PI3K/AKT signaling pathway, confirmed by validated animal models. These findings may help identify the mechanism involved in the anti-inflammatory action of celastrol in autoimmune hepatitis and provide ideas for future comprehensive studies.

## Introduction

Autoimmune hepatitis (AIH) is a chronic inflammatory disorder of the liver, characterized by elevation of serum immunoglobulin G (IgG), presence of autoantibodies and interface hepatitis on liver histology (Webb et al., 2018). It occurs across the globe affecting individuals of all ages, with a higher prevalence among females (Mieli-Vergani et al., 2018). Notably, the inflammatory context in the liver involves a complex cascade of molecular events; genetic and environmental risk factors significantly promote the pathogenesis of AIH (Floreani et al., 2018; Trivedi et al., 2019). Chronic liver inflammation may progress to liver cirrhosis, liver failure, and even hepatocellular carcinoma (Wu J. et al., 2019). Although the use of corticosteroids with or without azathioprine is introduced as the first-line treatment, some patients obtain little benefit from standard of care, hence causing resistance or relapse. Therefore, identifying novel therapeutic approaches for patients not benefiting from first-line treatments is necessary (Taubert et al., 2018; van den Brand et al., 2019; Engel et al., 2020).

The concanavalin A (ConA) model, mediated by T cells, is a well-accepted rodent model for studying acute immune-mediated liver injury with partial features resembling autoimmune hepatitis (Schippers et al., 2021). Since it can easily be recognized by liver-kidney microsomal antibodies 1 (LKM-1), cytochrome P4502D6 (CYP2D6) is a major autoantigen in type 2 AIH (Heneghan et al., 2013; Webb et al., 2018). Liver-specific overexpression of human gene coding CYP2D6 in mice can induce chronic progress of autoimmune liver damage, in turn leading to autoantibody generation (Liu S.-P. et al., 2020; Wang H. et al., 2020). Therefore, the present study examined the biological effects of celastrol in both acute and chronic experimental models of AIH.

Traditional Chinese medicines (TCM) comprise a treasure house for novel drug research. Previous literature has indicated that celastrol, isolated from the root bark of Tripterygium wilfordii Hook. f., has important therapeutic significance (Deng et al., 2020). Because of its anti-tumor, anti-oxidant, and anti-angiogenic properties, celastrol acts against myeloma, ovarian cancer, myeloid leukemia, pancreatic cancer and thyroid carcinoma. Meanwhile, it has anti-inflammatory effects and is effective against diet-induced obesity, inflammatory bowel disease, and other autoimmune diseases, including systemic lupus erythematosus (Pinna et al., 2004; Tozawa et al., 2011; Abdin and Hasby 2014; Ju et al., 2015; Venkatesha et al., 2016; Yang et al., 2017; Song X. et al., 2019; Venkatesha and Moudgil, 2019; An et al., 2020; An et al., 2020; Liu Y. et al., 2020; Chen et al., 2020; Lagoa et al., 2020; Lu et al., 2020). Nonetheless, the understanding of its efficacy in AIH is limited.

Herbal medicines harbor various bioactive ingredients acting against multiple targets rather than a single target (Yuan et al., 2020). In 2008, the concept of “network pharmacology” was proposed by Hopkins and has so far evolved as an emerging field of pharmacology. The central idea behind network pharmacology is to understand the multi-targeted agents; thus, network pharmacology methods may help identify complex mechanisms of TCM (Lee et al., 2019; Tang et al., 2020).

## Materials and Methods

### Data Preparation

A two-dimensional (2D) structure of celastrol was obtained from the PubChem database. The online resources in systems pharmacology (http://lsp.nwu.edu.cn/index.php) and PharmMapper (http://lilab-ecust.cn/pharmmapper/) were used to predict the potential targets of celastrol. After integrating the data and removing duplicates, all proteins were confirmed using the UniProt database (https://www.uniprot.org/). Genes related to the term “autoimmune hepatitis” were extracted from the GeneCards database (https://www.genecards.org/).

The intersection between potential targets and associated genes was obtained and presented by a Venn diagram.

### Protein-Protein Interaction Target Network

The Search Tool of Retrieval of Interacting Genes database (http://string-db.org/)was used to construct a PPI systematic network and visualize the result. The shared proteins with a combined score of >0.9 were selected for PPI analysis, and disconnected nodes were hidden in the network. Network visualization and analysis were conducted using the Cytoscape software 3.6.0 (Shannon et al., 2003). The node size and color were used to present the degree (number of the edges), analyzed by network analyzer of Cytoscape.

### Enrichment Analysis

Gene enrichment analysis of the screened drug targets and intersectional targets was performed to further elucidate the mechanisms underlying the potency of celastrol. Both KEGG signaling pathway enrichment and GO biological functional enrichment analyses were performed using Metascape (http://metascape.org/gp/index.html#/main/step1) (Zhou et al., 2019). All genes in the genome were used as the enrichment background. Terms with a *p*-value of < 0.01, a minimum count of 3, and an enrichment factor of > 1.5 were collected and grouped into clusters based on their membership similarities. Data visualization was performed using an online platform (http://www.bioinformatics.com.cn).

### Molecular Docking Analysis

The PDB database (http://www.rcsb.org/) was used to obtain the crystal structures of candidate targets of celastrol. Docking studies were conducted between celastrol and key targets using Autodock 4.2. The structure modification process included ligand and water removal, hydrogen addition, amino acid optimization, and patching (Pradeepkiran et al., 2015; Yin et al., 2020; Li et al., 2021; Venkataramaiah et al., 2021).

### Experimental Autoimmune Hepatitis Models

Male C57BL/6 mice (6–8 weeks old, 20–25 g) were supplied by the Laboratory Animal Centre of Tongji Hospital. They were housed in the SPF environment with an alternating 12 h light/dark cycle and had free access to food and water. Concanavalin-A (ConA) was purchased from Sigma-Aldrich; Merck KgaA, celastrol was obtained from MCE (HY-13067). Mice were randomly divided into normal control group (*n* = 6), celastrol (Cel) group (*n* = 6), ConA group (*n* = 7) and ConA + celastrol (ConA + Cel) group (*n* = 7). According to the existing literature and the results of our pre-experiments, the mice in the celastrol group and the ConA + Cel group were perfused with celastrol once daily in doses of 3 mg/kg for a week (Yang et al., 2006; Cascao et al., 2012; Guan et al., 2016; Wang Y. et al., 2020). After 5 days of daily gavage, the mice in the ConA + Cel group were injected with ConA via tail vein at a dose of 10 mg/kg. The ConA groups were injected via tail vein with the same dose of ConA simultaneously. Pre-experiments of different celastrol concentration are shown in supplementary materials (Supplementary Figure S1).

Plasmid pCYP2D6 was obtained from our lab preserved. The remaining mice were randomly divided into control-c group (*n* = 6), CYP2D6 (CYP) group (*n* = 8), CYP2D6+celastrol (CYP + Cel) group (*n* = 8), celastrol-chronic (Cel-c) group (*n* = 6). For mice in the CYP and CYP + Cel groups, multiple high-pressure tail vein injections of plasmid were used to develop AIH mouse models as reported in previous literature (Wang et al., 2019; Liu S.-P. et al., 2020; Wang H. et al., 2020). Twenty-one days after the adenovirus injection, the CYP + Cel and Cel group mice were orally administered with celastrol (3 mg/kg) once a day for 14 days.

**FIGURE 1:**
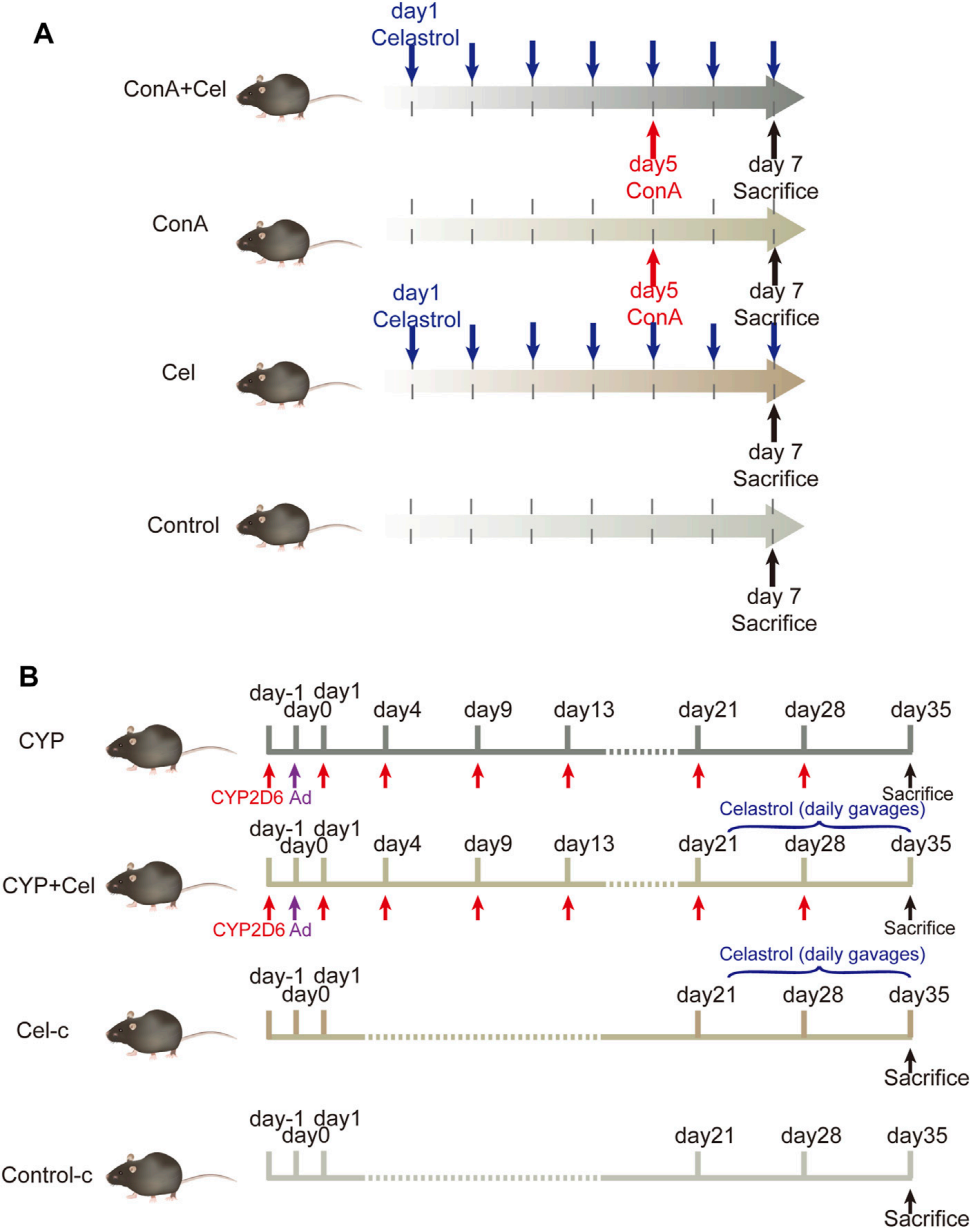
Animal models and experimental design. **(A)** The intervention cycle in ConA-induced acute liver injury models. **(B)** The intervention cycle in chronic AIH models.

The animal models and treatment scheme was established as previously described (Figure 1). For the ConA mice model, all mice were sacrificed 48 h after injecting ConA for further study. Additionally, the mice in AIH models were sacrificed on the 35th day, and relative specimens were collected. The animal studies were approved by the Institutional Animal Care and Use Committee of Tongji Hospital.

### Histopathology and Biochemical Analysis

The isolated fresh liver tissues were fixed in 4% paraformaldehyde for 48 h and embedded in paraffin. Hematoxylin-eosin (HE) staining was analyzed to evaluate the liver injury. Meanwhile, Sirius-red staining was performed to evaluate the degree of fibrosis in chronic models.

Blood samples were collected by extracting the eyeball after anesthesia. The whole blood was collected in tubes, and the serum was isolated through centrifugation at 3,500 rpm for 10 min. Subsequently, serum levels of AST and ALT were measured by the clinical laboratory of Tongji Hospital (Wuhan, China).

### Western Blotting

Liver tissue samples were stored at −80°C, then digested in RIPA buffer containing phosphatase inhibitor, cocktail, and PMSF (Wuhan Sevier Biotechnology Co., Ltd.). All samples were centrifuged at 12,000 x g for 10 min after grinding and sonication. Protein concentration was measured via the a BCA assay. Then, 60 μg protein was loaded on 10% Bis-Tris gel and separated by SDS-PAGE. The membranes were blocked with TBST containing 5% BSA (Absin, Shanghai, China) at room temprature for 1.5 h. The primary antibodies applied overnight at 4°C included: anti-AKT1 (1:1,000, CST, #2938); anti-p-AKT1 (1:1,000, CST, #9018); anti-PI3K (1:1,000, CST, #4257); anti-p-PI3K (1:1,000, CST, #4228); anti-GAPDH (1:20,000, Proteintech, 60004-1-Ig). On the next day, secondary antibodies were incubated at room temperature for 1.5 h. All membranes were washed 3 times using TBST for 10 min, then exposed to hypersensitive electrochemiluminescence (ECL) reagents (NCM Biotech, Suzhou, China). WB images were analyzed using the Image J software.

### Statistical Analysis

The resulting data were analyzed using GraphPad Prism 8.0 software. The results were presented as mean ± standard deviation (SD). The Student’s *t*-test was used to analyze the comparison between two groups. *p* < 0.05 was considered statistically significant.

## Results

### Collection of the Targets Involving Celastrol and Autoimmune Hepatitis

**FIGURE 2:**
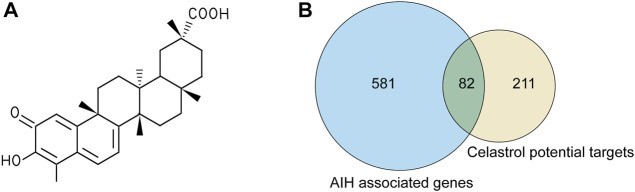
The targets of celastrol were collected to explore the drug–disease relationships. **(A)** 2D structure of celastrol. **(B)** Venny diagram of celastrol and AIH intersection targets. Note: 82 celastrol-AIH intersection targets are shown in the middle.

Celastrol is an active ingredient isolated from Tripterygium wilfordii Hook. f.; its 2D structure is shown in Figure 2A. In total, we screened 292 targets of celastrol obtained from the above-mentioned databases. The results in GeneCards showed that 5,298 genes are potentially related to AIH; 663 genes were included in the present study, as the median relevance score was used as the cutoff. The Venn diagram showed 82 candidate genes screened through the intersection of the two groups of genes (Figure 2B).

### Topological Network Analysis of the Collective Targets

**FIGURE 3:**
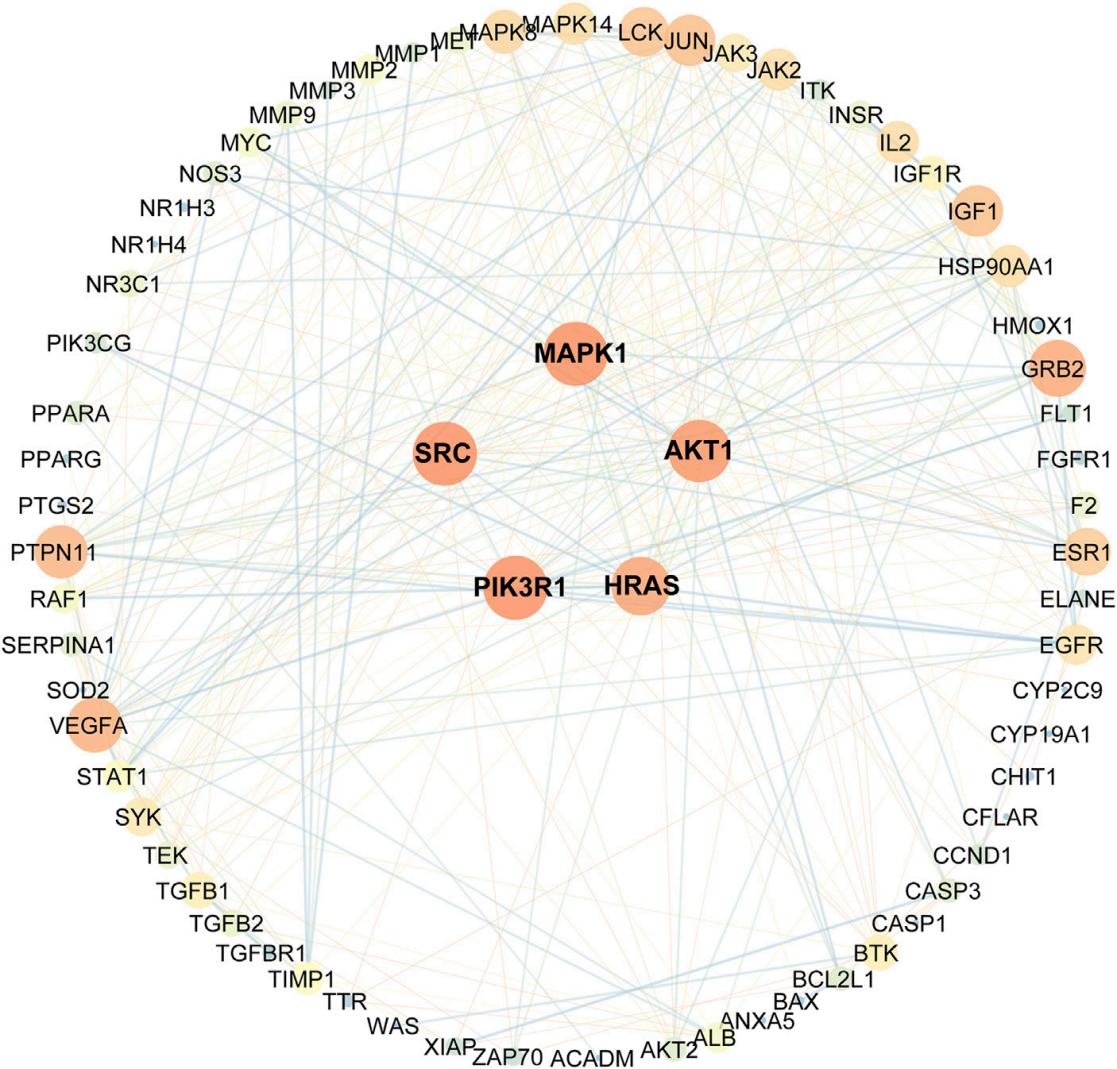
Protein-protein interactions network schematic of intersection targets of celastrol and AIH. The warm color tone and node size indicate higher degree, the thick red lines indicate closer structure linkage.

As indicated in the STRING database, 82 collected genes were used to construct the protein-protein interaction network comprising 329 edges. The average node degree was 8.02, whereas the average local clustering coefficient was 0.5 (Figure 3). In this network, nodes represented the obtained common targets; the higher the degree value, the larger the node; the edges between nodes indicated interaction relationships.

Eventually, based on the degree and above-average betweenness centrality, we defined 5 key players in the activity of celastrol against AIH: PIK3R1, SRC, MAPK1, AKT1, HRAS (Table 1).

**TABLE 1:** Top 5 targets with potentially critical roles in celastrol treatment of autoimmune hepatitis.

NO	Gene Abbreviation	Degree	Closeness Centrality	Betweenness Centrality
1	PIK3R1	27	0.5528	0.0524
2	MAPK1	27	0.5862	0.1236
3	SRC	27	0.5862	0.0886
4	AKT1	26	0.5862	0.1389
5	HRAS	24	0.5397	0.0343

### Systemic Exploration of Target Proteins Through Enrichment Analyses

**FIGURE 4:**
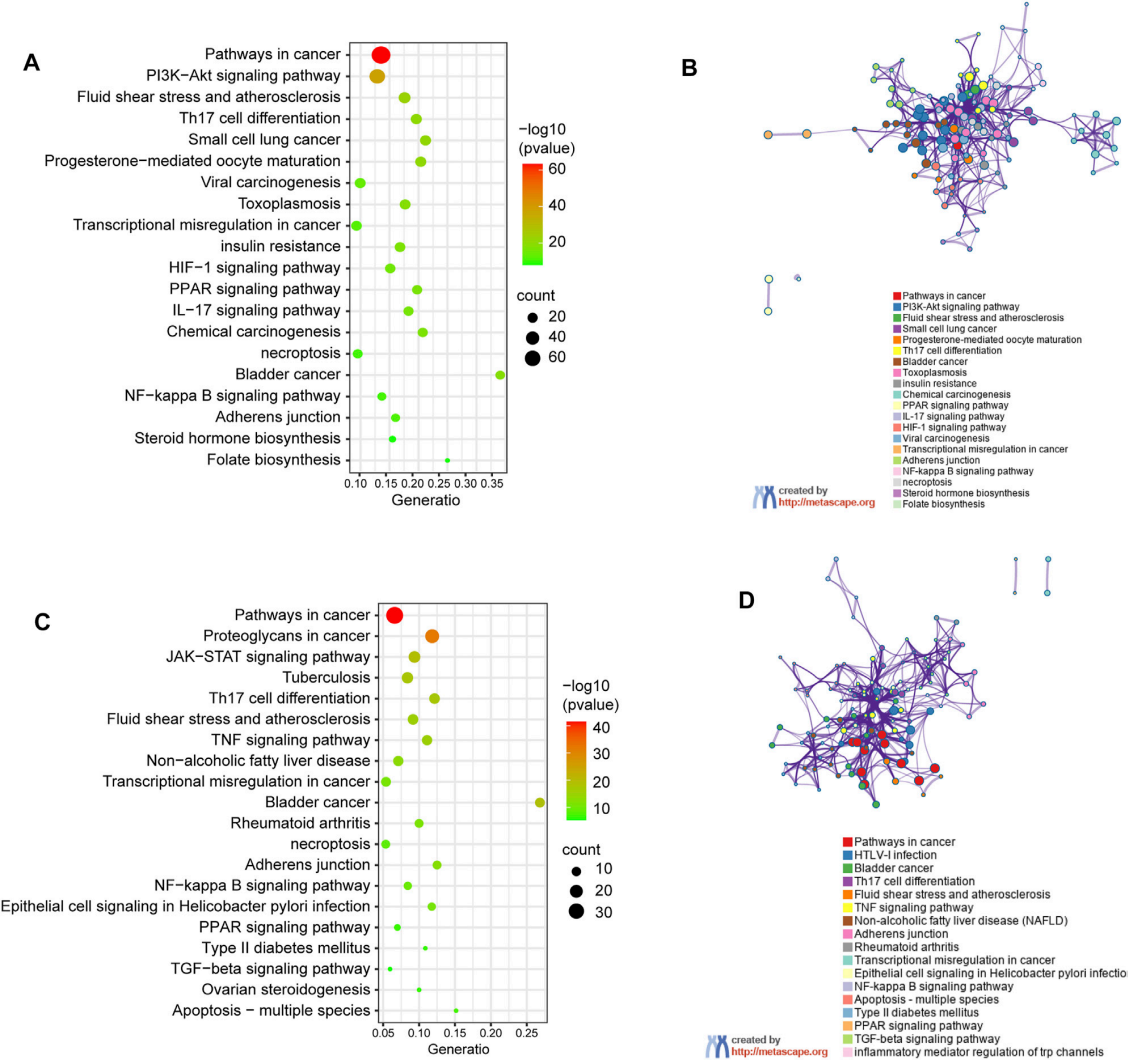
KEGG pathway enrichment analysis of genes encoding proteins targeted by celastrol. **(A)** Signalling pathways involved in predicted targets of celastrol. **(B)** Network of enriched terms in **(A)**: colored by cluster ID, where nodes that share the same cluster ID are typically close to each other. **(C)** Signalling pathways involved in intersection targets of celastrol and AIH. **(D)** Network of enriched terms in **(C)**.

Genes encoding proteins targeted by celastrol are involved in signaling pathways (Figure 4A) including pathways in cancer (hsa05200), PI3K/Akt signaling pathway (hsa04151), fluid shear stress and atherosclerosis (ko05418), Th17 cell differentiation (ko04659), as well as small cell lung cancer (hsa05222). To further understand the relationships between the terms, a subset of enriched terms were selected and rendered as a network plot, where each node represented an enriched term and colored first by its cluster ID (Figure 4B).

The pathway enrichment analysis showed that the shared genes encoding proteins, also treated as the potential drug targets are associated with pathway in cnacer (hsa05200), proteoglycans in cancer (ko05205), JAK-STAT signaling pathway (hsa04630), bladder in cancer (ko05219), and *tuberculosis* (ko05152). The top 20 clusters are shown in Figure 4C. The network of enrichment terms was illustrated as previously described (Figure 4D).

**FIGURE 5:**
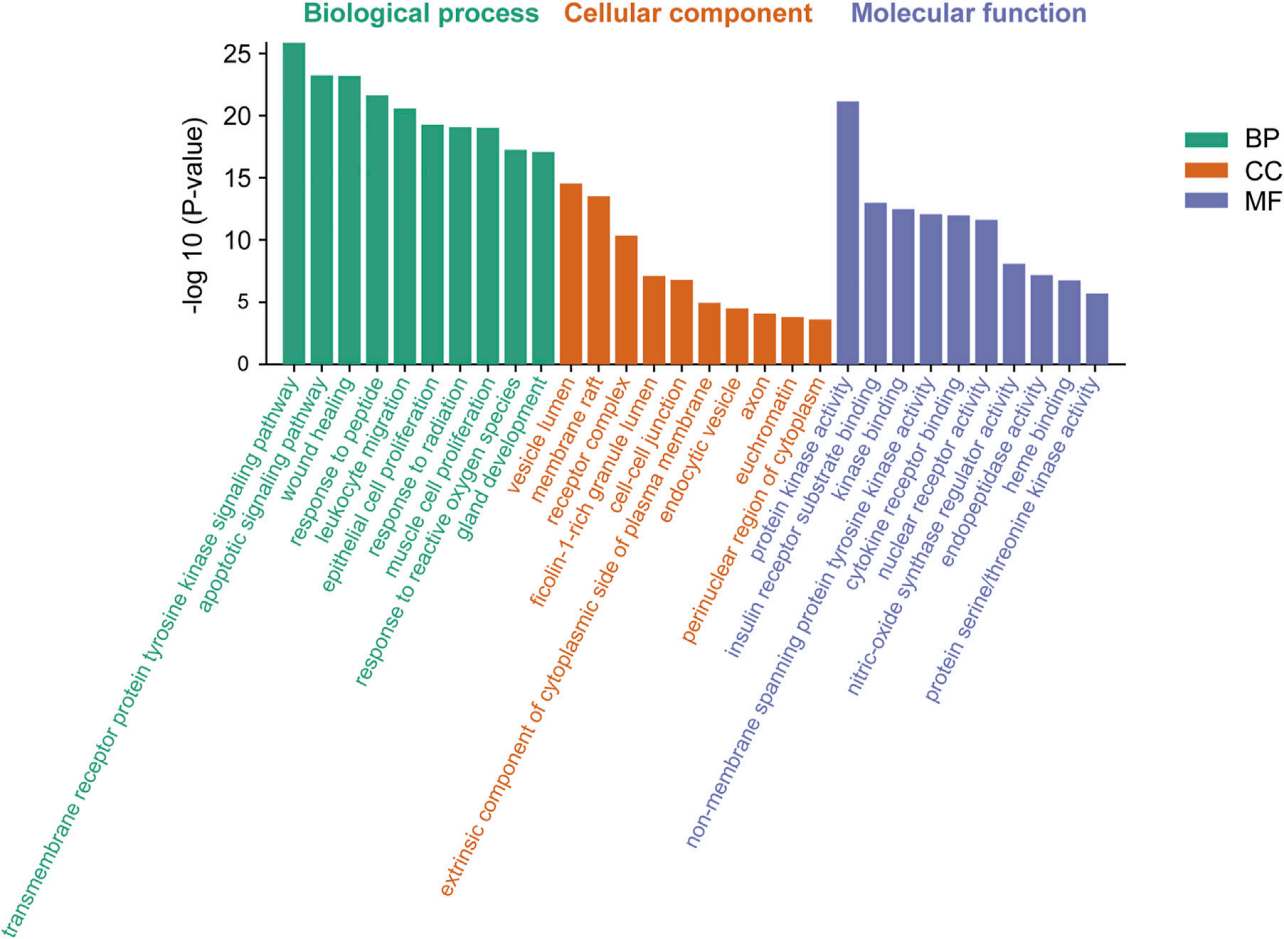
Gene Ontology (GO) enrichment analysis of genes encoding the intersection targets. The terms in green represent biological processes, the terms in orange represent cellular components, and the terms in purple represent molecular functions.

Meanwhile, based on their significance level (*p* < 0.01), the top 10 significantly enriched terms were selected in BP, MF, and CC categories, respectively (Figure 5). GO enrichment analysis revealed that the collective genes encoding proteins are involved in various biological processes (BP), primarily reflected in the transmembrane receptor protein tyrosine kinase signaling pathway (GO:0007169), apoptotic signaling pathway (GO:0097190), and wound healing (GO:0042060). Molecular functions (MF) of these proteins majorly contain protein kinase activity (GO:0004672), insulin receptor substrate binding (GO:0043560), and kinase binding (GO:0019900). Meanwhile, clusters of cellular components (CC) were also enriched. According to the *p*-value, vesicle lumen (GO:0031983), membrane raft (GO:0045121), and receptor complex (GO:0043235) were considered to be the top 3 clusters.

### Molecular Docking

**FIGURE 6:**
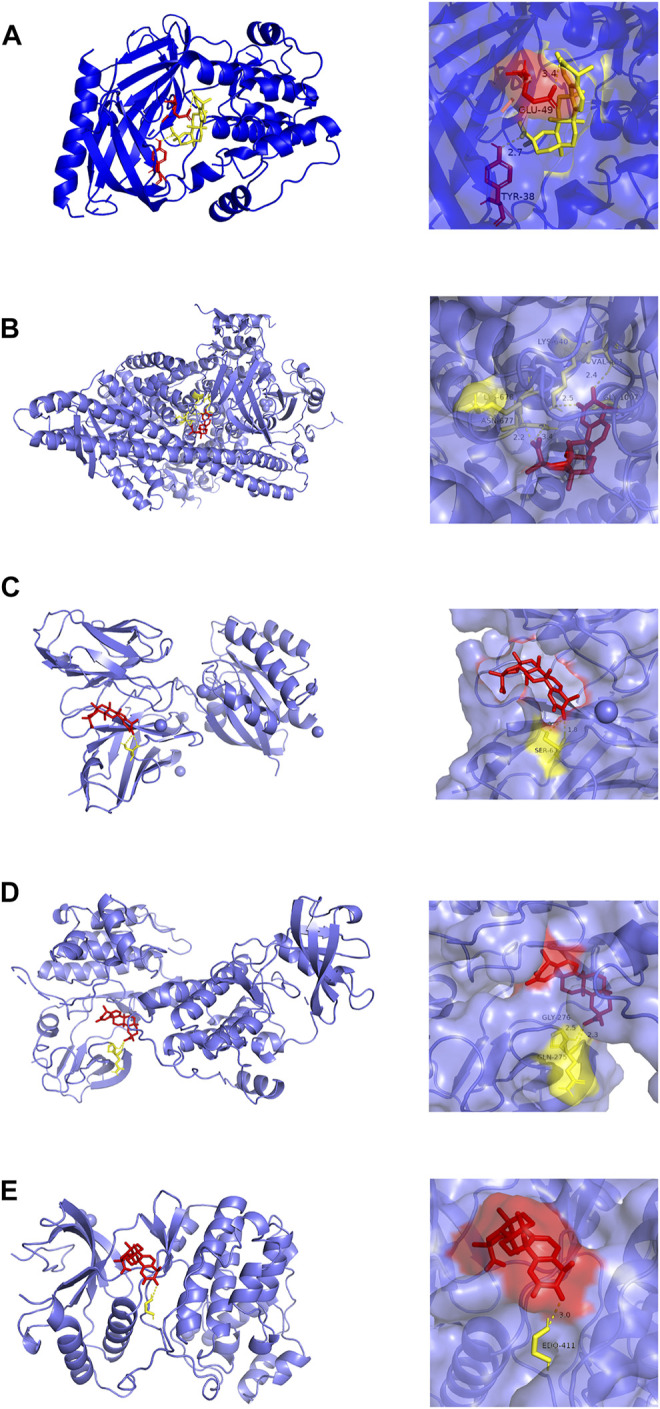
Molecular models of celastrol binding to the 5 most probable binding targets. Proteins **(A)** AKT1, **(B)** PIK3R1, **(C)** HRAS, **(D)** SRC, **(E)** MAPK1 are shown interacting with a celastrol molecule. The binding amino acidic sites and other details are shown on the right.

Molecular docking analysis visually showed the interaction between celastrol and its potential protein targets associated with AIH (Figure 6). Schematic diagrams of drug-target binding mode are displayed on the left, and the details are on the right. Based on online analysis (Autodock 4.0), the binding energy of celastrol with each target was low. Among these, AKT1 and PIK3R1 exhibited significantly lower binding energy (Table 2).

**TABLE 2:** Different binding energies of celastrol polysulfides with 5 selected targets.

Rank	Target	Affinity (kcal/mol)
1	AKT1	−13.1
2	PIK3R1	−11.3
3	SRC	−8.5
4	HRAS	−8.2
5	MAPK1	−7.6

### Celastrol Alleviates Inflammation in Mice With Autoimmune Hepatitis

**FIGURE 7:**
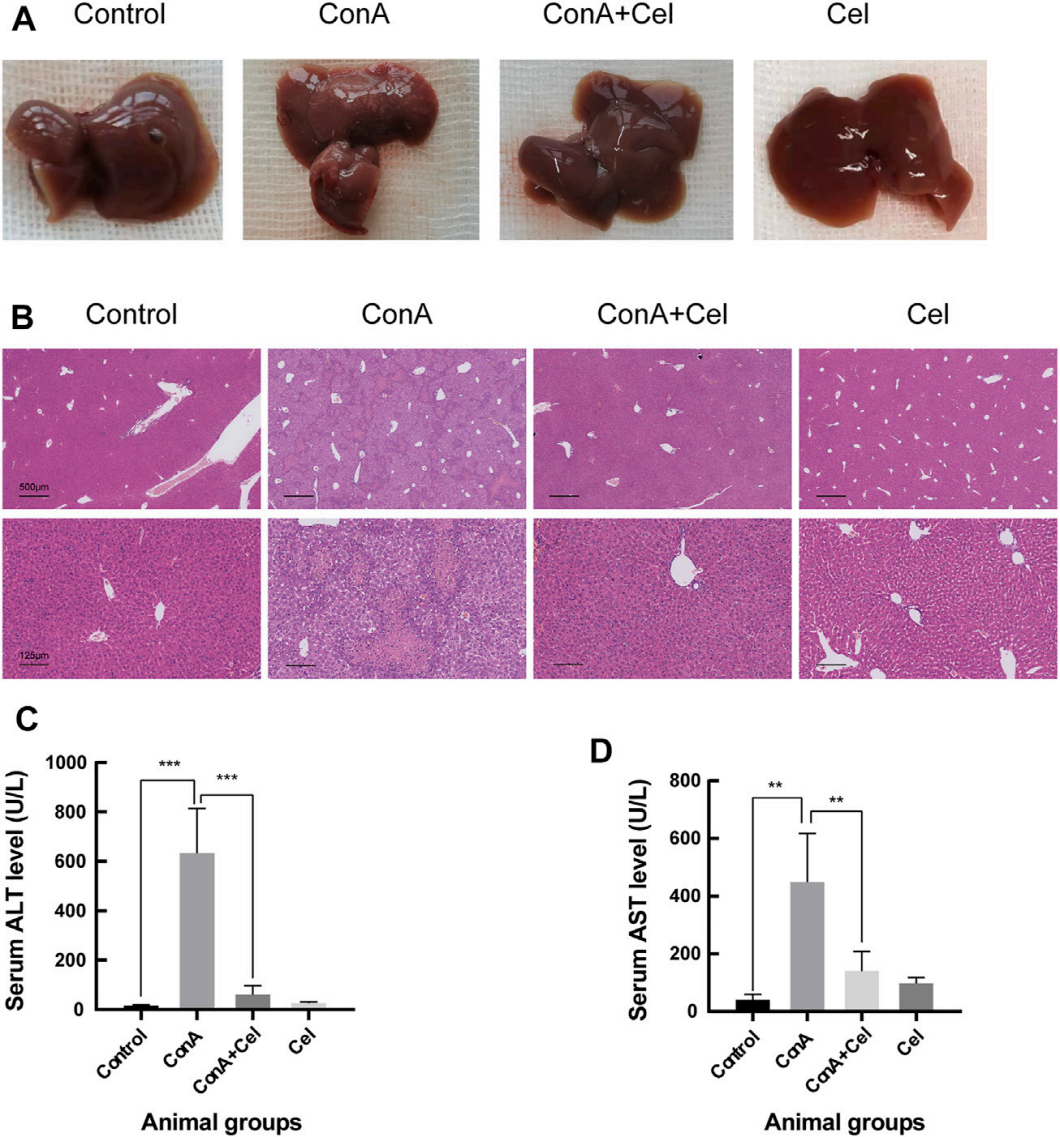
ConA-induced liver damage was ameliorated in celastrol treated mice. **(A)** Gross appearance of livers. **(B)** Representative photomicrographs of HE stained liver sections. **(C)** Quantification of serum ALT (**p < 0.*01*, ***p < 0.*001*). **(D)** Quantification of serum AST.

For the ConA induced acute injury models (Figure 7A), gross appearance demonstrated potent anti-inflammatory effects of celastrol. HE staining revealed significant inflammatory cells infiltration and hepatic necrosis in ConA group mice (Figure 7B). However, liver cells of the control and treatment groups were closely and ordrly arranged, with a normal structure of hepatic lobules. As similar to the trend of histological results, levels of alanine aminotransferase and aspartate aminotransferase in plasma samples were significantly increased in ConA mice (Figures 7C,D).

**FIGURE 8:**
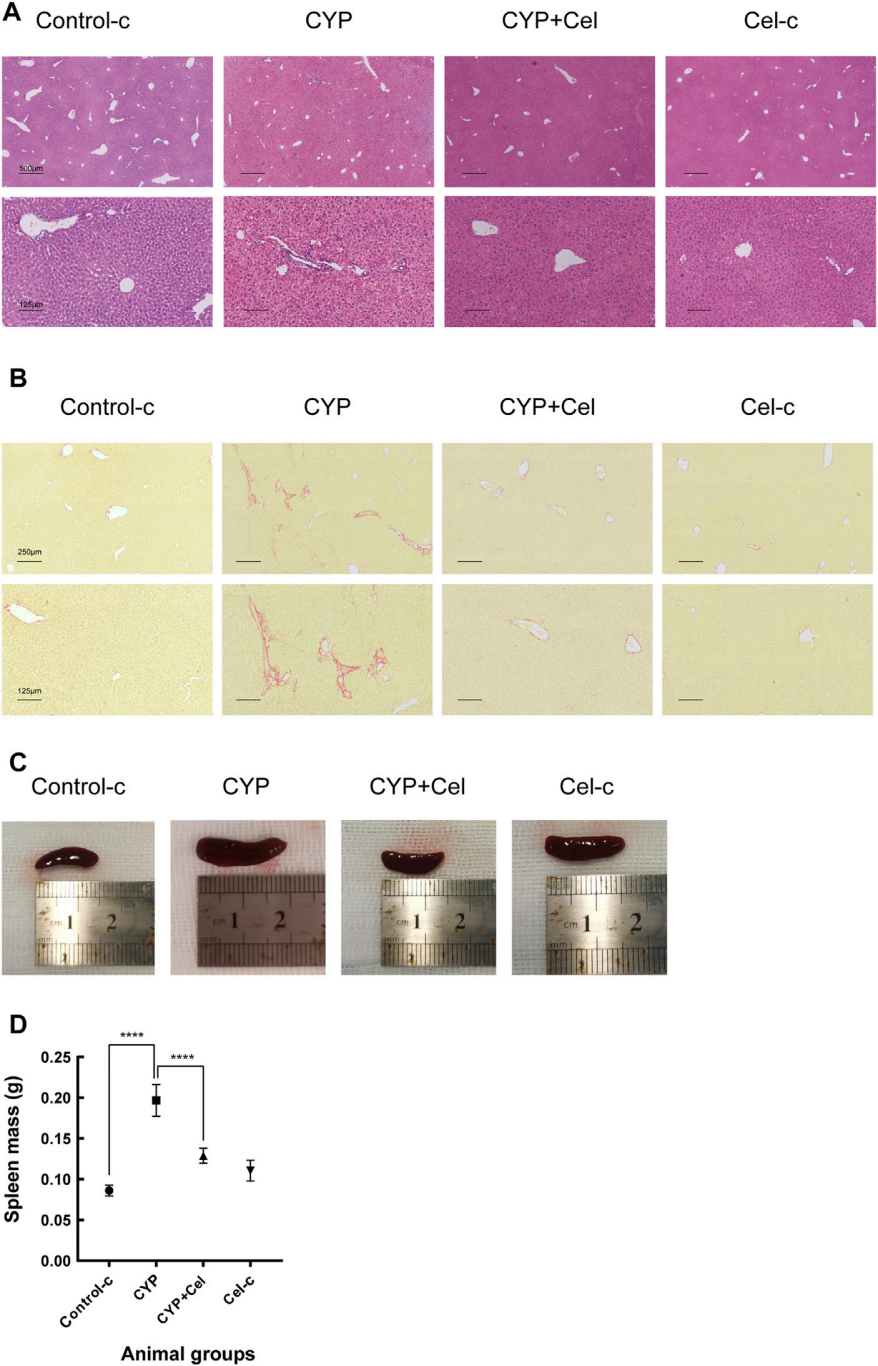
Inflammatory infiltration and liver injury of experimental AIH models were alleviated in celastrol treated mice. Representative photographs of HE stained **(A)** and Sirius Red stained **(B)** liver sections. **(C,D)** Differential sizes of the spleens from each group of animals, ****p < 0.*0001*.

For the AIH mice models, inflammatory cell infiltration was shown in the portal area, with the formation of interface hepatitis (Figure 8A). Sirius-Red staining revealed evidence of more collagen fiber deposition in the CYP group than that in the CYP + Cel group. This indicates that celastrol improved liver fibrosis in autoimmune hepatitis mice (Figure 8B). Also, chronic inflammation resulted in an enlarged spleen, whereas celastrol alleviated this phenotype (Figures 8C,D).

### Celastrol Inhibits the Activation of the PI3K/AKT Pathway in Autoimmune Hepatitis

**FIGURE 9:**
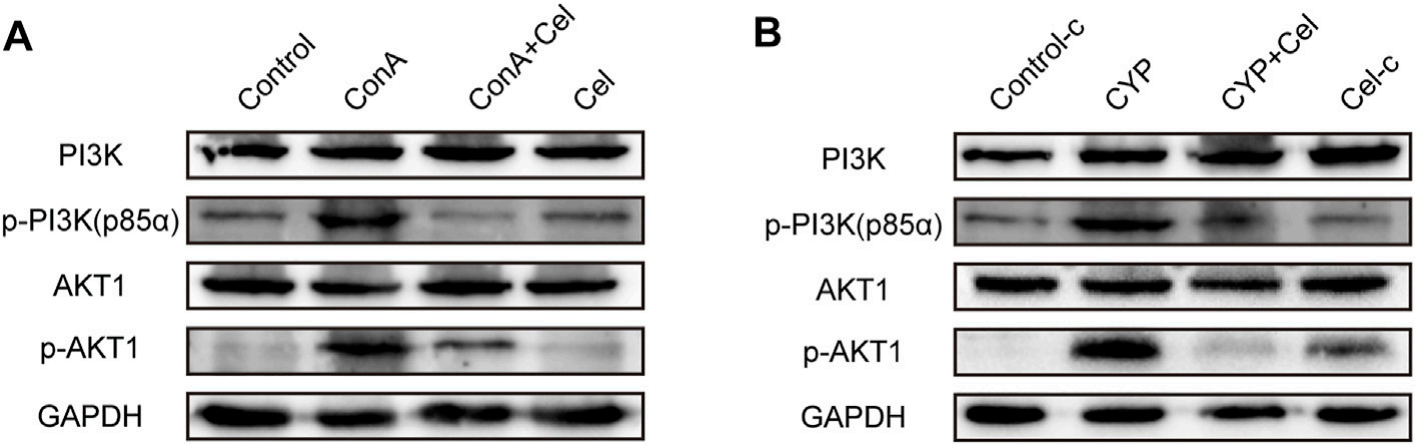
Effect of celastrol on phosphorylation of PI3K/AKT pathway proteins. **(A)** and **(B)** represent the inhibition effect of celastrol in ConA-induced liver injury and experimental AIH models, respectively. Supplementary Figure S1. Pre-experiment of therapeutic effect of different drug concentration. **(A)** Gross appearance of livers. **(B)** Body weight curve.

As suggested by the results of KEGG and molecular docking, the effect of celastrol was assessed on the PI3K/AKT pathway activation or suppression. As shown in Figure 9, Akt1 and the p85 subunit of PI3K were phosphorylated in both ConA induced and experimental AIH models; however, celastrol treatment inhibited this activation effect.

## Discussion

Autoimmune hepatitis (AIH) is a progressive autoimmune-mediated liver inflammatory disorder with a poorly understood etiology and no specific therapeutics (Zhang et al., 2018). Its underlying etiologies are increasingly diverse, including environmental and genetic factors (Saito et al., 2020; Tanaka et al., 2020; Vollmer et al., 2020). A search for novel therapeutic strategies is necessary to manage the socio-economic burden of AIH. Celastrol is widely known as an effective proteasome inhibitor, whose inhibition of proteasomal activity potentially induces apoptosis in cancer cells (Kannaiyan et al., 2011; Hou et al., 2020; Shi et al., 2020). Previous studies have also indicated that celastrol inhibits interferon response through targeting IRF3 activation and may be an effective treatment for interferon response-dependent autoimmune diseases (Liu Y. et al., 2020). Here, we established two typical experimental autoimmune hepatitis models (Wang et al., 2019; Liu S.-P. et al., 2020; Wang H. et al., 2020), and found that celastrol alleviates inflammatory responses in both animal models.

Information from public databases was integrated to explore interactions between celastrol and its potential protein targets in AIH, as well as relevant signaling pathways in which celastrol targets participate. As a consequence, 82 collective targets of celastrol and AIH were identified, among which PIK3R1, SRC, MAPK1, AKT1, and HRAS exhibited a closer association with autoimmune hepatitis. Also, docking studies were performed to predict specific interactions between celastrol and its predicted targets; notably, AKT1 and PIK3R1 bound well with celastrol. AKT1 is the central member of the PI3K/AKT signaling pathway, and PIK3R1 codes for phosphatidylinositol 3-kinase (PI3K) regulatory subunit alpha (p85-ALPHA) (Marquer et al., 2014; Zhang J. et al., 2021). As suggested by molecular docking and KEGG analysis, the PI3K/AKT signaling pathway suppression may lead to the treatment effect of celastrol in AIH; this was confirmed by Western blotting.

The PI3K/AKT signaling pathway is closely related to numerous inflammatory-associated diseases and is considered a key target for tumors (Kannaiyan et al., 2011; Song M. et al., 2019; Zhu et al., 2020). It is worth mentioning that, celastrol plays a role in inhibiting PI3K/AKT signaling pathway in the treatment of various diseases, such as glioblastoma and prostate cancer (Pang et al., 2010; Kannaiyan et al., 2011). Besides, it is thought to be correlated with the cell cycle (Zhang M. et al., 2021). Li H et al. reported that PTEN, a negative regulating factor, may suppress the PI3K/AKT pathway and maintain moderate T cell proliferation in healthy individuals (Li et al., 2019). Herrero-Sánchez M^a^ Carmen et al. revealed that the PI3K/AKT pathway is closely related to the proliferation of T cells in graft-versus-host disease; meanwhile, the ability of pathway inhibitors (BKM120 and BEZ235) to suppress activated T cell proliferation were confirmed (Herrero-Sanchez et al., 2016). Altered T cell regulation is as an important factor that changes the risk of developing autoimmune hepatitis. The mononuclear infiltrate in periportal areas predominantly comprises CD4+T lymphocytes (De Biasio et al., 2006; Webb et al., 2018). Impairment of T cell regulation is mainly reflected in the imbalance between liver-antigen-specific regulatory T cells and effector T cells, especially the abnormal ratio of Treg/Th17 cells (Heneghan et al., 2013). Nevertheless, whether the numerical change or functional defect in regulatory T cells causes a collapse of the immune environment in the liver remains unclarified (Longhi et al., 2004; Longhi et al., 2005). Our findings suggest that celastrol exerts therapeutic effects against AIH, at least partly by regulating the activation of the PI3K/AKT signaling pathway. Whether modulating T cell proliferation is a crucial pathway, the defined mechanism is still poorly understood and awaits further study, including flow cytometric analysis, etc. Similarly, whether celastrol restores the T cell-related immunity balance will be an interesting focus for further studies.

Meanwhile, the antifibrotic efficacy of celastrol in chronic AIH models was also reflected in this study. Celastrol has previously been reported to have anti-fibrotic effect by activating AMPK-SIRT3 signaling in activated stellate cell (HSCs) induced by CCL_4_ (Wang Y. et al., 2020). The PI3K/AKT signaling pathway, involed in this study, has also been reported to be related with liver fibrosis and HSCs proliferation (Lou et al., 2017; Wu W. et al., 2019). Previously studies found that overexpression or recombinant treatment of SPOCK1 promoted HSCs activation and proliferation by activating the PI3K/Akt signaling pathway (Du et al., 2020). Therefore, inhibition of PI3K/AKT signaling pathway may inhibit the activation of HSCs and liver fibrosis. However, whether celastrol directly inhibited HSCs activation through PI3K/AKT signaling pathway in AIH treatment awaits further investigation.

In conclusion, we developed comprehensive network pharmacology to identify targets of celastrol in AIH and preliminary confirmed the suppressed PI3K/AKT pathway-mediated treatment efficacy in two typical rodent models. This study provides a basis for further research determining the molecular targets of celastrol in AIH as well as drugs against other inflammatory and autoimmune diseases.

## Data Availability

The original contributions presented in the study are included in the article/Supplementary Materials, further inquiries can be directed to the corresponding authors.
