# Supplementary figures and images for: Celastrol Alleviates Autoimmune Hepatitis Through the PI3K/AKT Signaling Pathway Based on Network Pharmacology and Experiments
Source: Front Pharmacol. 2022 Mar 10;13:816350. doi: 10.3389/fphar.2022.816350 (PMC8960436; doi:10.3389/fphar.2022.816350)

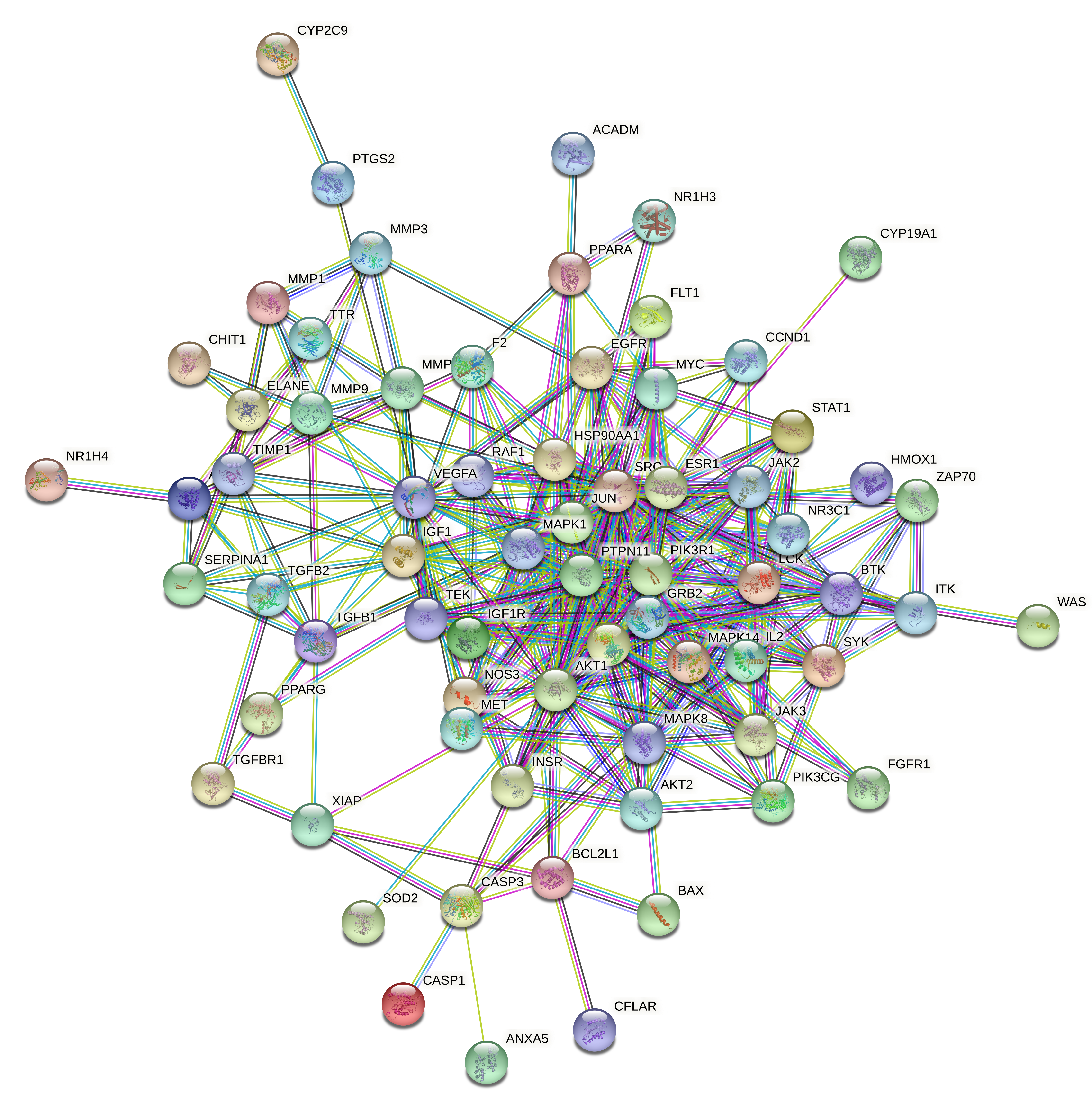

Supplement: Supplementary file 1 [file DataSheet3.ZIP › source data -1/cytoscape/string_hires_image (String ).png]

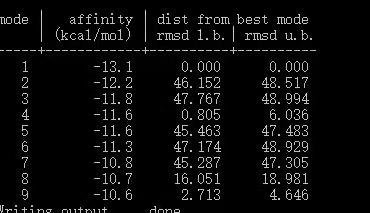

Supplement: Supplementary file 1 [file DataSheet3.ZIP › source data -1/docking/AKT1.jpg]

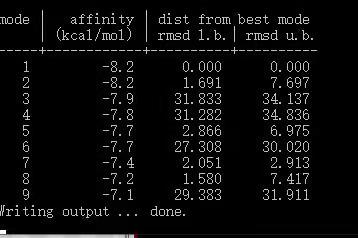

Supplement: Supplementary file 1 [file DataSheet3.ZIP › source data -1/docking/HRAS.jpg]

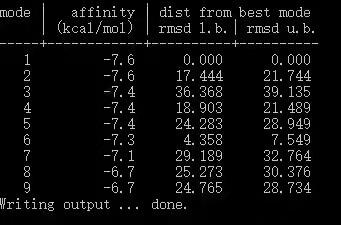

Supplement: Supplementary file 1 [file DataSheet3.ZIP › source data -1/docking/MAPK1.jpg]

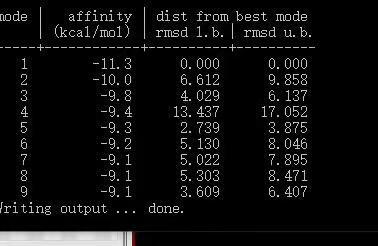

Supplement: Supplementary file 1 [file DataSheet3.ZIP › source data -1/docking/PIK3R1.jpg]

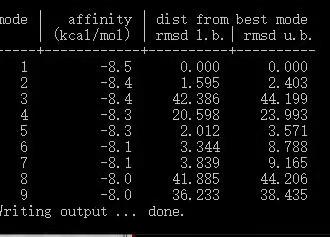

Supplement: Supplementary file 1 [file DataSheet3.ZIP › source data -1/docking/SRC.jpg]

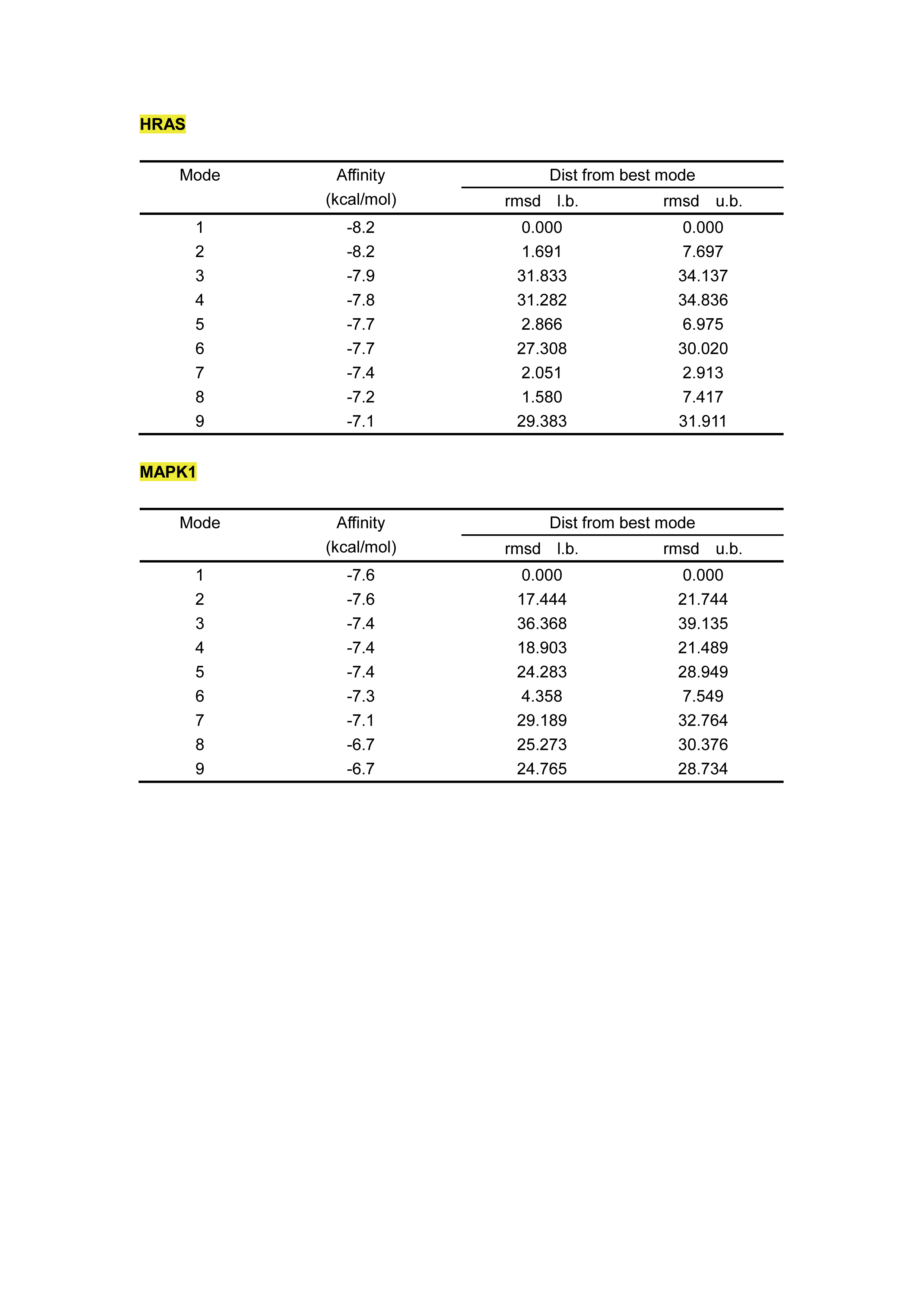

Supplement: Supplementary file 2 [file Image3.TIF]

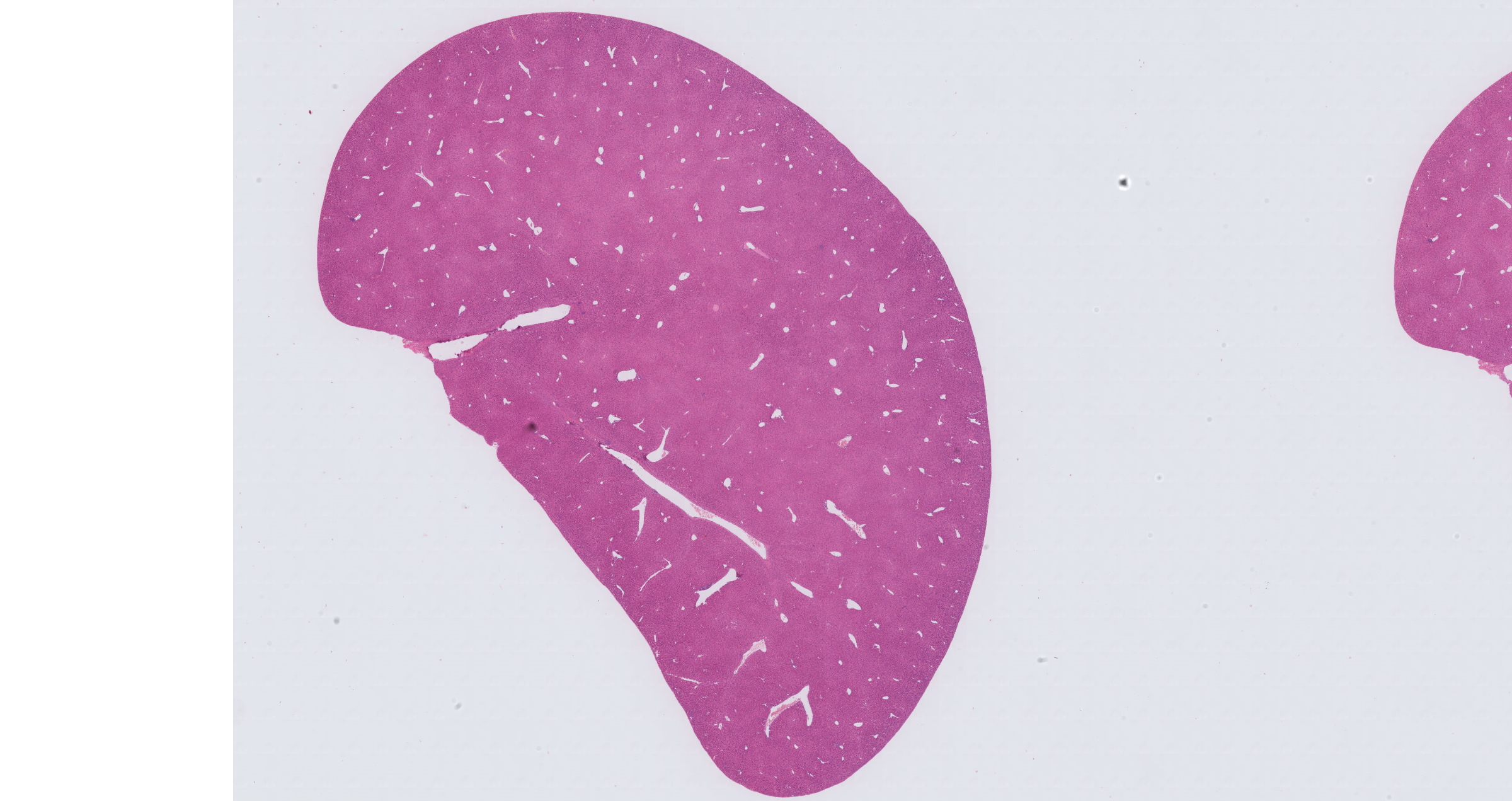

Supplement: Supplementary file 3 [file DataSheet4.ZIP › source data-2/Histology/cel(c) HE.jpg]

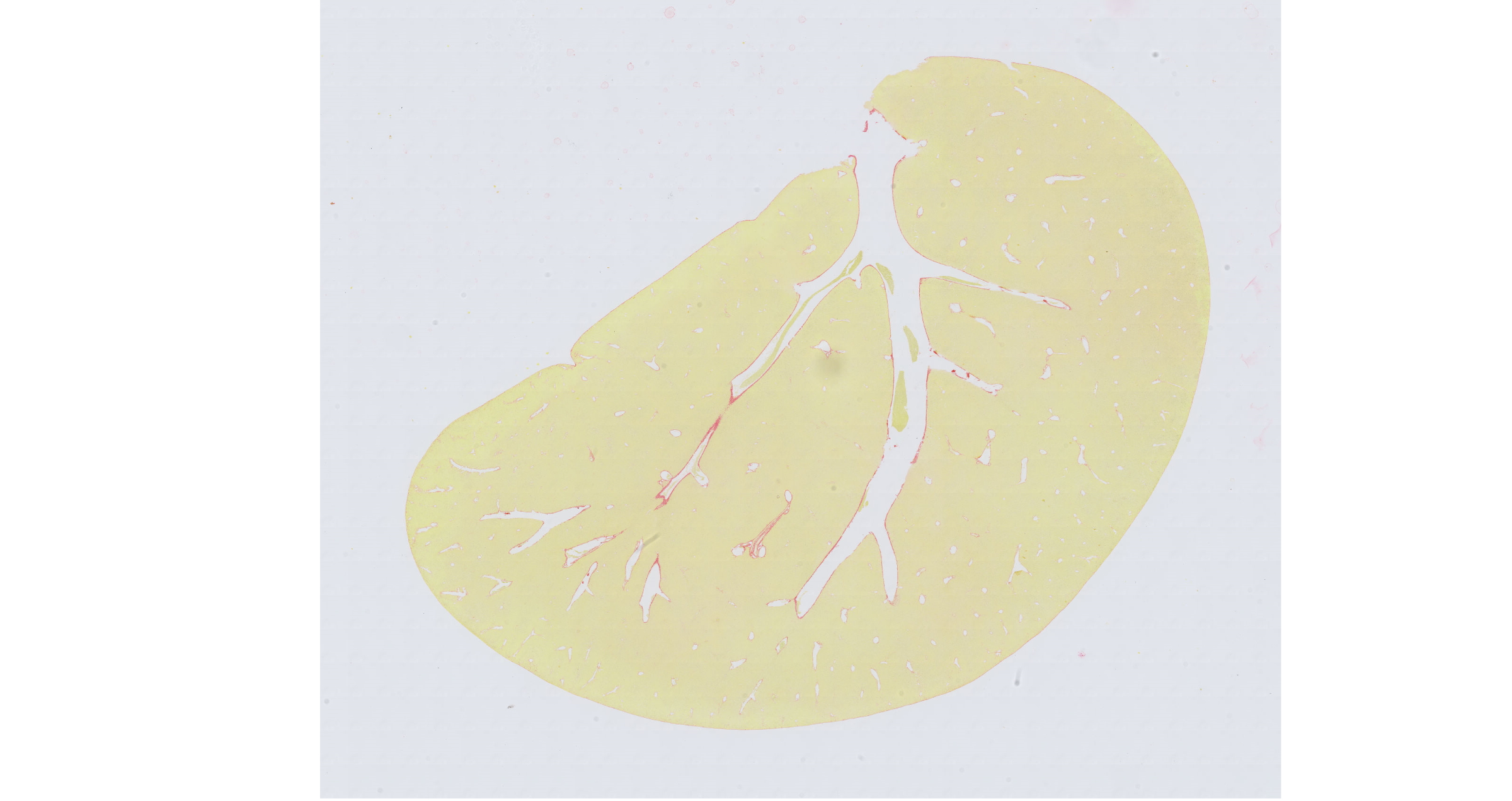

Supplement: Supplementary file 3 [file DataSheet4.ZIP › source data-2/Histology/cel(c).jpg]

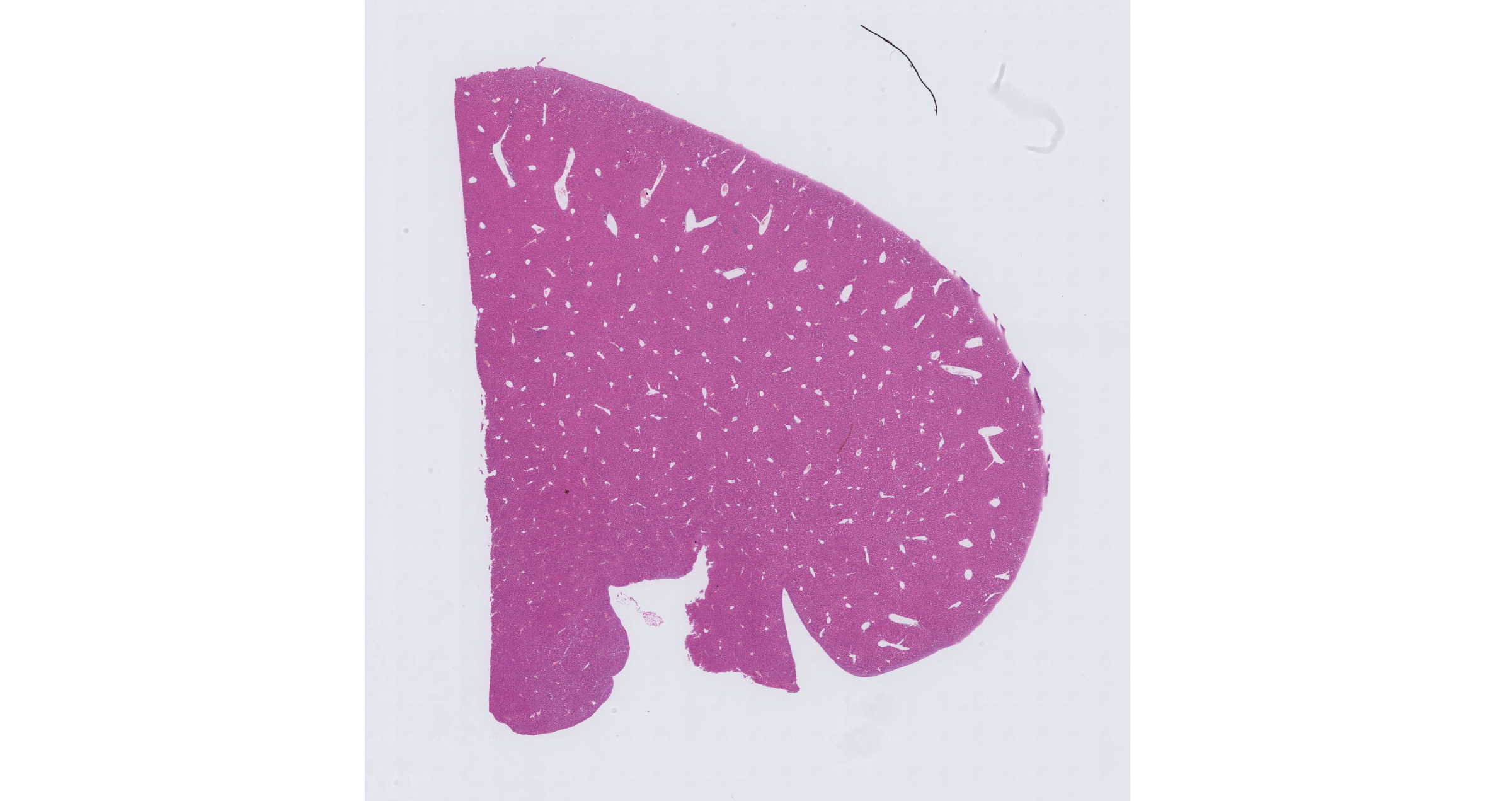

Supplement: Supplementary file 3 [file DataSheet4.ZIP › source data-2/Histology/Cel.jpg]

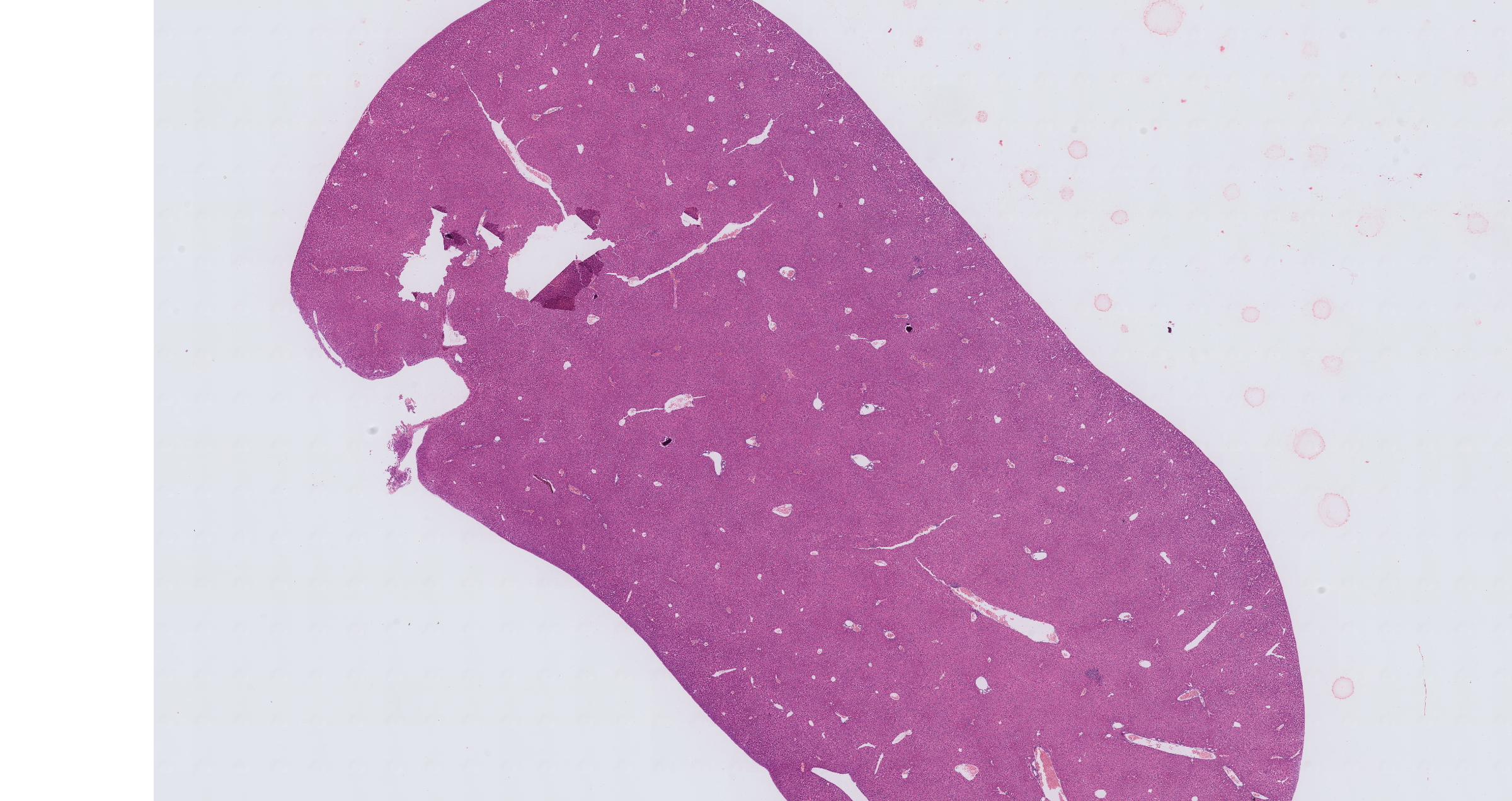

Supplement: Supplementary file 3 [file DataSheet4.ZIP › source data-2/Histology/conA+Cel.jpg]

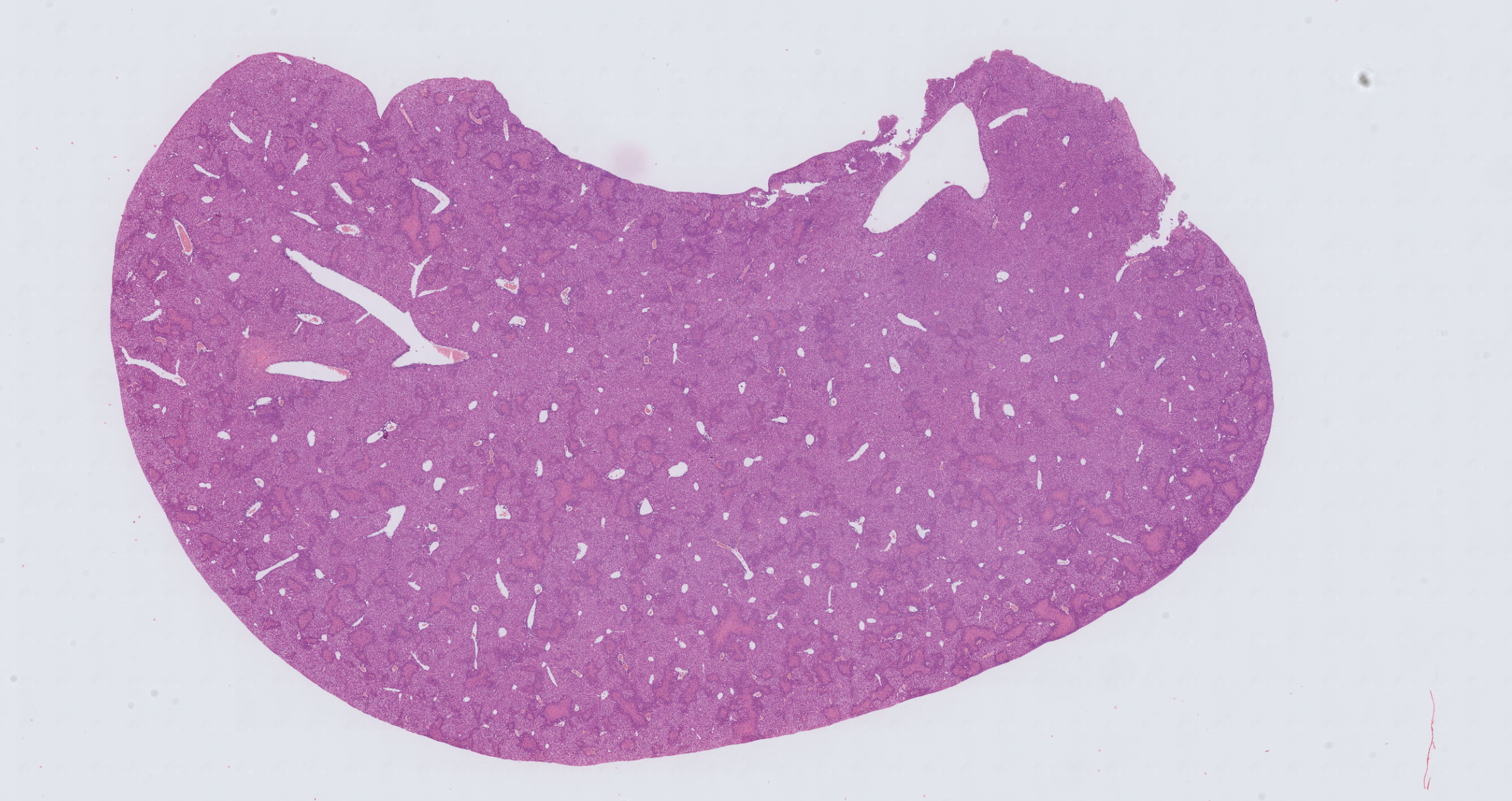

Supplement: Supplementary file 3 [file DataSheet4.ZIP › source data-2/Histology/ConA.jpg]

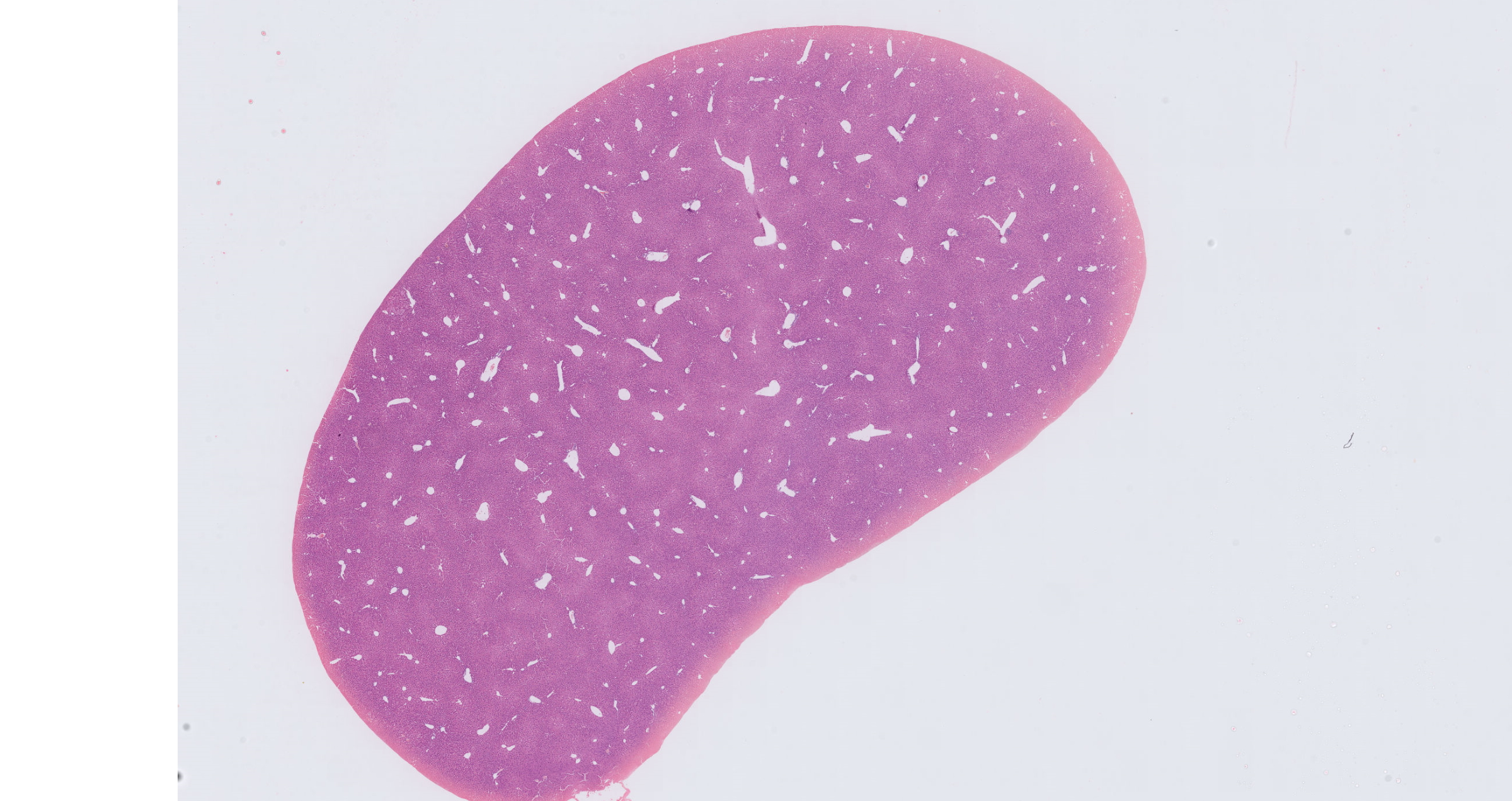

Supplement: Supplementary file 3 [file DataSheet4.ZIP › source data-2/Histology/control-c HE.jpg]

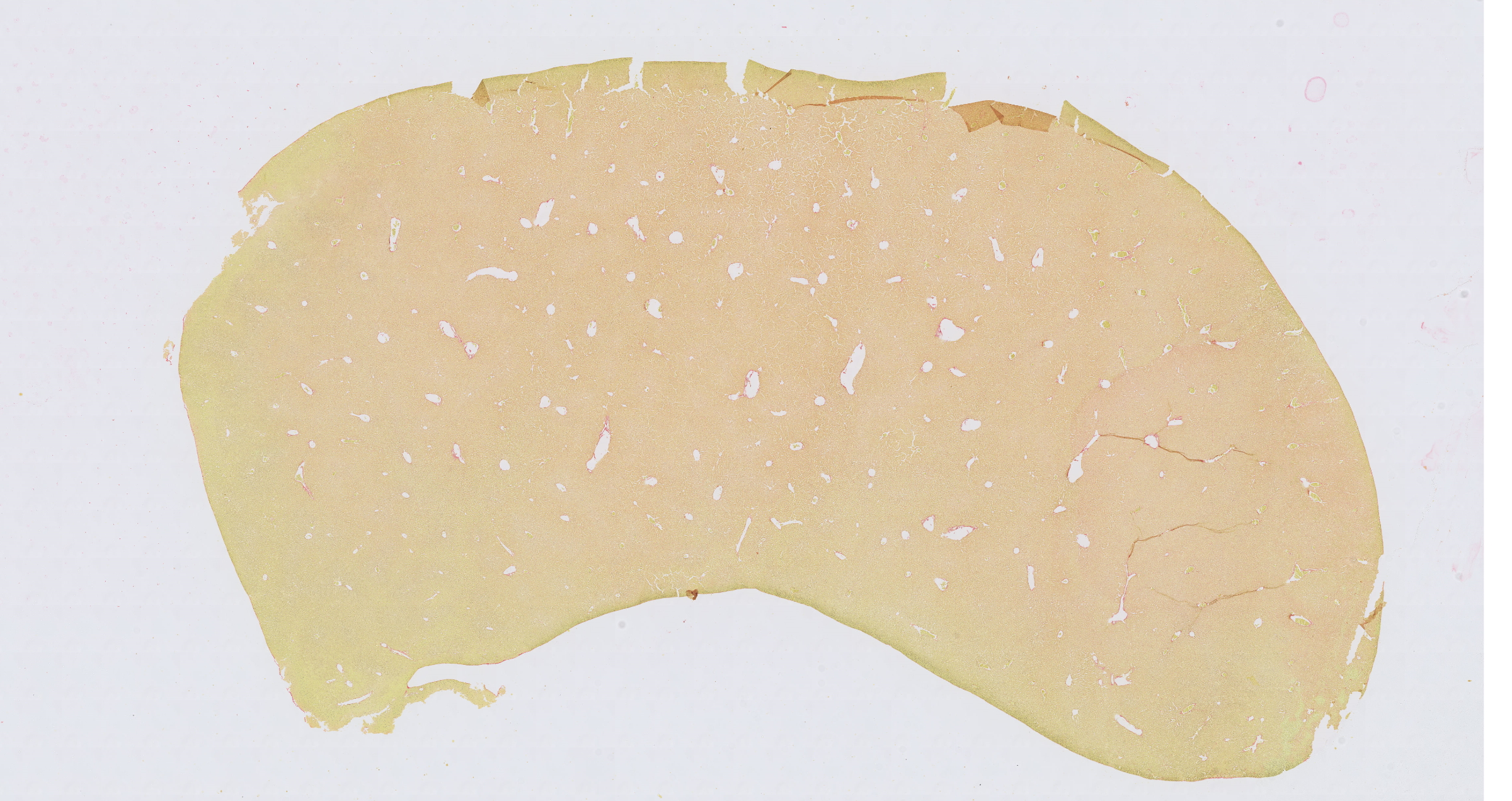

Supplement: Supplementary file 3 [file DataSheet4.ZIP › source data-2/Histology/control-c.jpg]

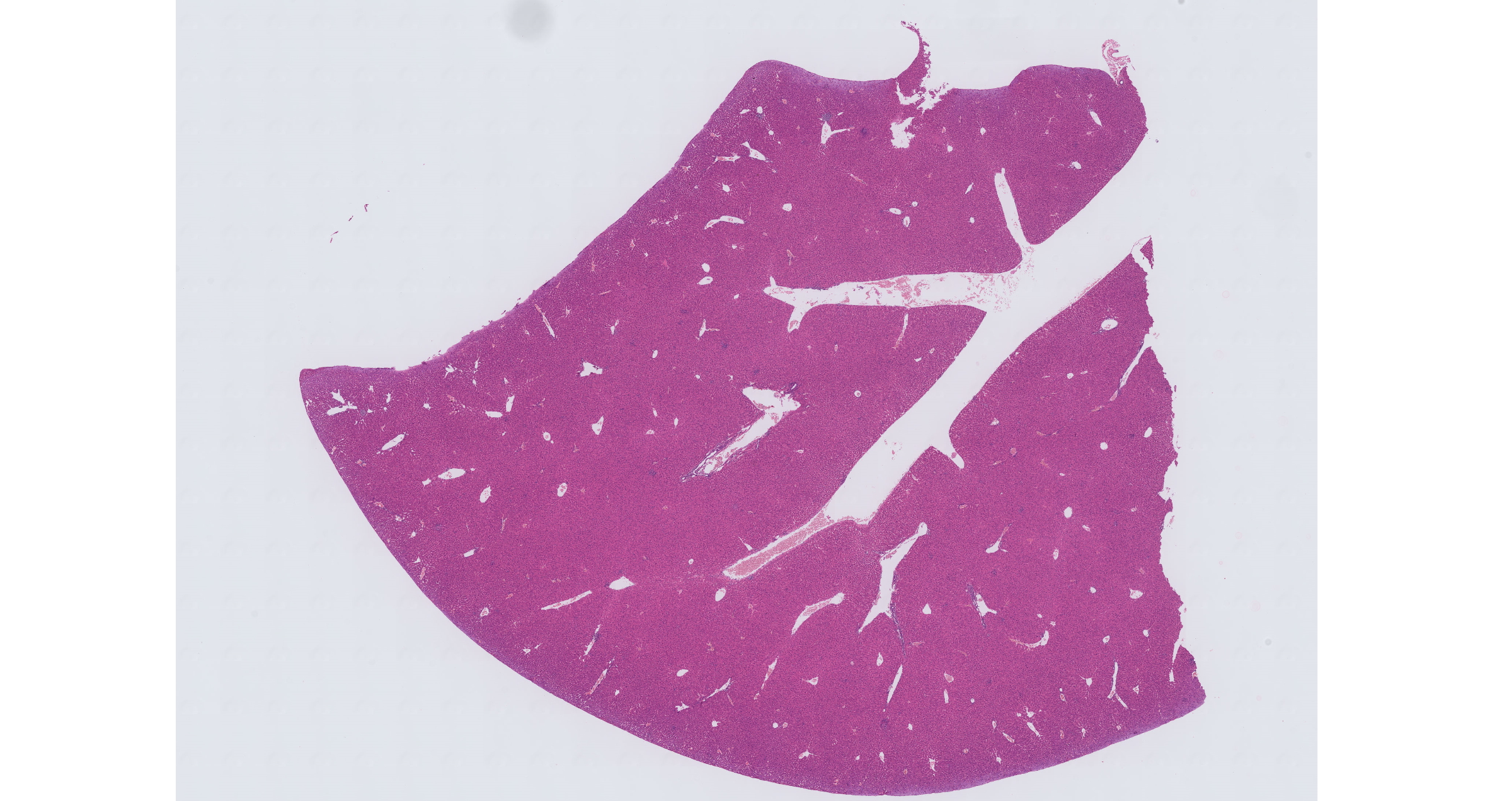

Supplement: Supplementary file 3 [file DataSheet4.ZIP › source data-2/Histology/control.jpg]

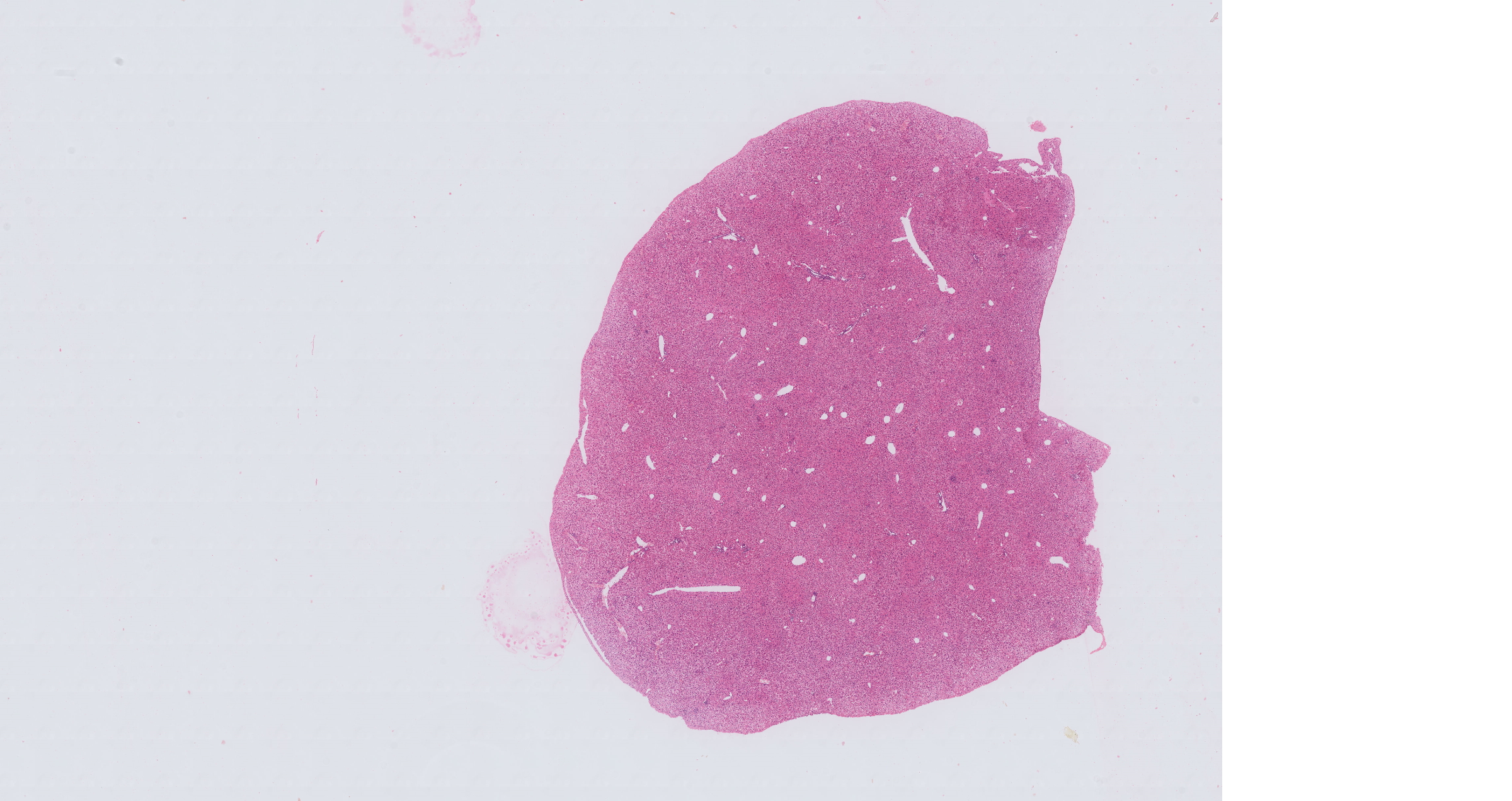

Supplement: Supplementary file 3 [file DataSheet4.ZIP › source data-2/Histology/CYP HE.jpg]

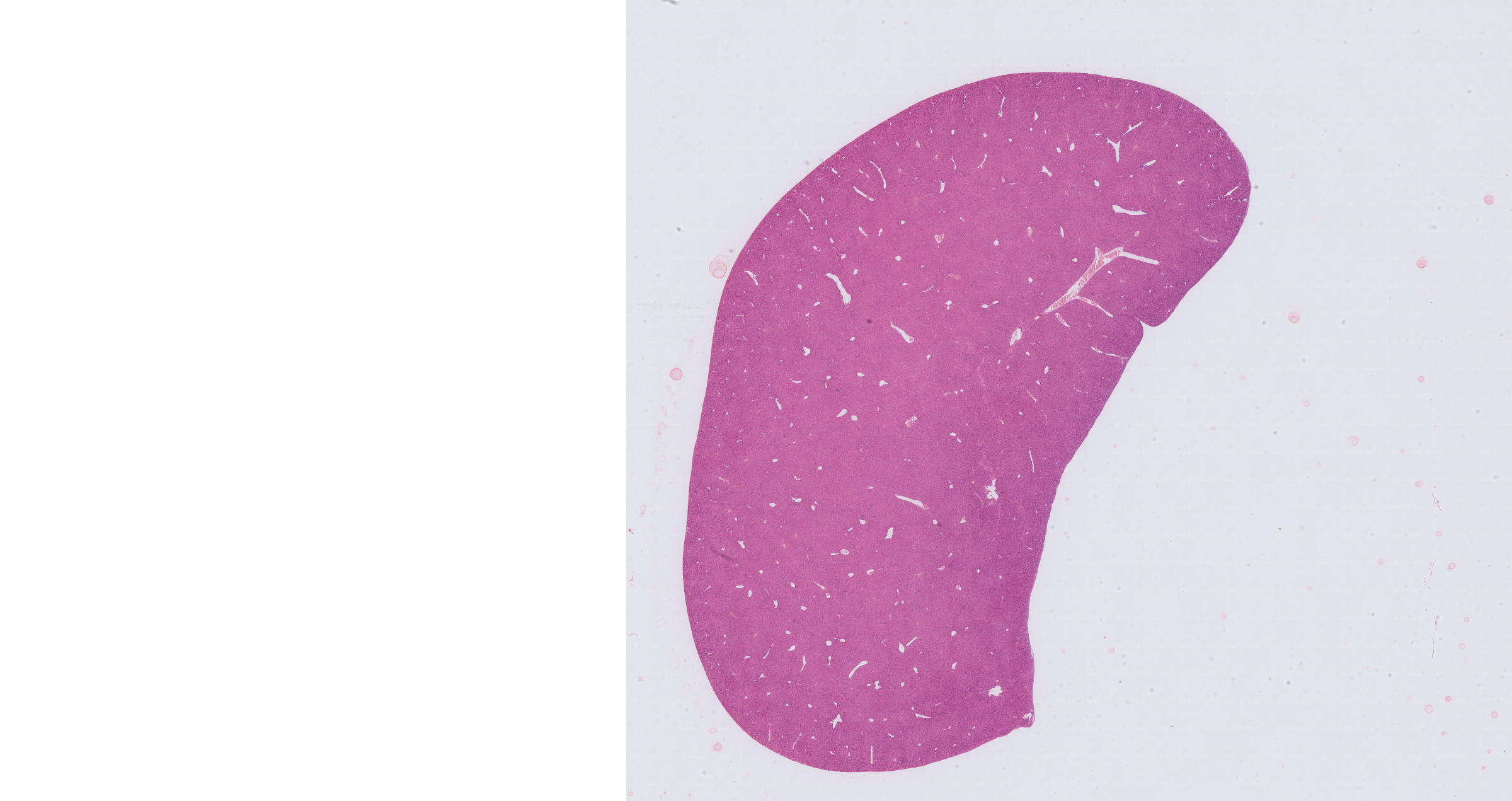

Supplement: Supplementary file 3 [file DataSheet4.ZIP › source data-2/Histology/cyp+cel HE.jpg]

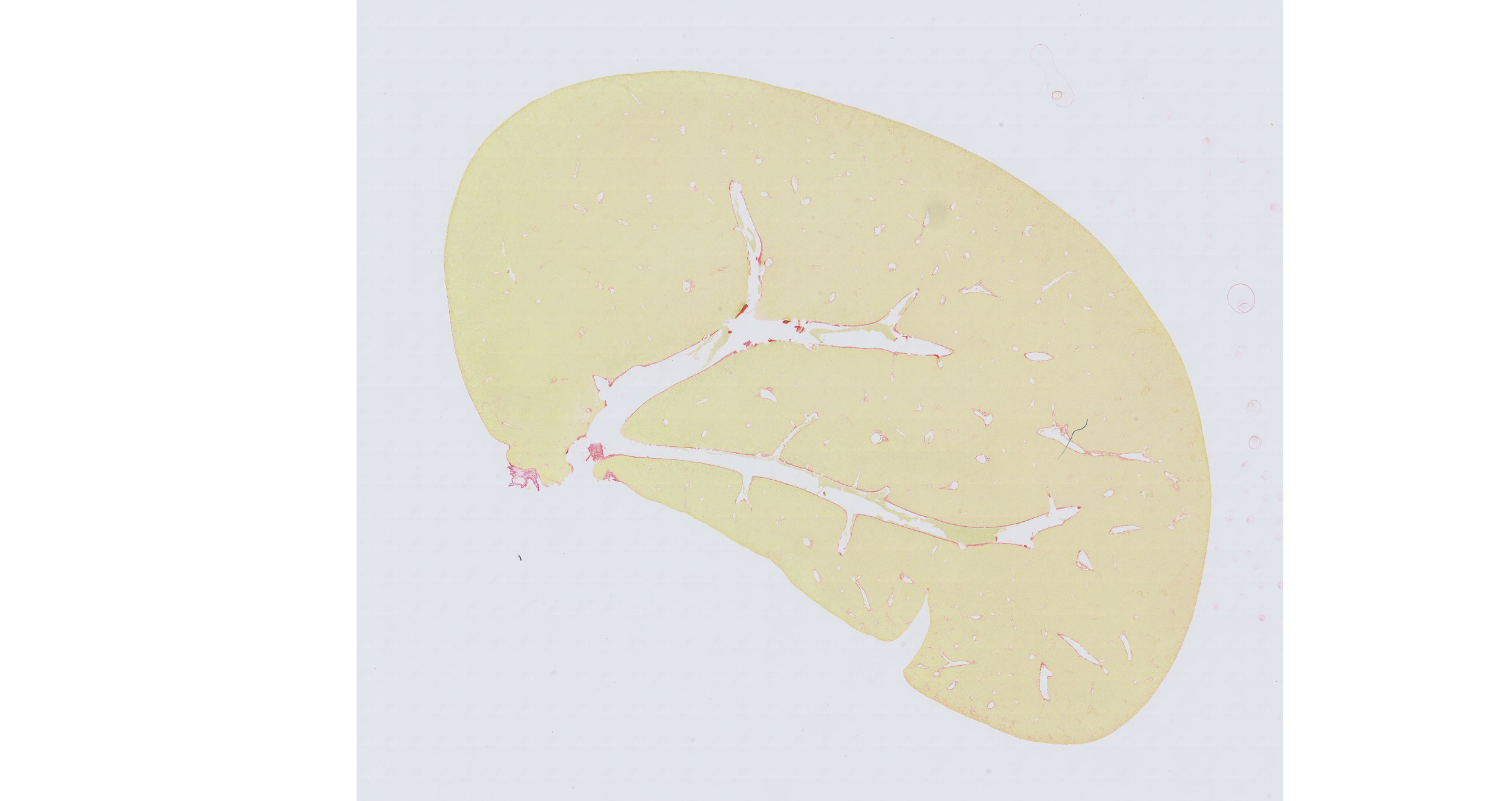

Supplement: Supplementary file 3 [file DataSheet4.ZIP › source data-2/Histology/cyp+cel.jpg]

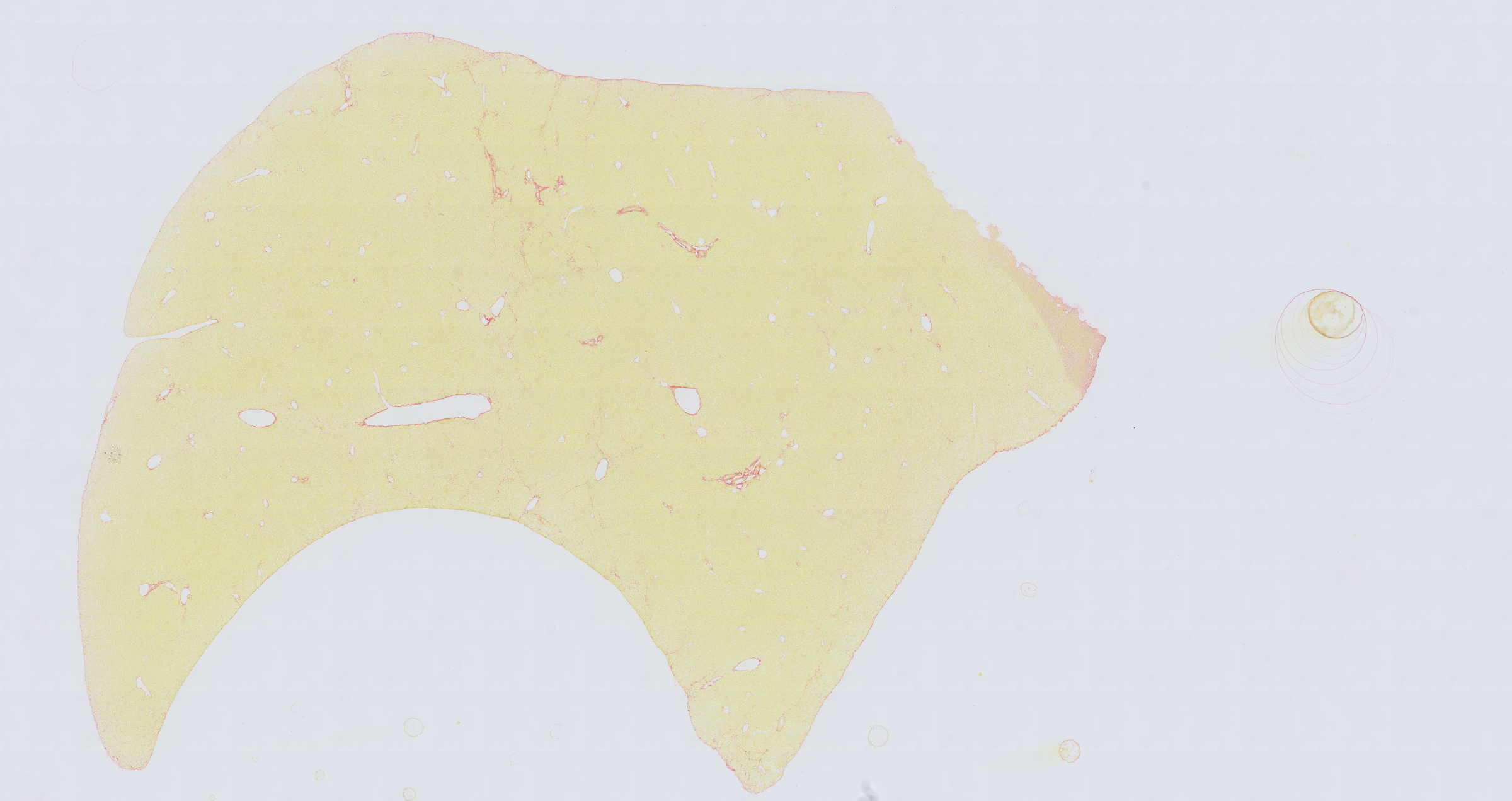

Supplement: Supplementary file 3 [file DataSheet4.ZIP › source data-2/Histology/CYP.jpg]

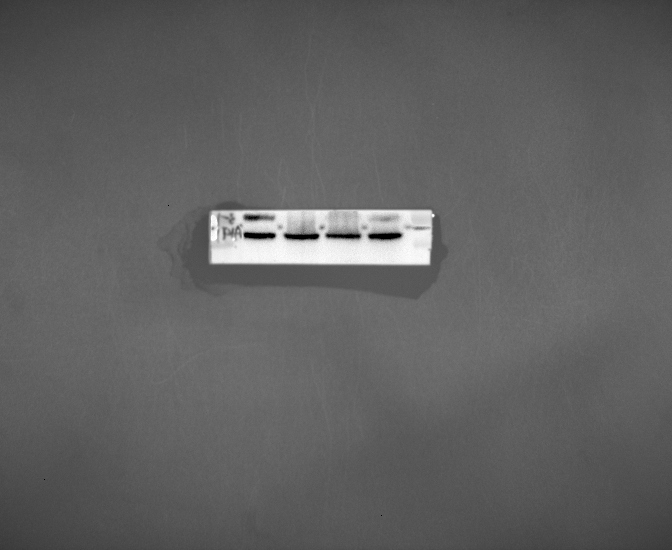

Supplement: Supplementary file 3 [file DataSheet4.ZIP › source data-2/WB-original gels/AIH models/AKT1-0.tif]

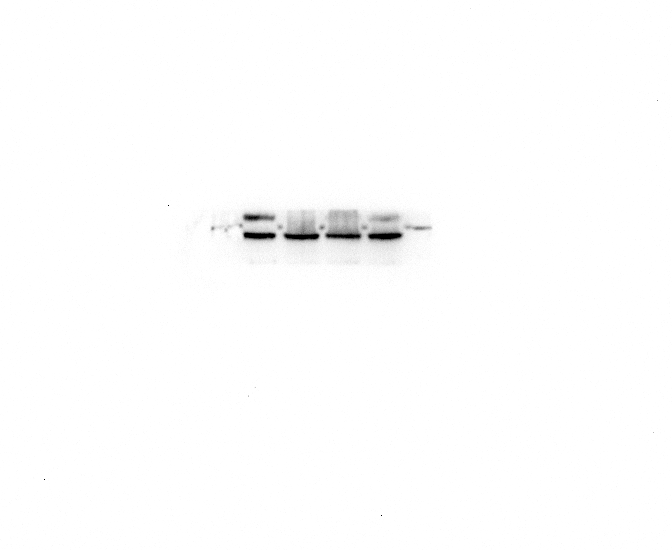

Supplement: Supplementary file 3 [file DataSheet4.ZIP › source data-2/WB-original gels/AIH models/AKT1.tif]

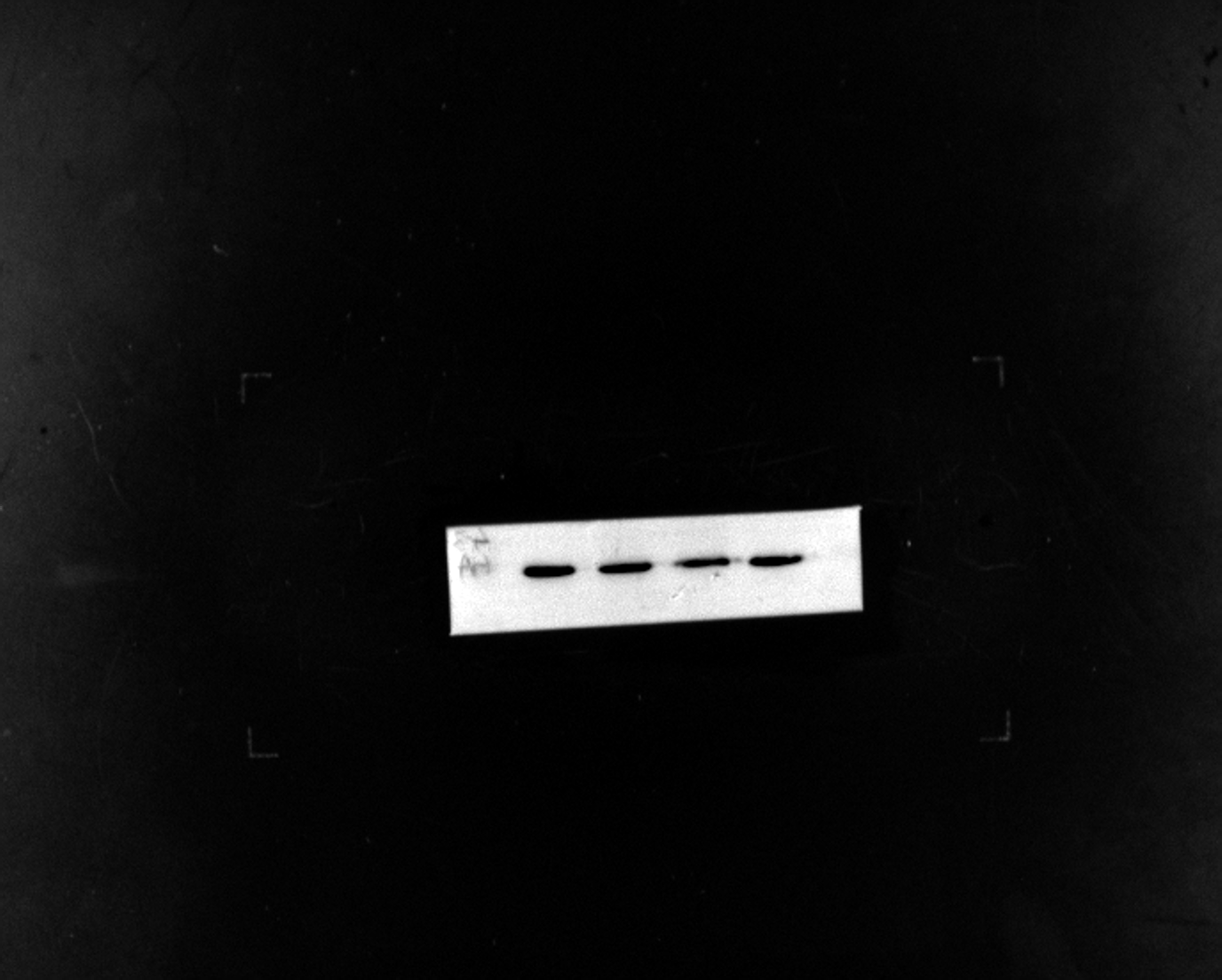

Supplement: Supplementary file 3 [file DataSheet4.ZIP › source data-2/WB-original gels/AIH models/GAPDH-0.Tif]

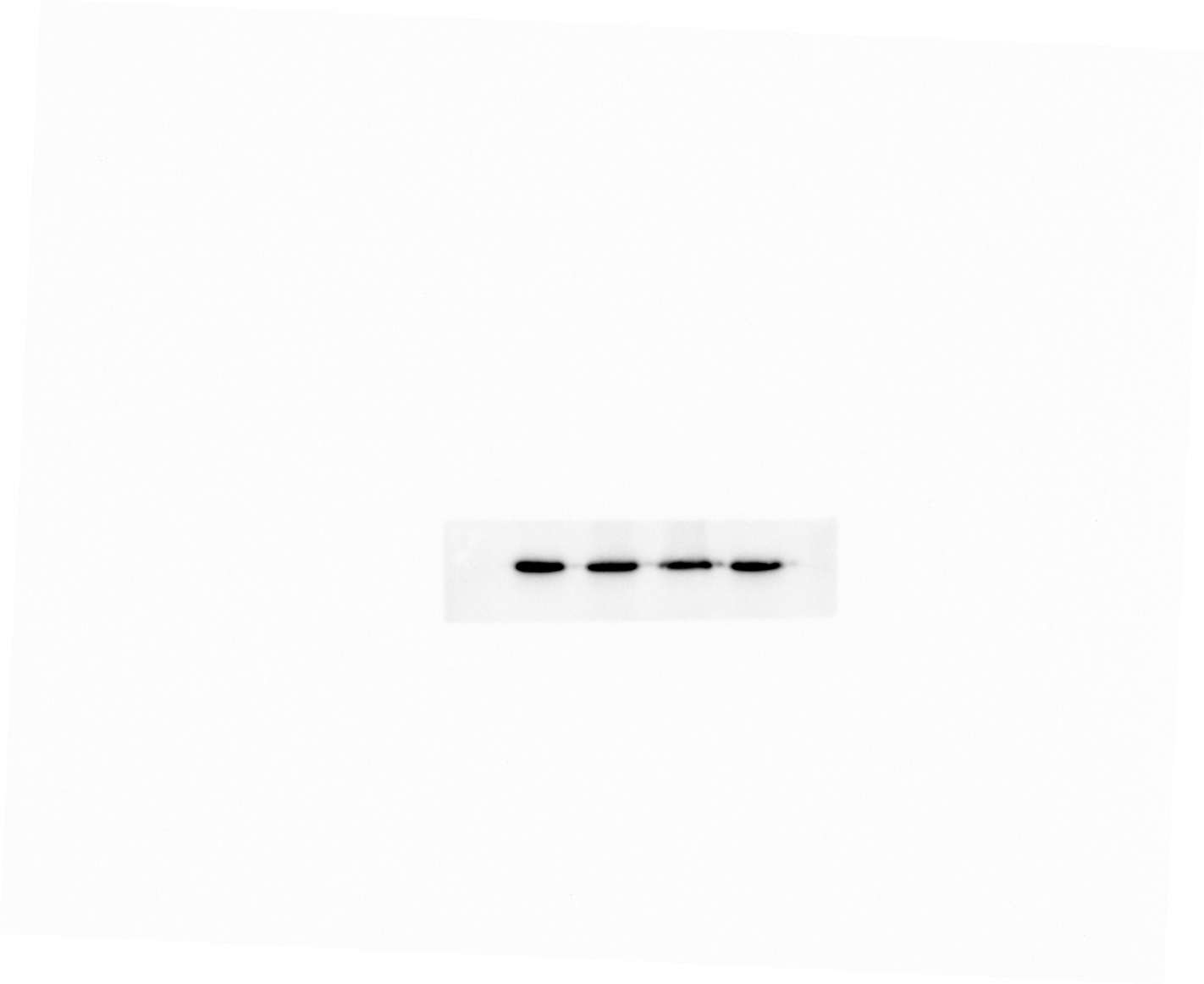

Supplement: Supplementary file 3 [file DataSheet4.ZIP › source data-2/WB-original gels/AIH models/GAPDH.Tif]

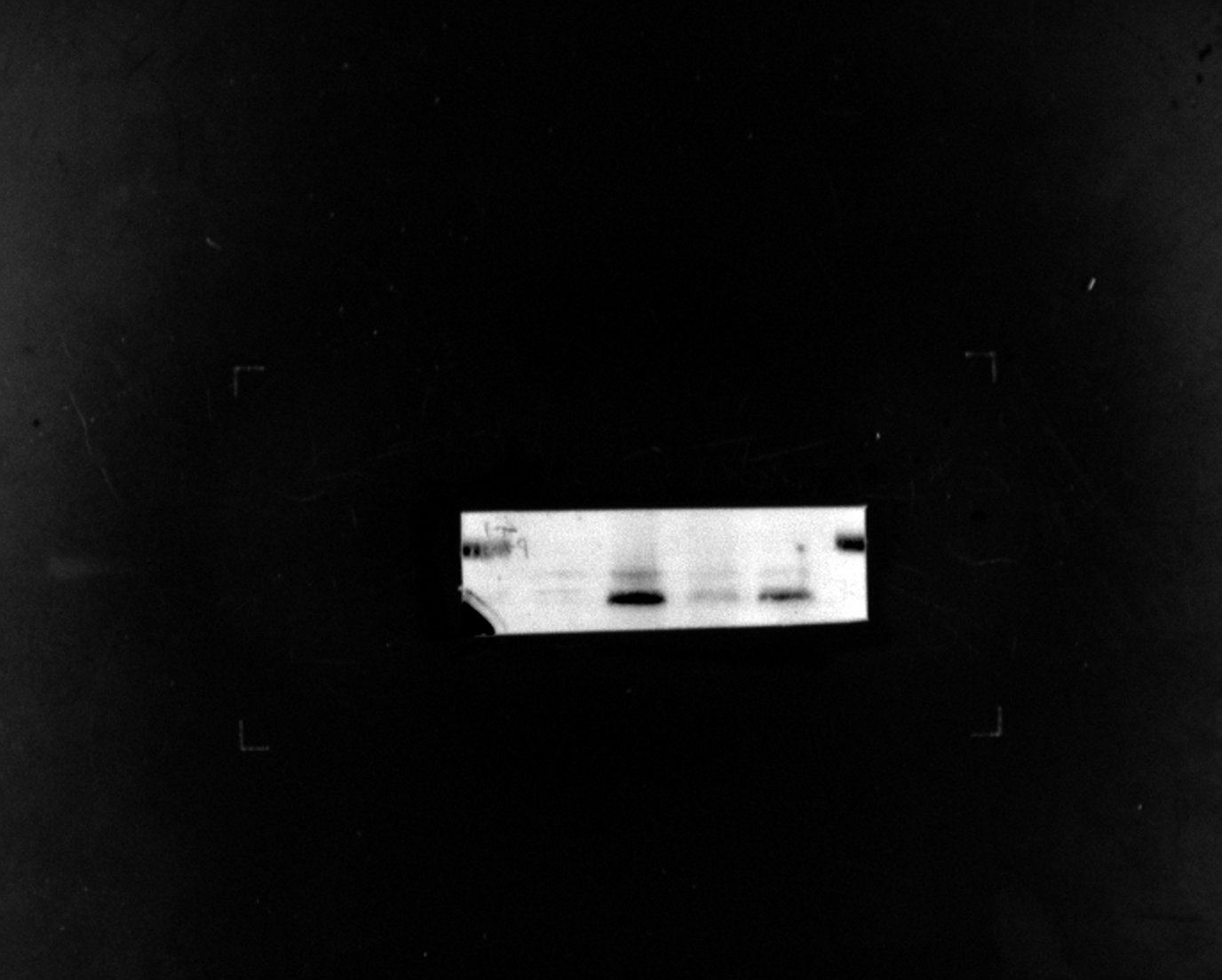

Supplement: Supplementary file 3 [file DataSheet4.ZIP › source data-2/WB-original gels/AIH models/P-AKT1-0.Tif]

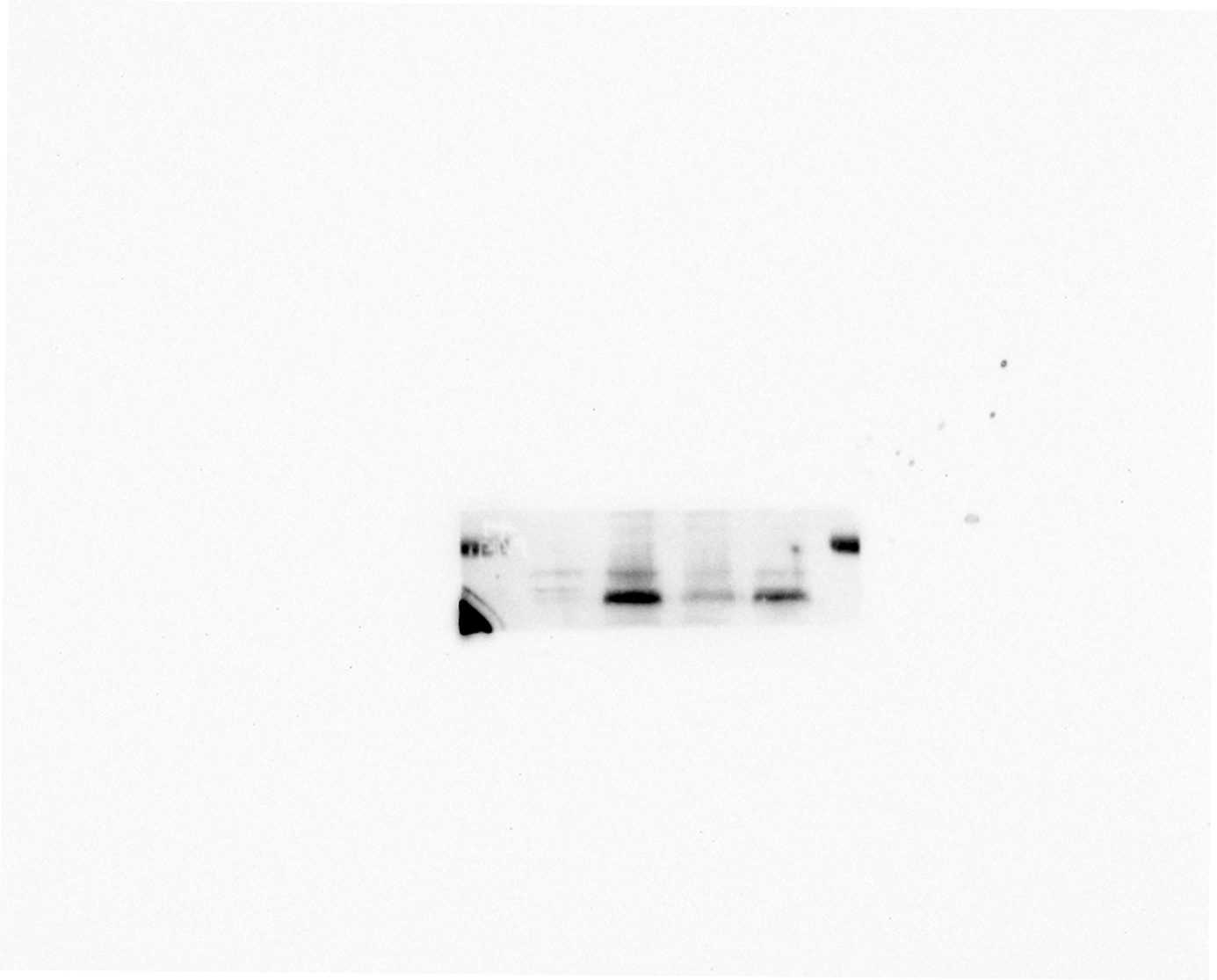

Supplement: Supplementary file 3 [file DataSheet4.ZIP › source data-2/WB-original gels/AIH models/P-AKT1.Tif]

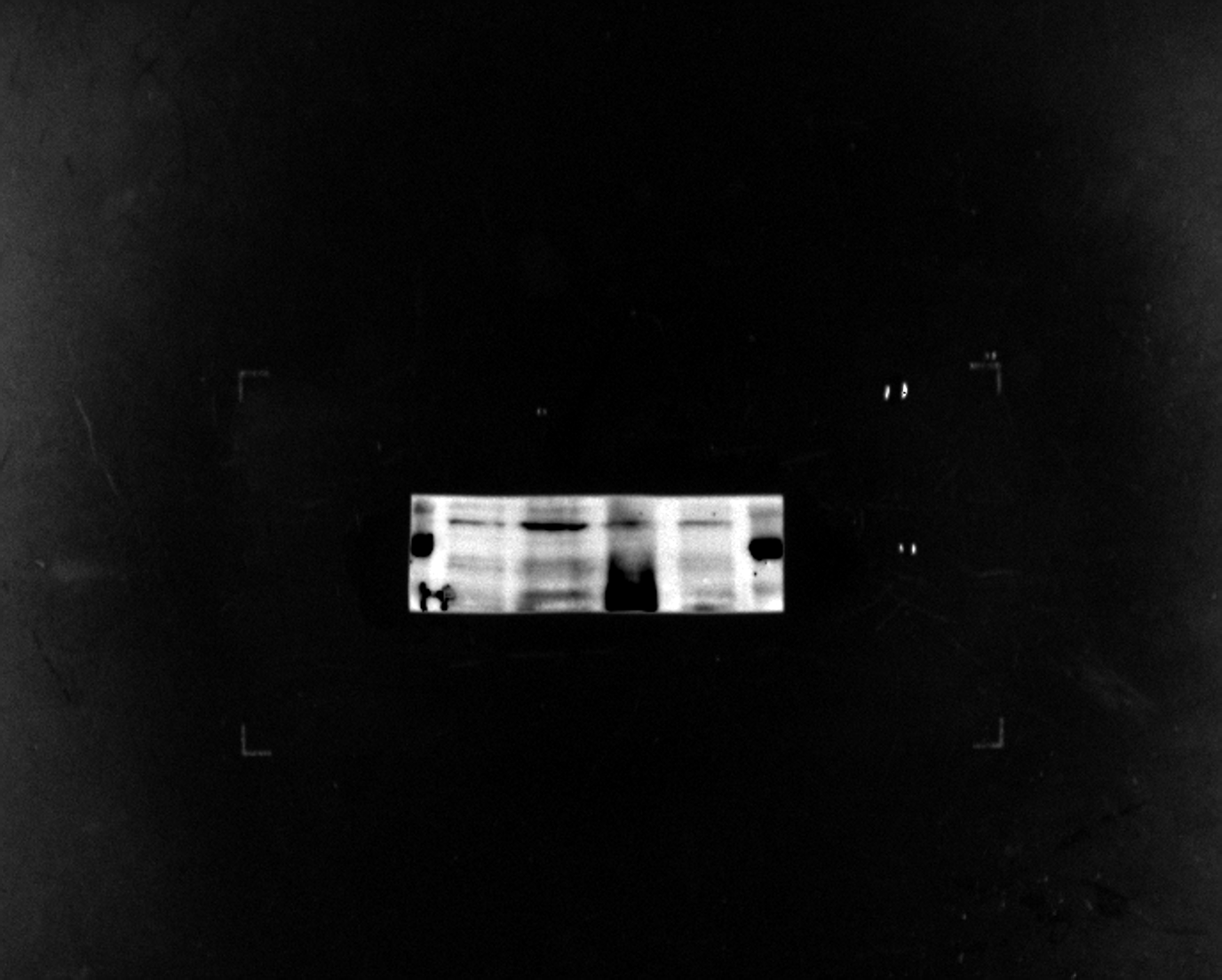

Supplement: Supplementary file 3 [file DataSheet4.ZIP › source data-2/WB-original gels/AIH models/P-PI3K-0.Tif]

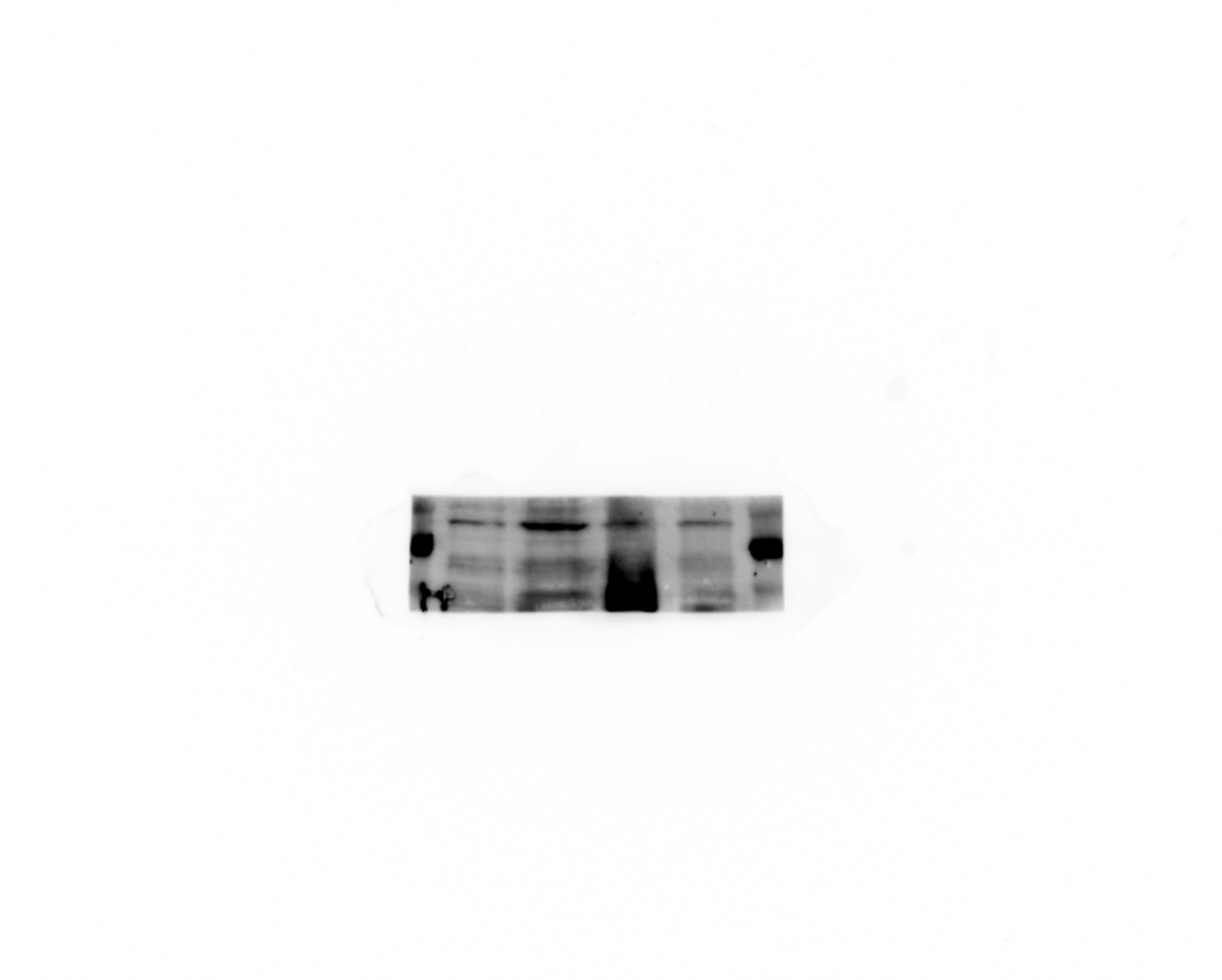

Supplement: Supplementary file 3 [file DataSheet4.ZIP › source data-2/WB-original gels/AIH models/P-PI3K.Tif]

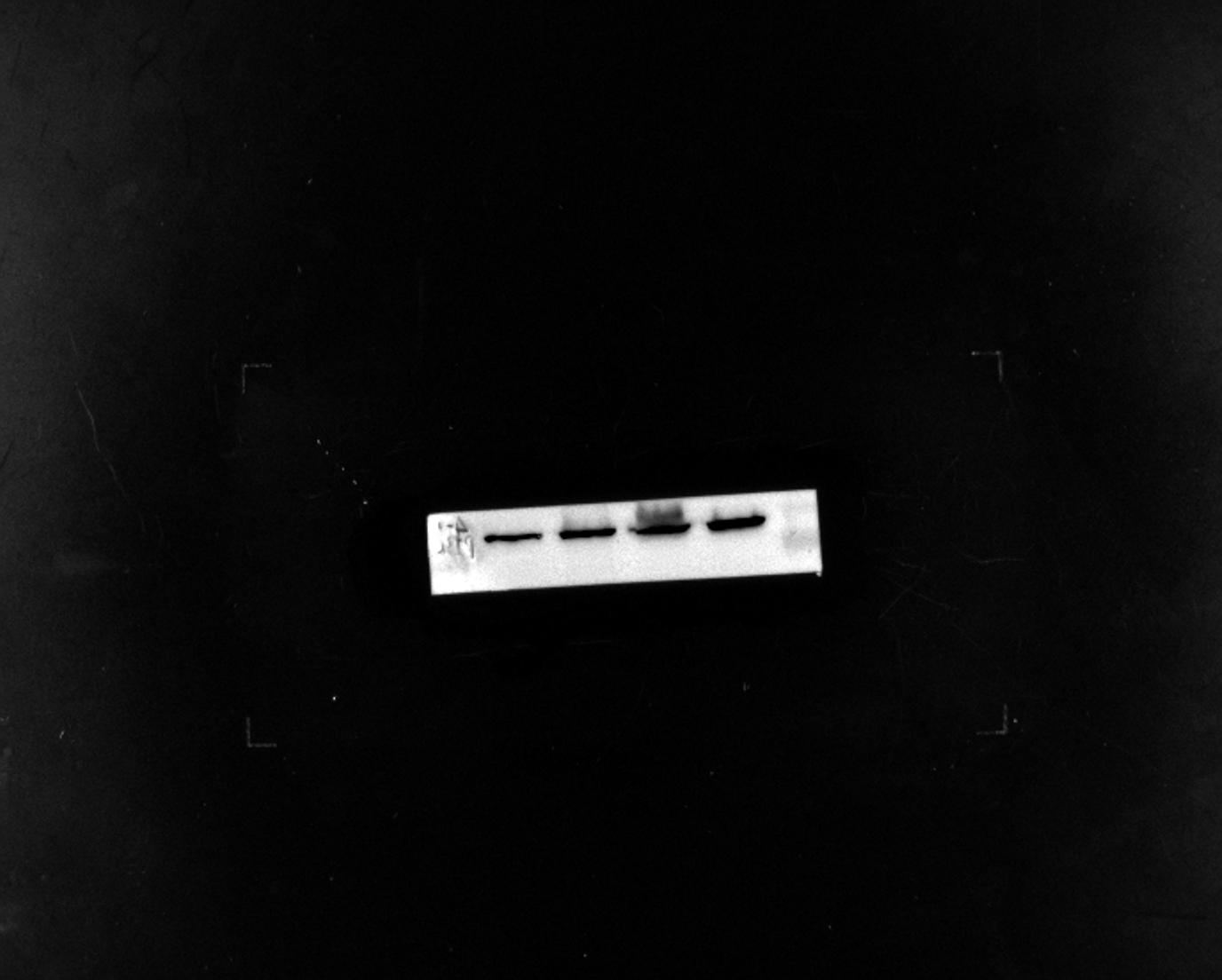

Supplement: Supplementary file 3 [file DataSheet4.ZIP › source data-2/WB-original gels/AIH models/PI3K-0.Tif]

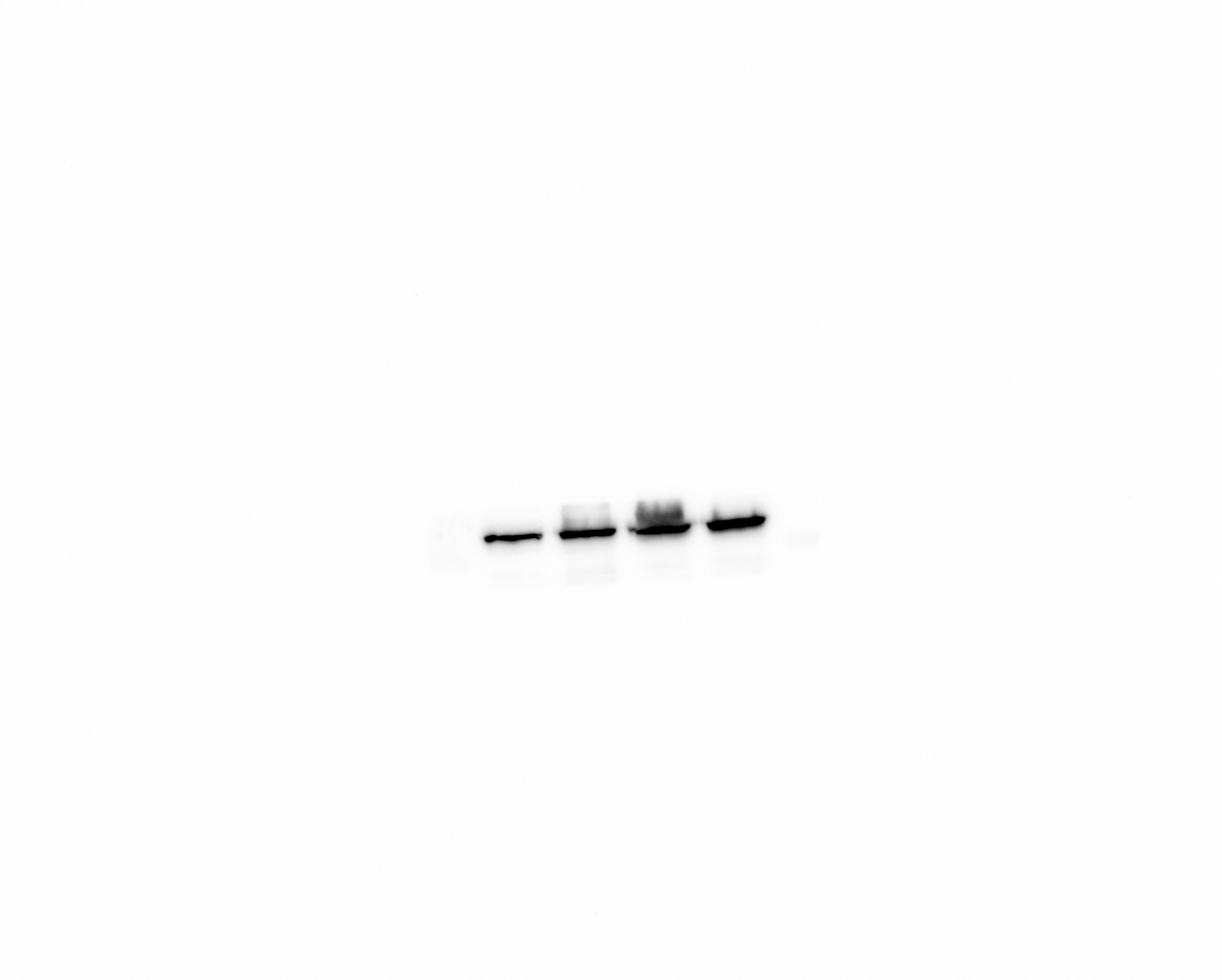

Supplement: Supplementary file 3 [file DataSheet4.ZIP › source data-2/WB-original gels/AIH models/PI3K.Tif]

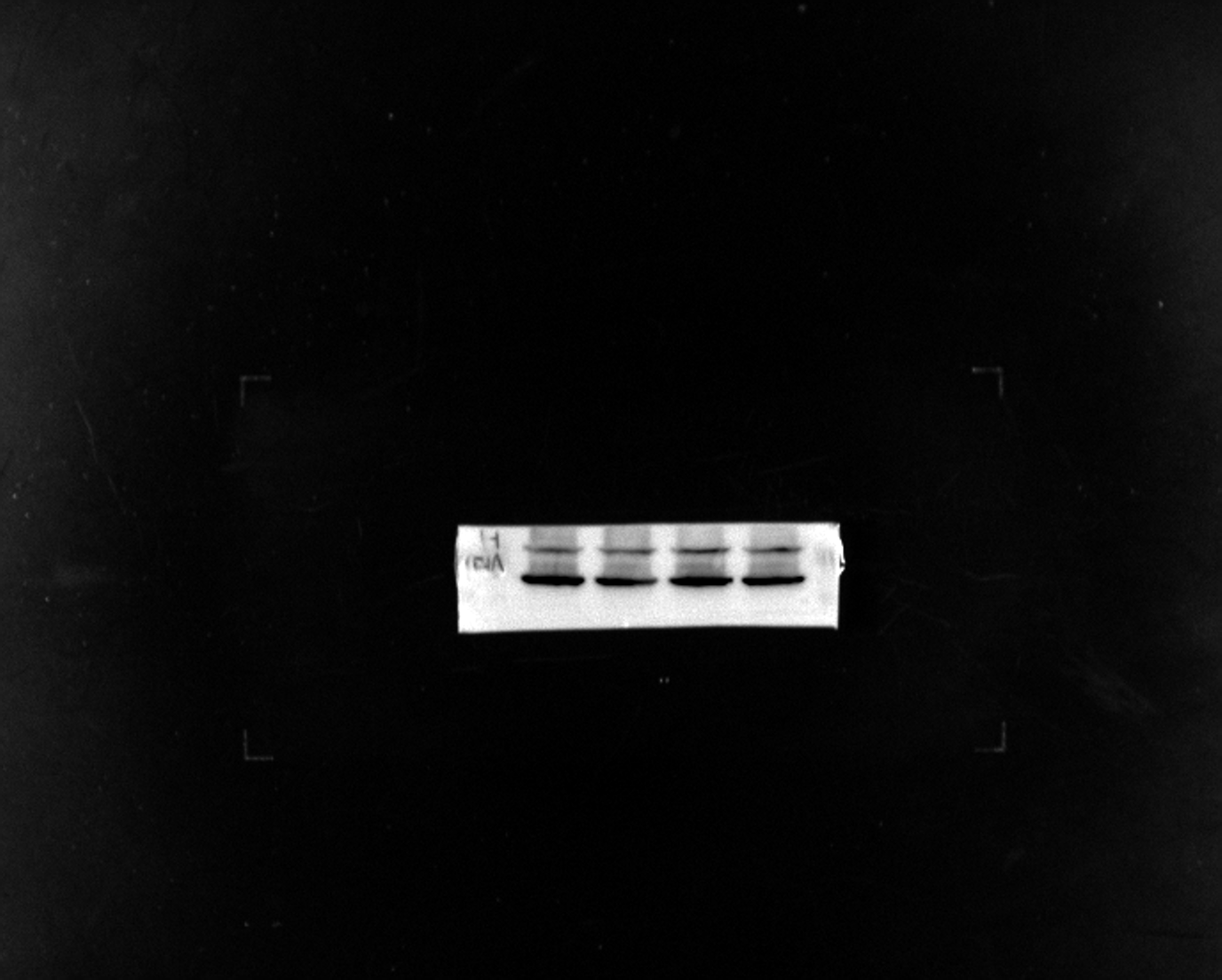

Supplement: Supplementary file 3 [file DataSheet4.ZIP › source data-2/WB-original gels/ConA models/AKT1-0.Tif]

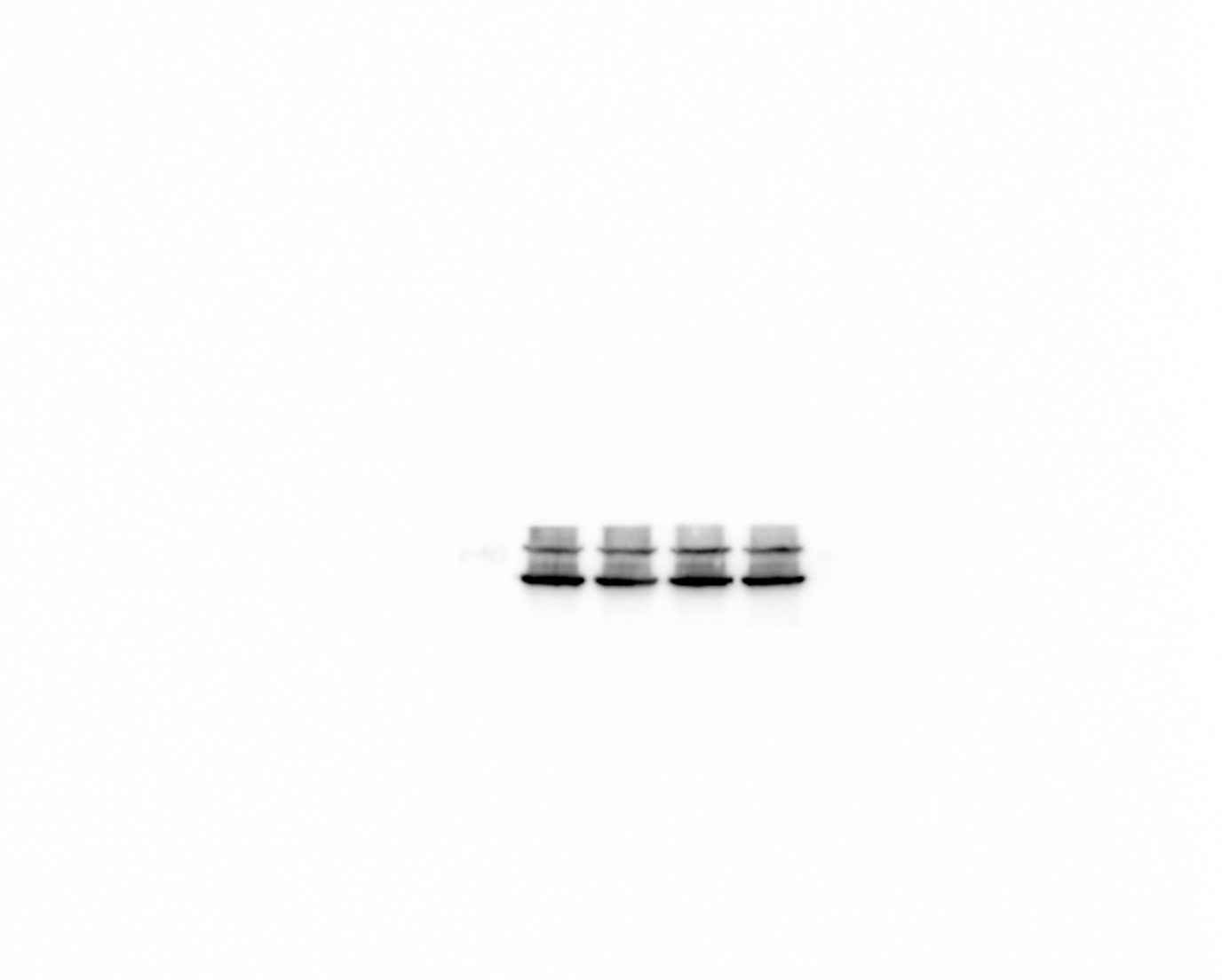

Supplement: Supplementary file 3 [file DataSheet4.ZIP › source data-2/WB-original gels/ConA models/AKT1.Tif]

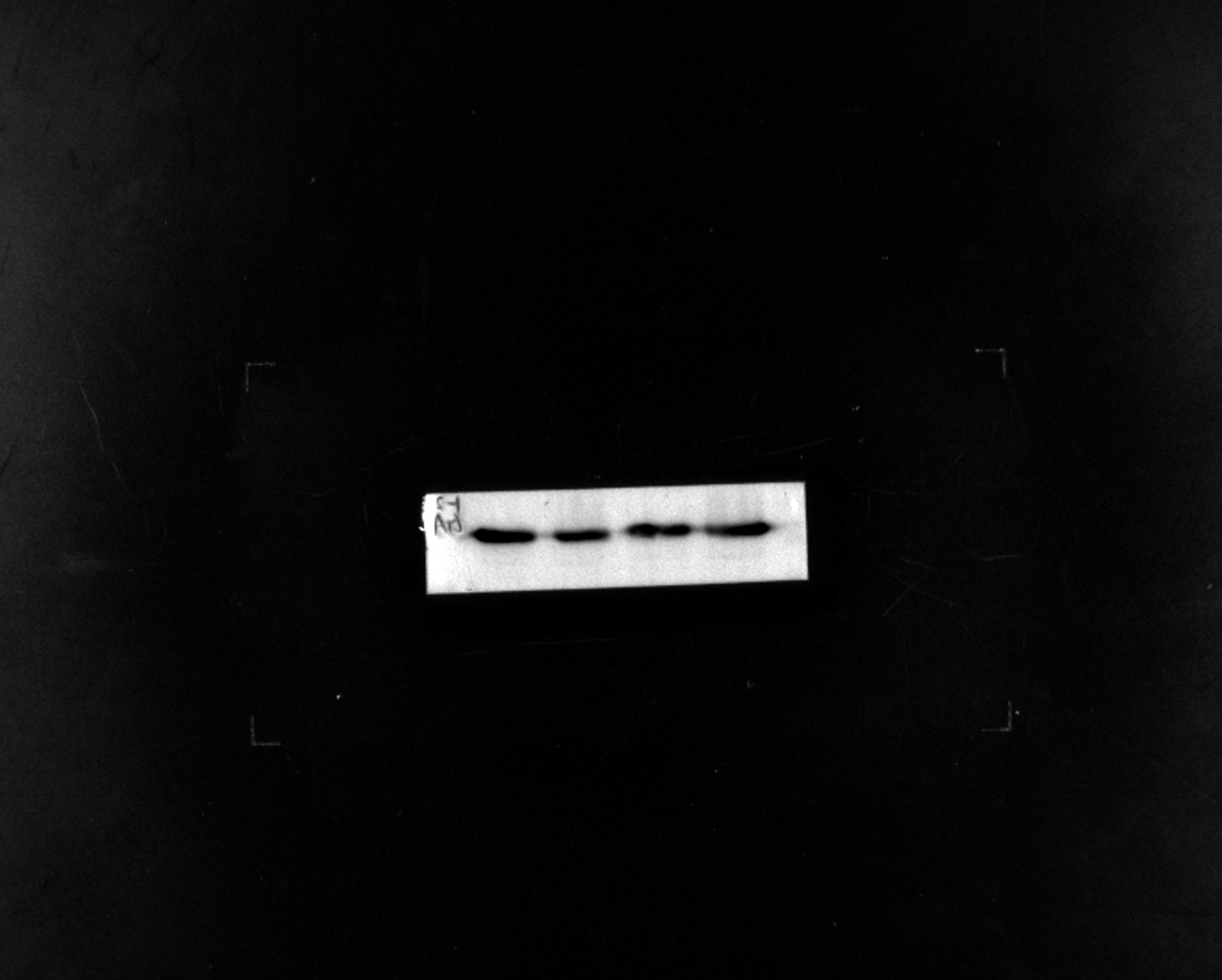

Supplement: Supplementary file 3 [file DataSheet4.ZIP › source data-2/WB-original gels/ConA models/GAPDH-0.Tif]

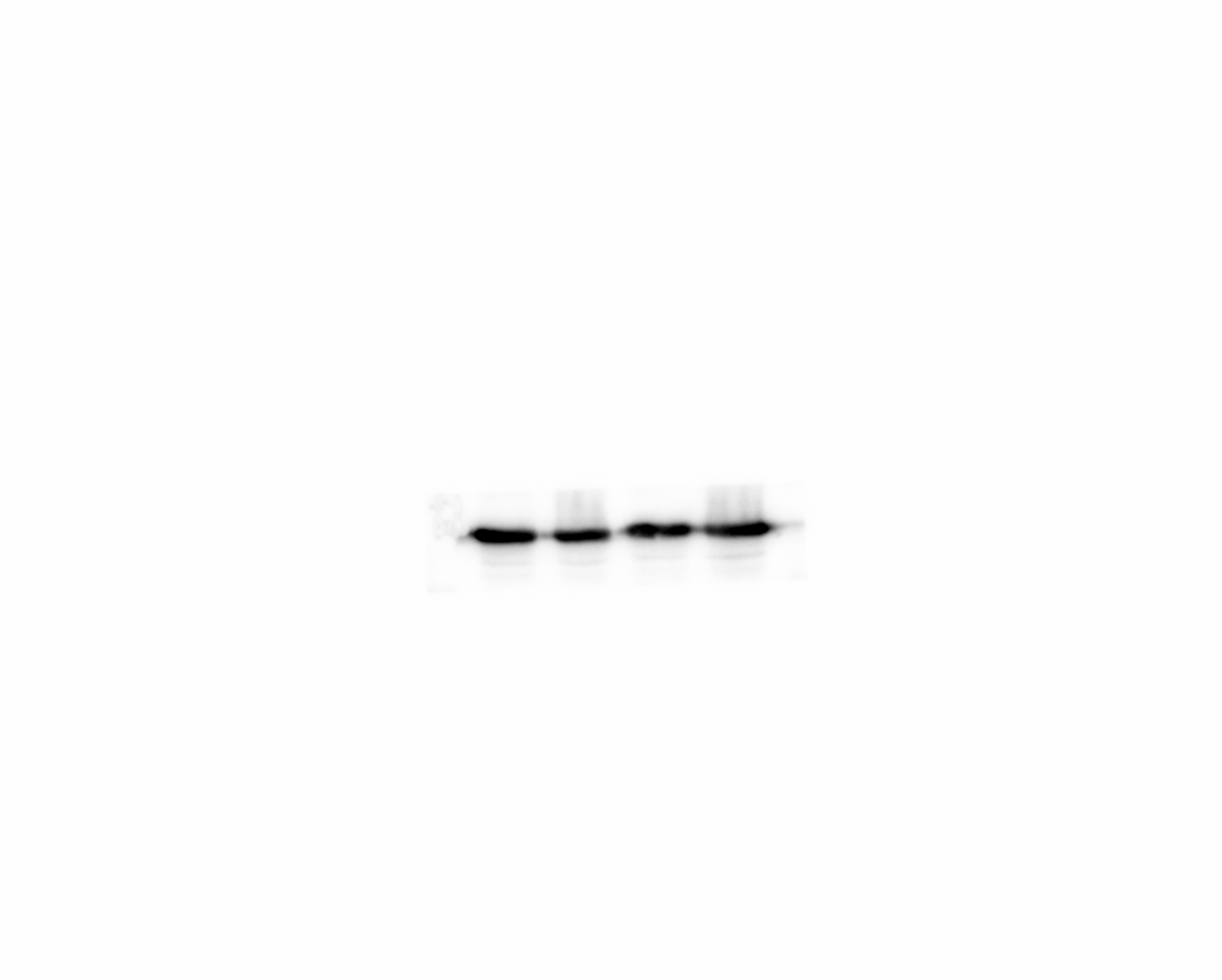

Supplement: Supplementary file 3 [file DataSheet4.ZIP › source data-2/WB-original gels/ConA models/GAPDH.Tif]

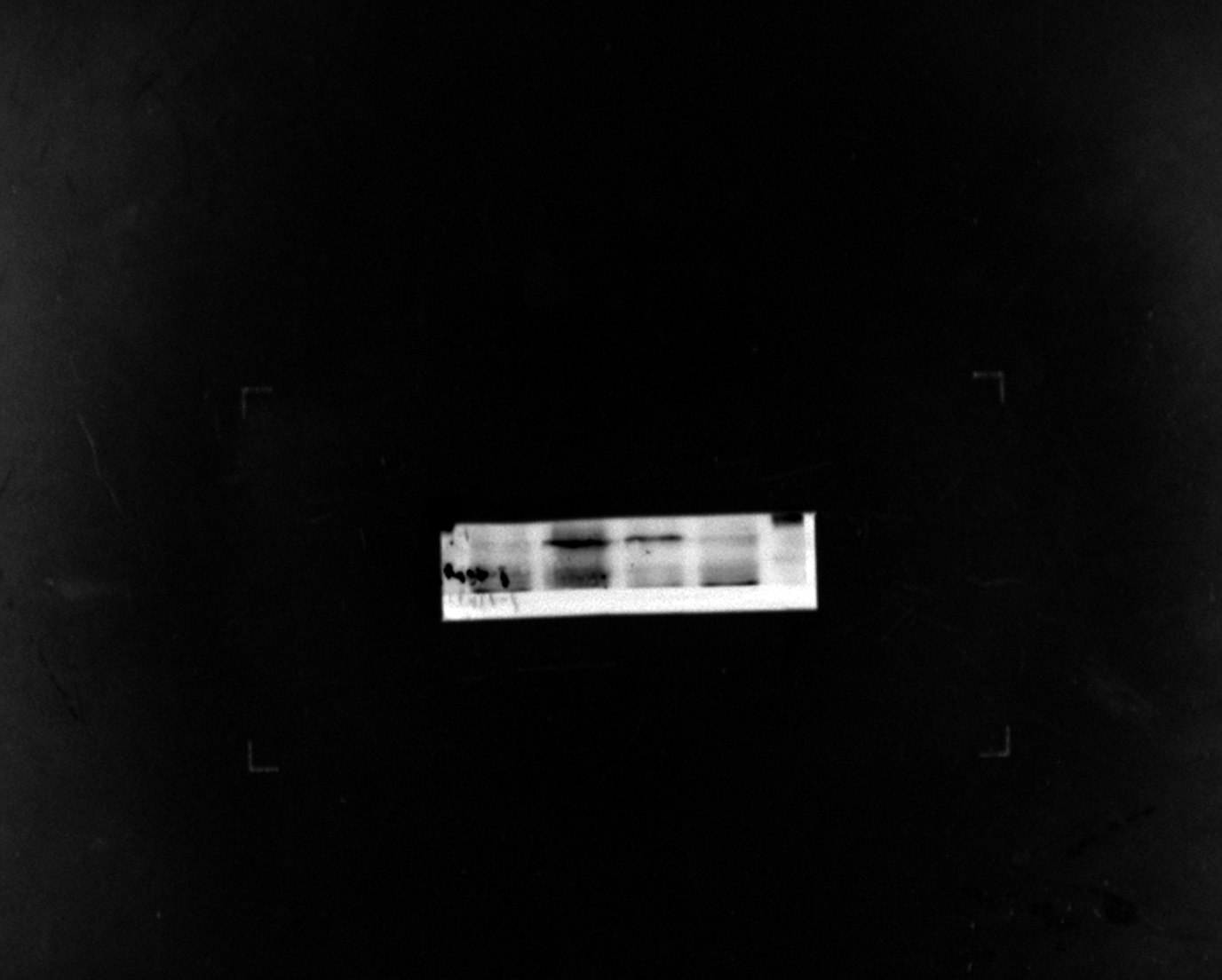

Supplement: Supplementary file 3 [file DataSheet4.ZIP › source data-2/WB-original gels/ConA models/P-AKT1-0.Tif]

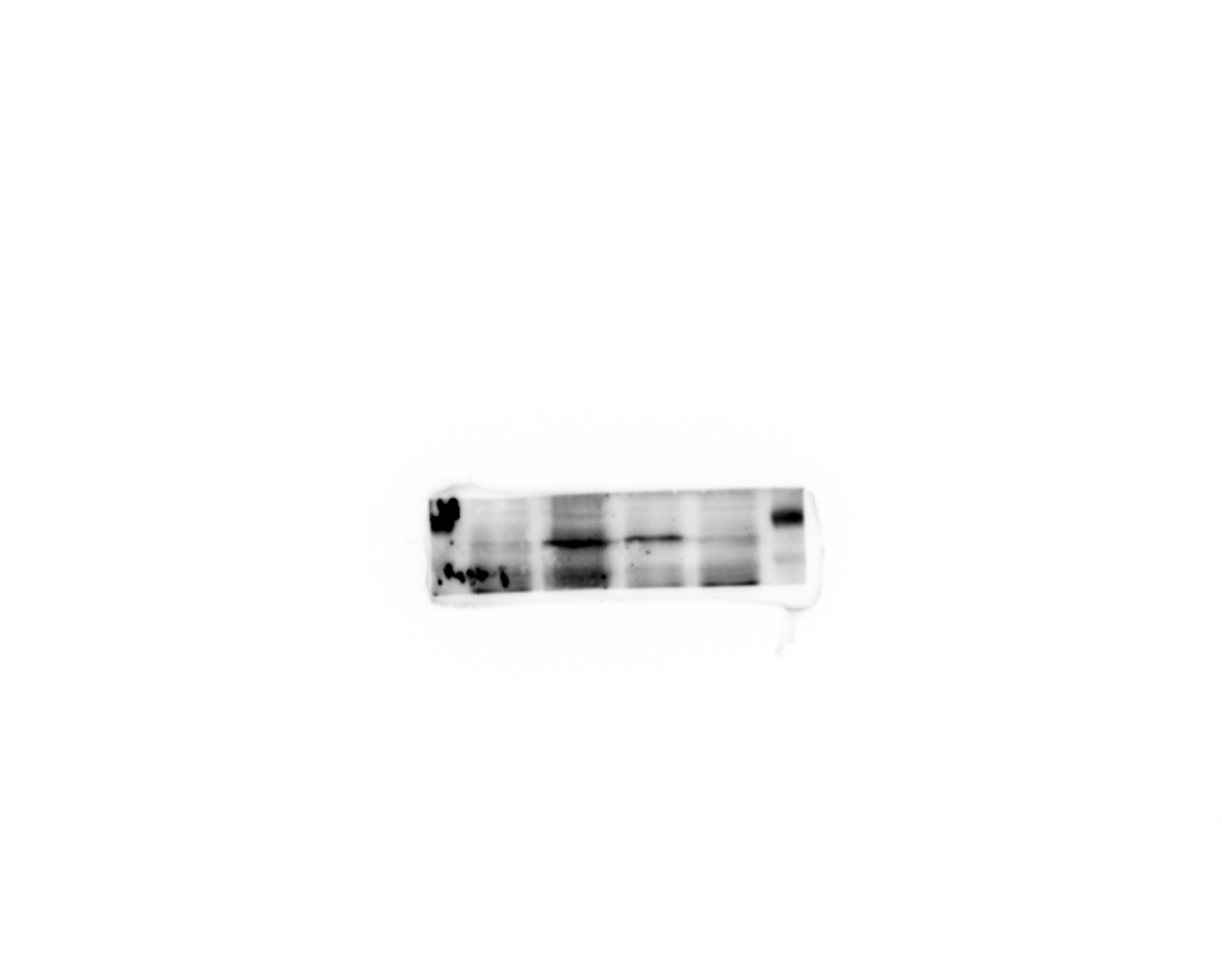

Supplement: Supplementary file 3 [file DataSheet4.ZIP › source data-2/WB-original gels/ConA models/P-AKT1.Tif]

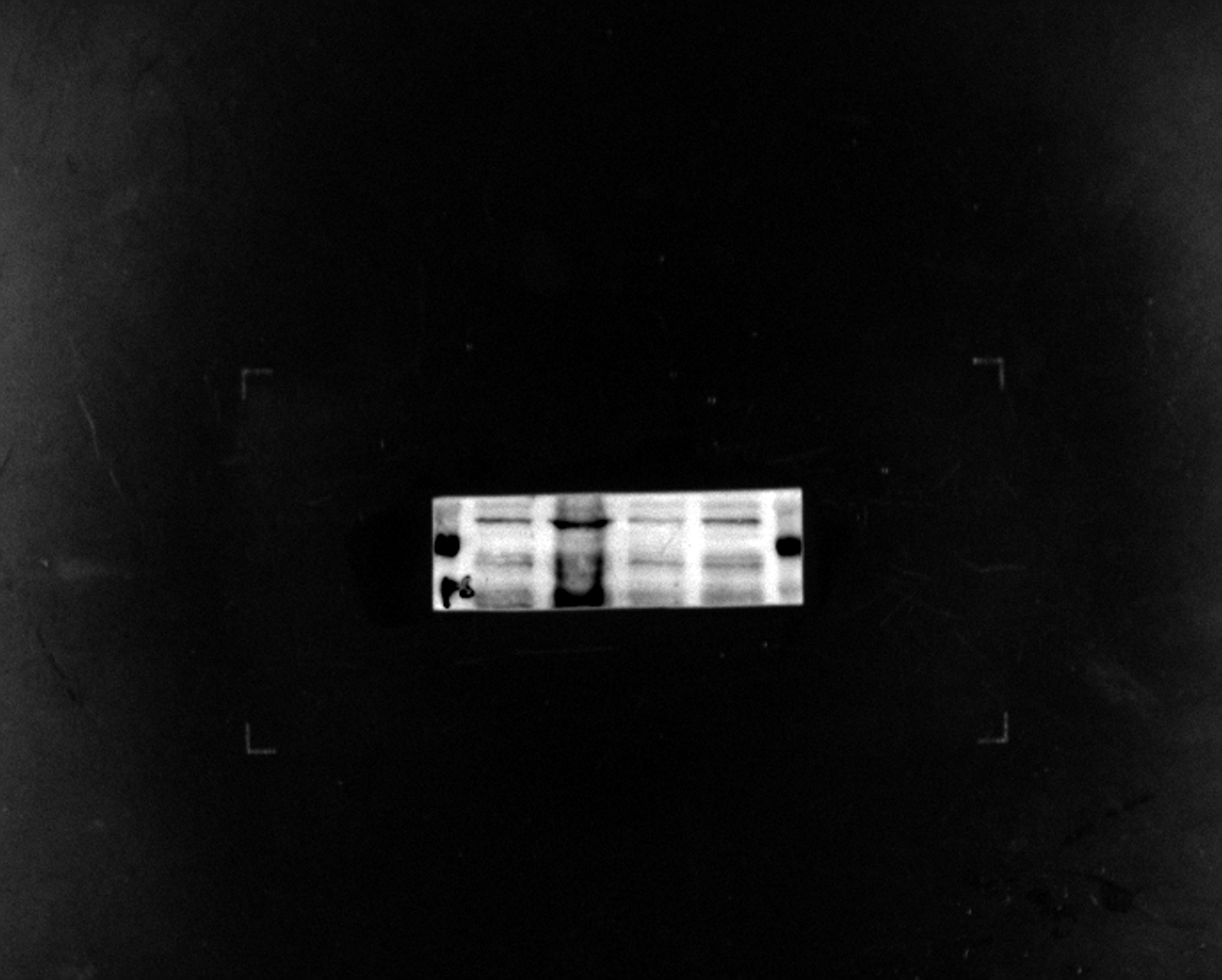

Supplement: Supplementary file 3 [file DataSheet4.ZIP › source data-2/WB-original gels/ConA models/P-PI3K-0.Tif]

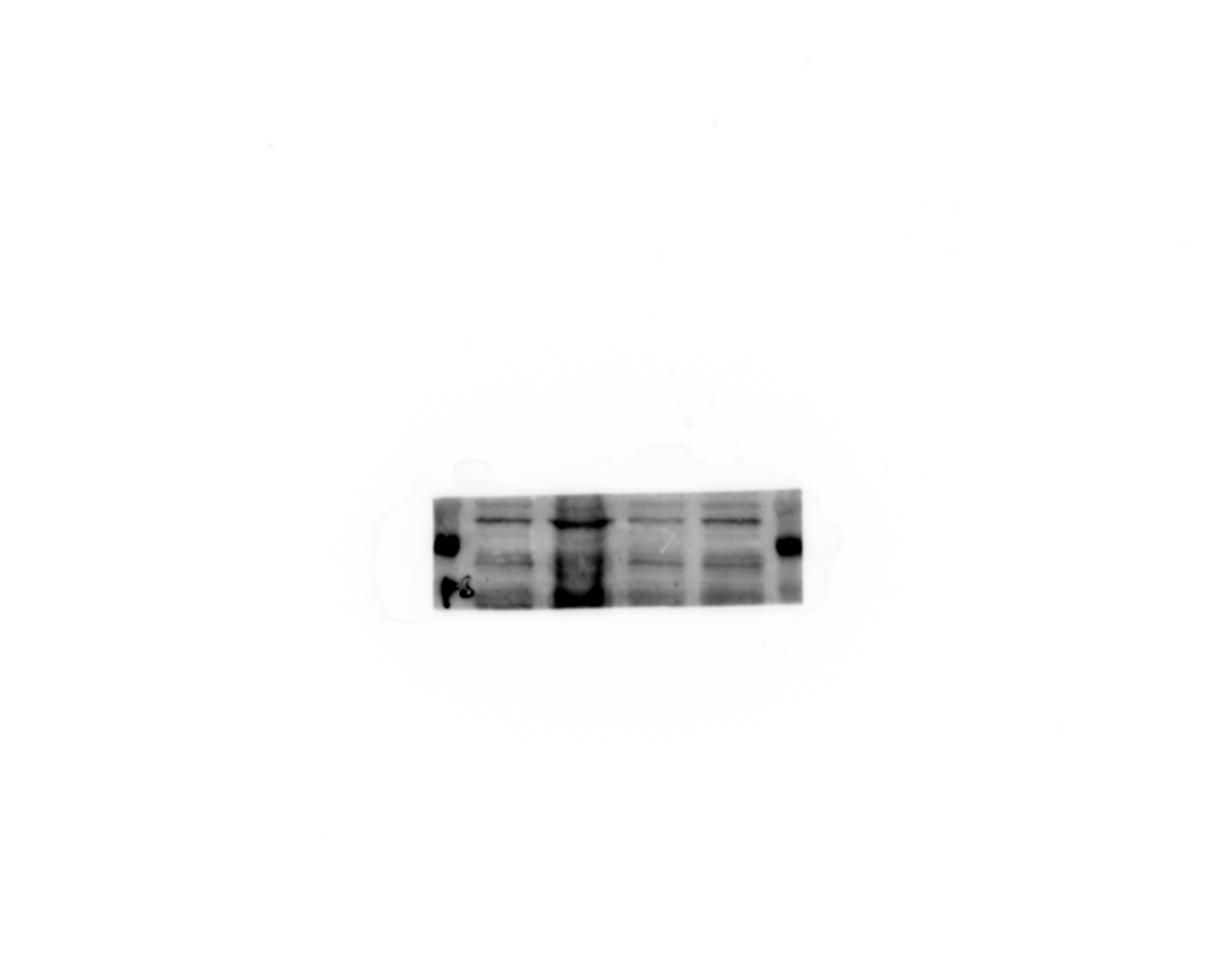

Supplement: Supplementary file 3 [file DataSheet4.ZIP › source data-2/WB-original gels/ConA models/P-PI3K.Tif]

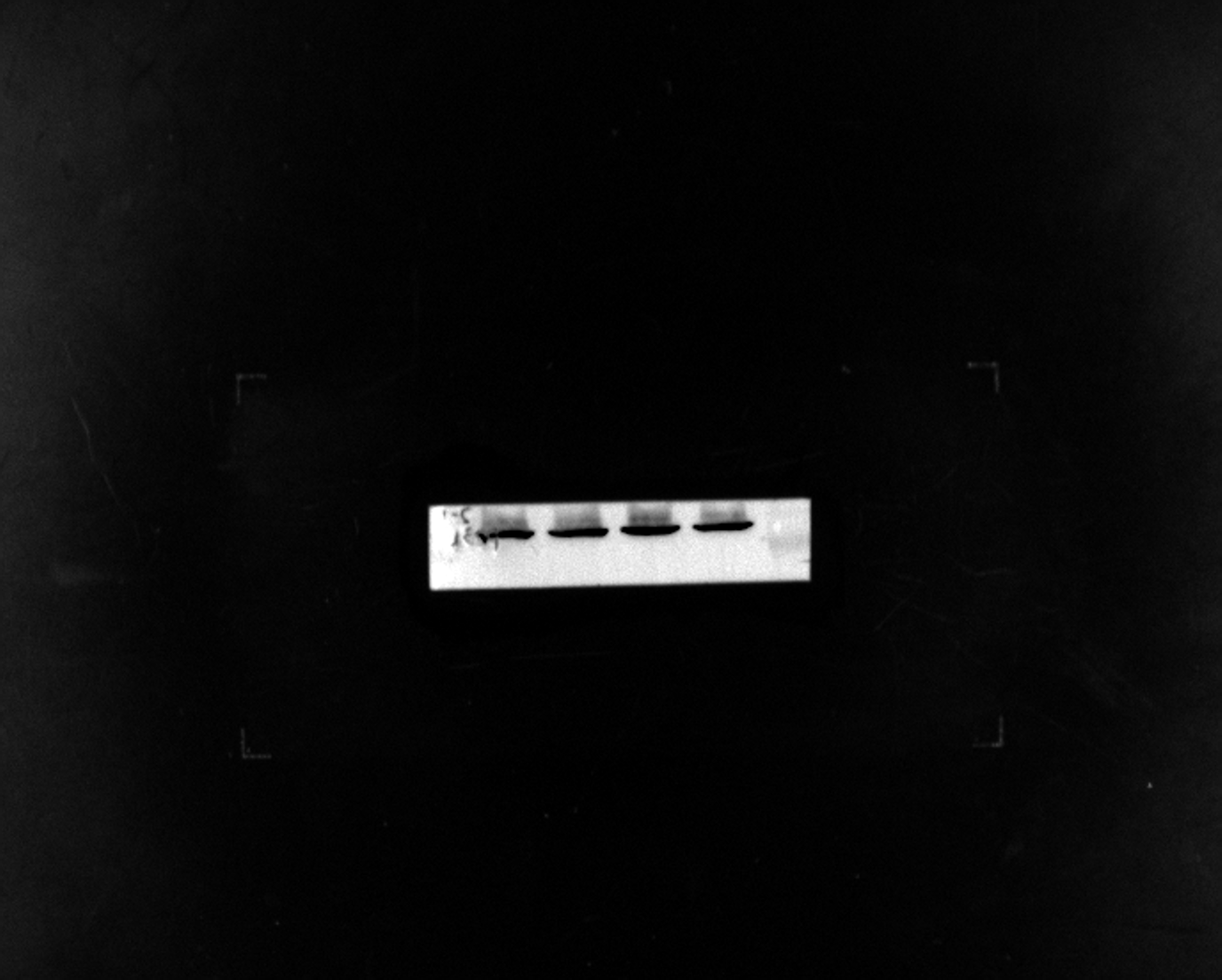

Supplement: Supplementary file 3 [file DataSheet4.ZIP › source data-2/WB-original gels/ConA models/PI3K-0.Tif]

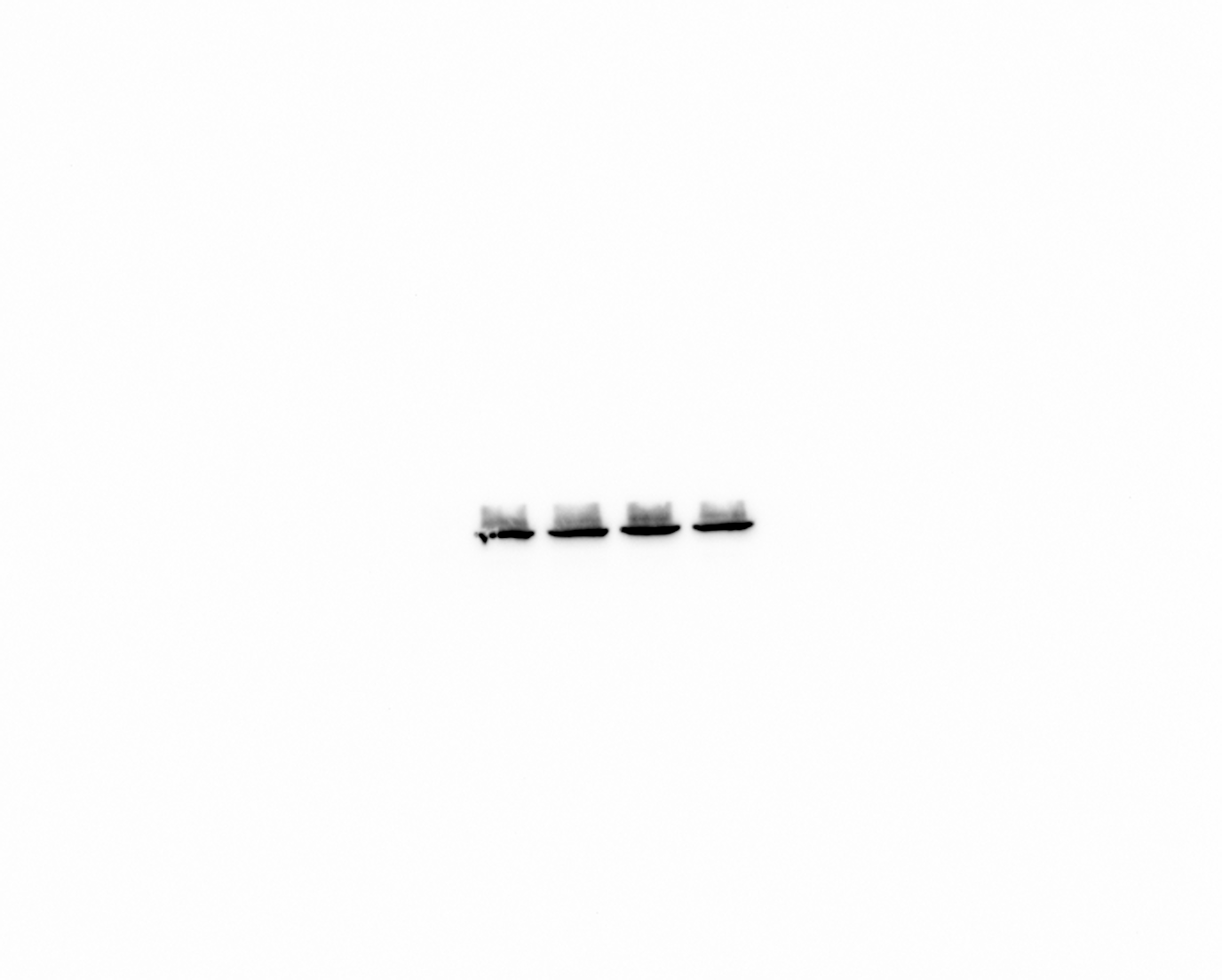

Supplement: Supplementary file 3 [file DataSheet4.ZIP › source data-2/WB-original gels/ConA models/PI3K.Tif]

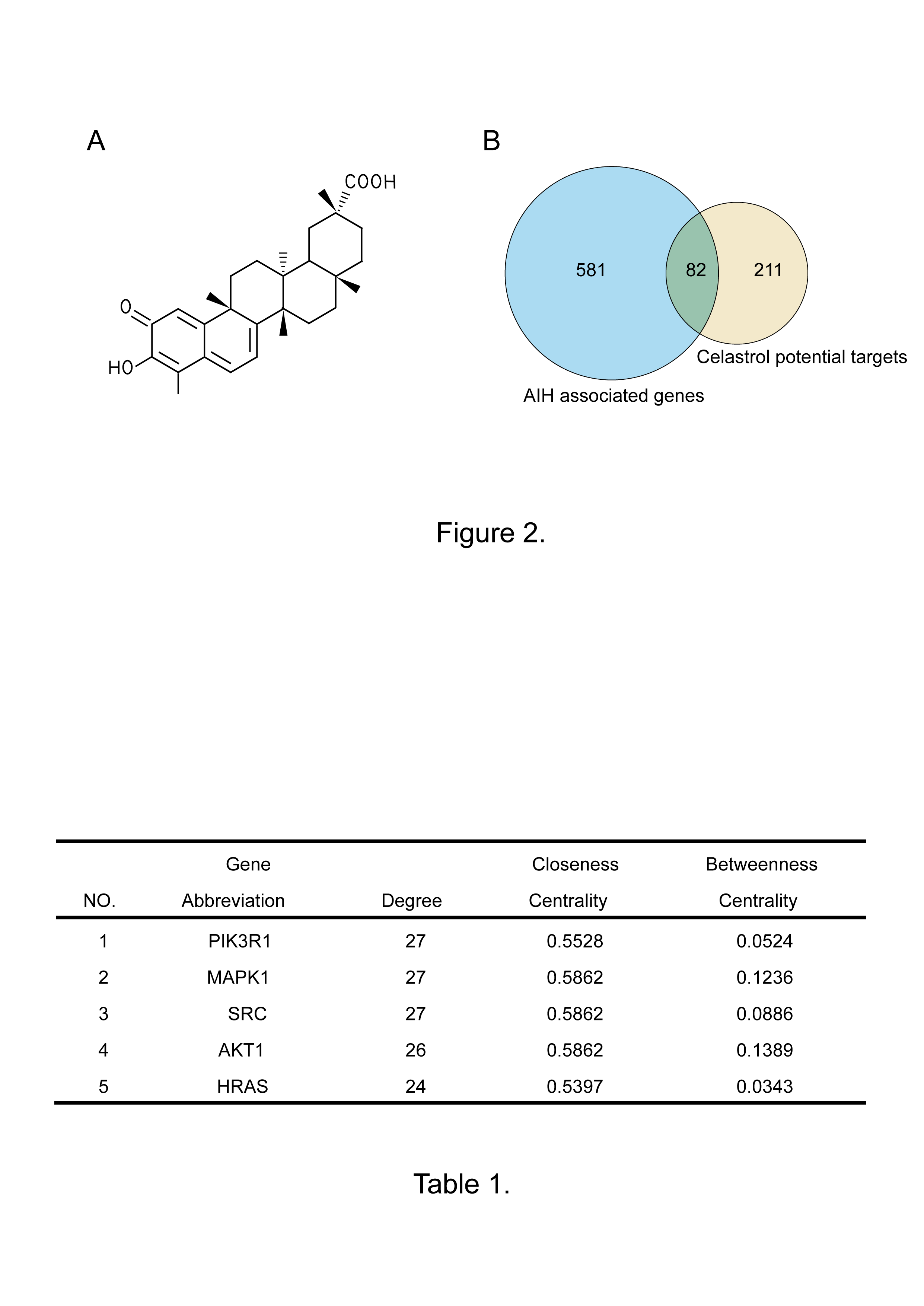

Supplement: Supplementary file 4 [file DataSheet1.ZIP › figure-1/Figures-02.tif]

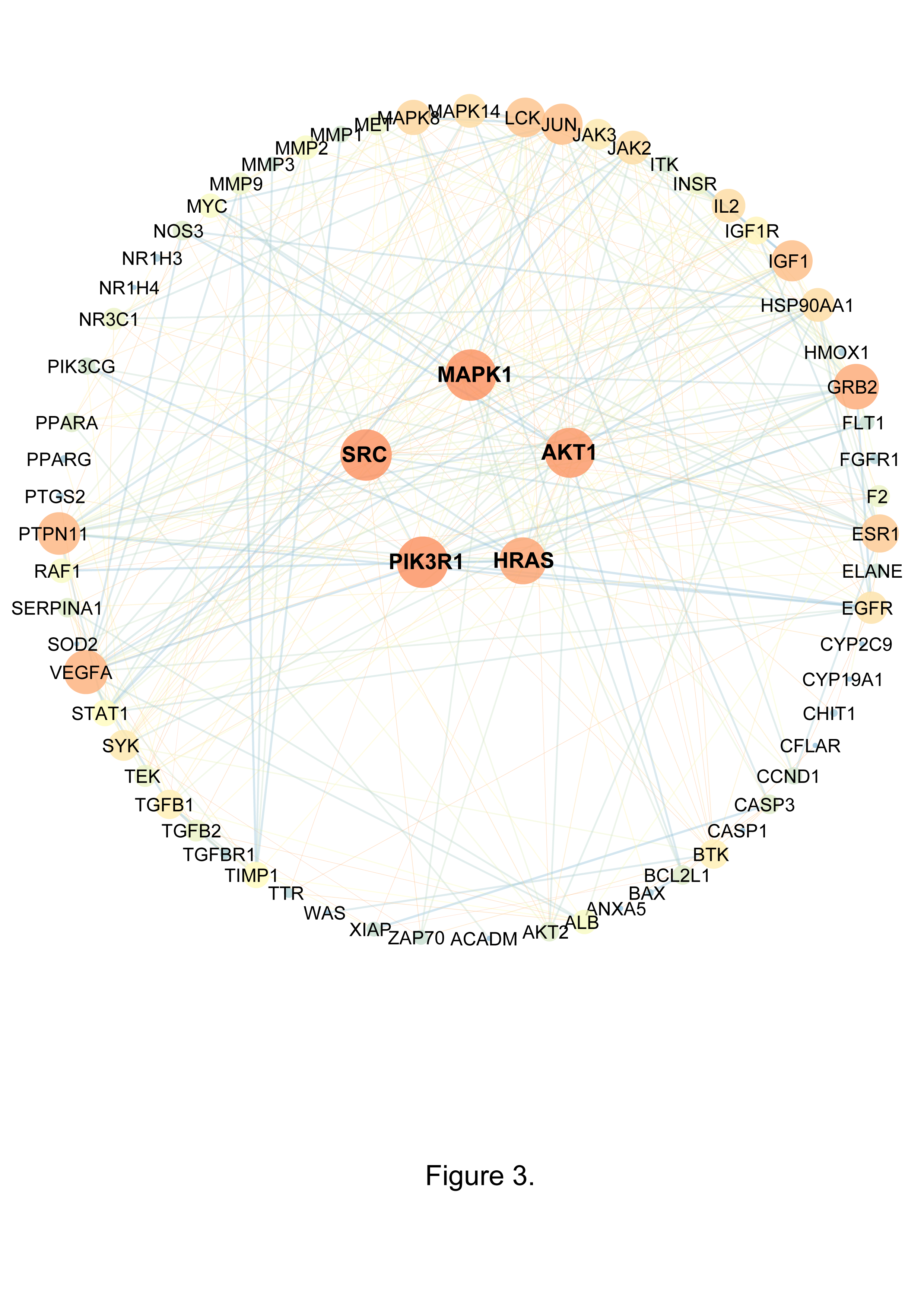

Supplement: Supplementary file 4 [file DataSheet1.ZIP › figure-1/Figures-03.tif]

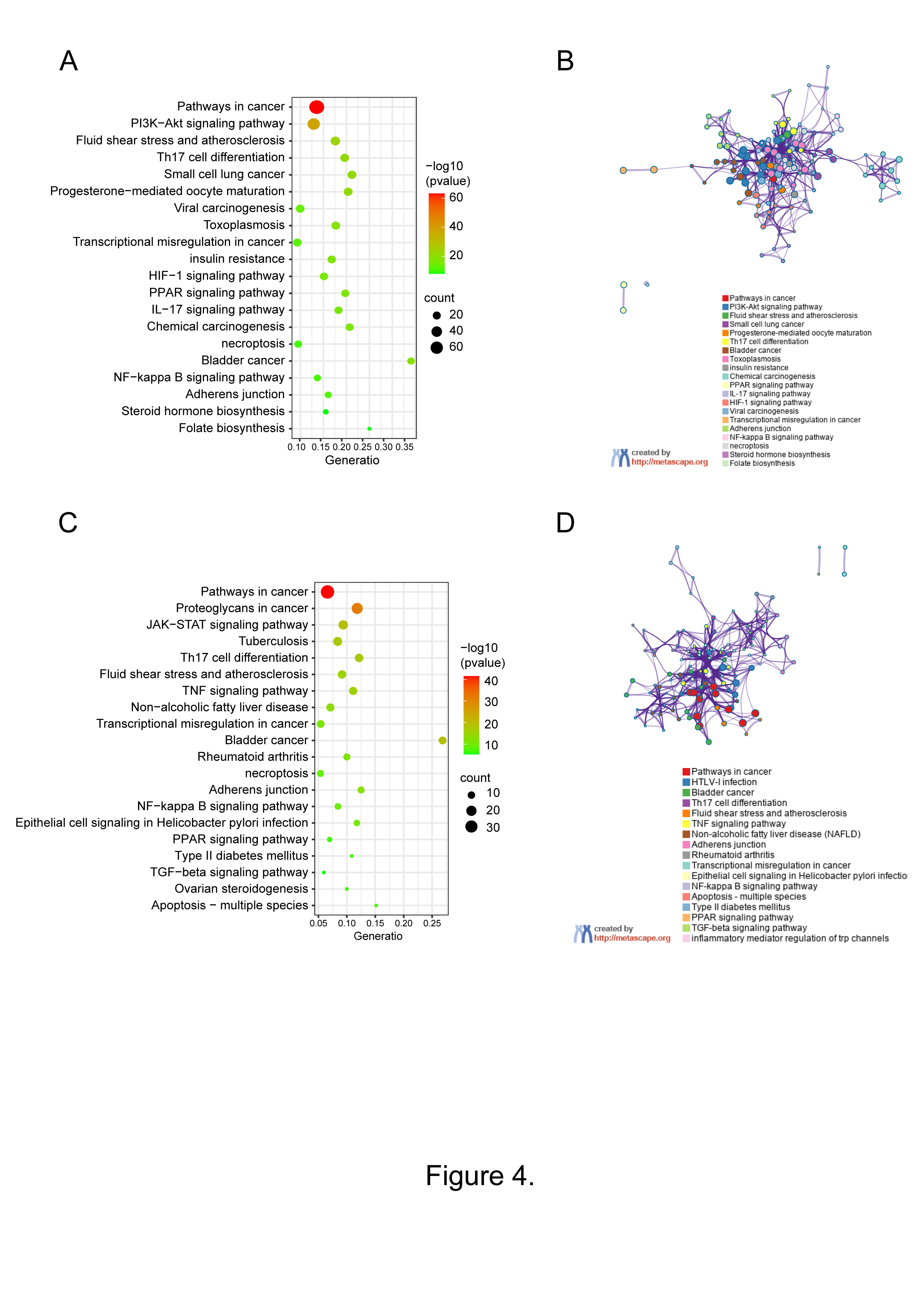

Supplement: Supplementary file 4 [file DataSheet1.ZIP › figure-1/Figures-04.tif]

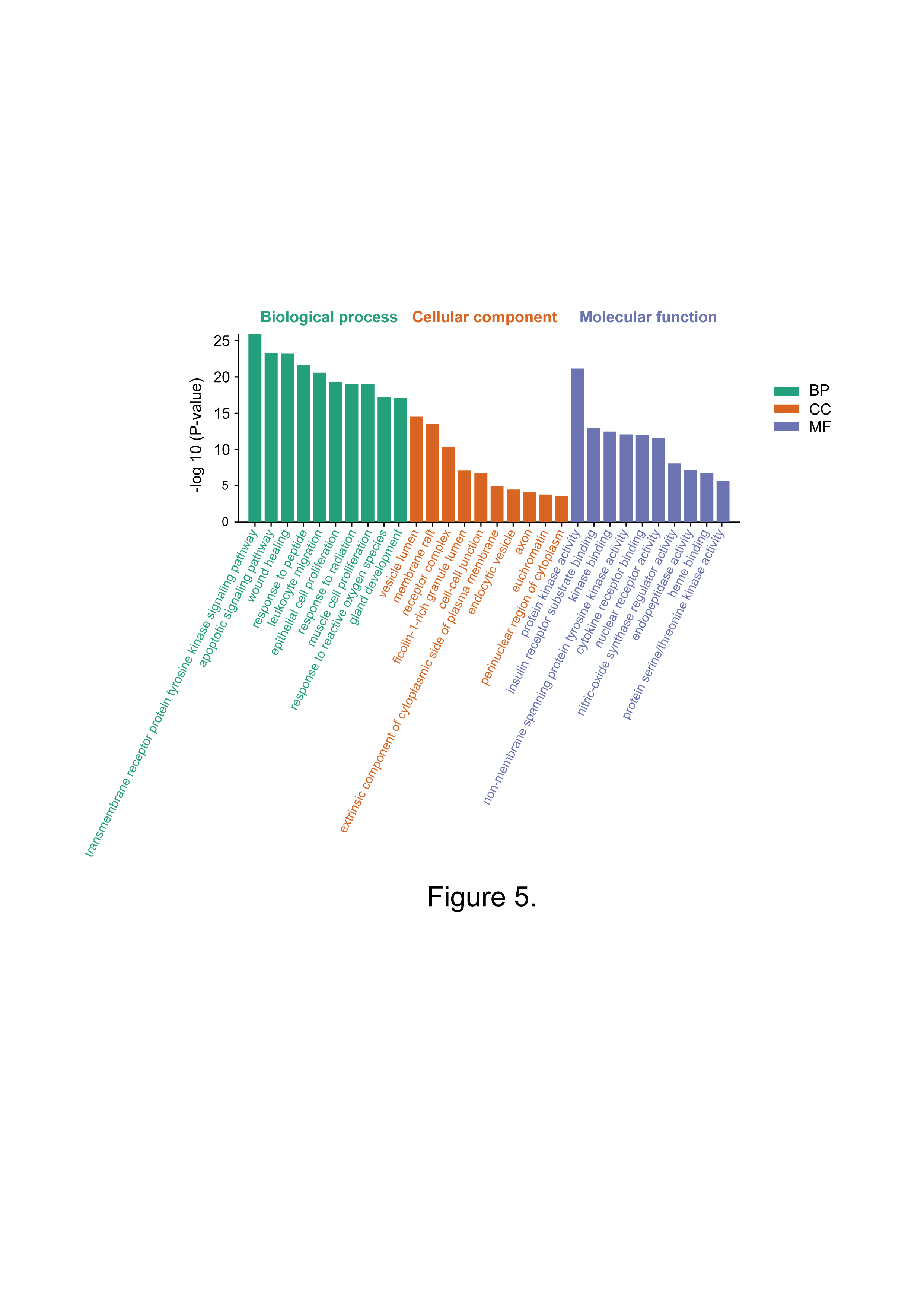

Supplement: Supplementary file 4 [file DataSheet1.ZIP › figure-1/Figures-05.tif]

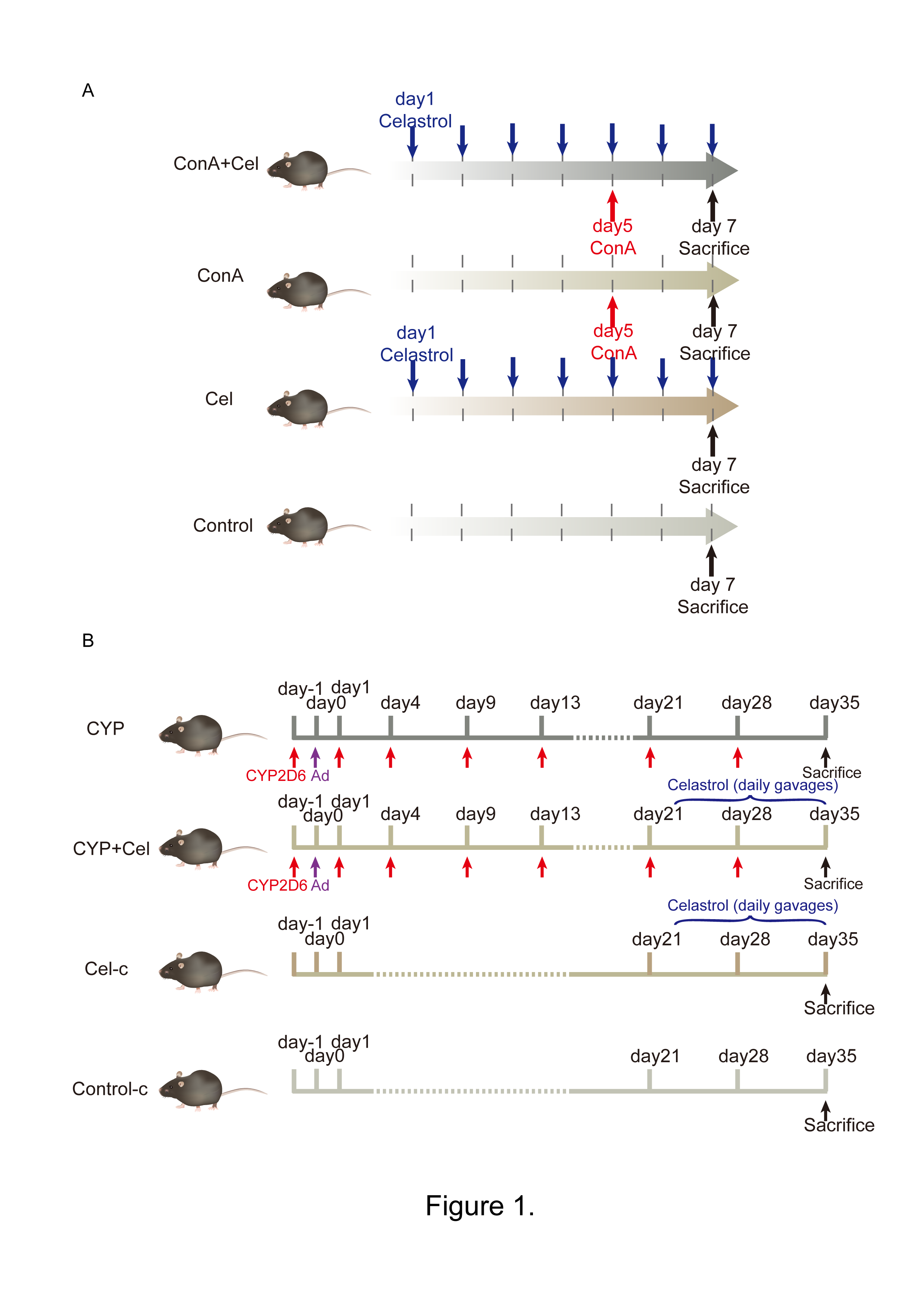

Supplement: Supplementary file 4 [file DataSheet1.ZIP › figure-1/Figures_画板 1.tif]

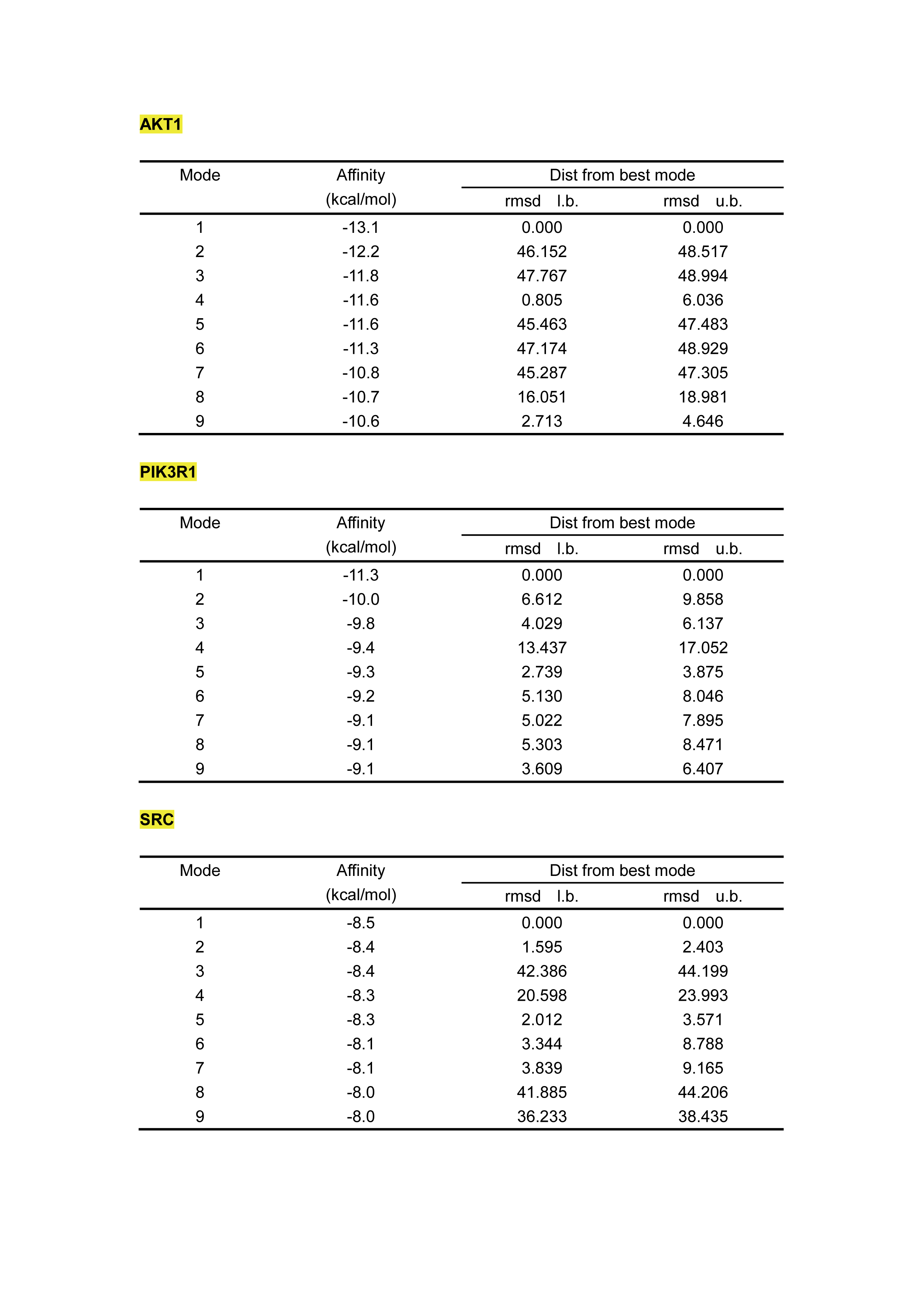

Supplement: Supplementary file 5 [file Image2.TIF]

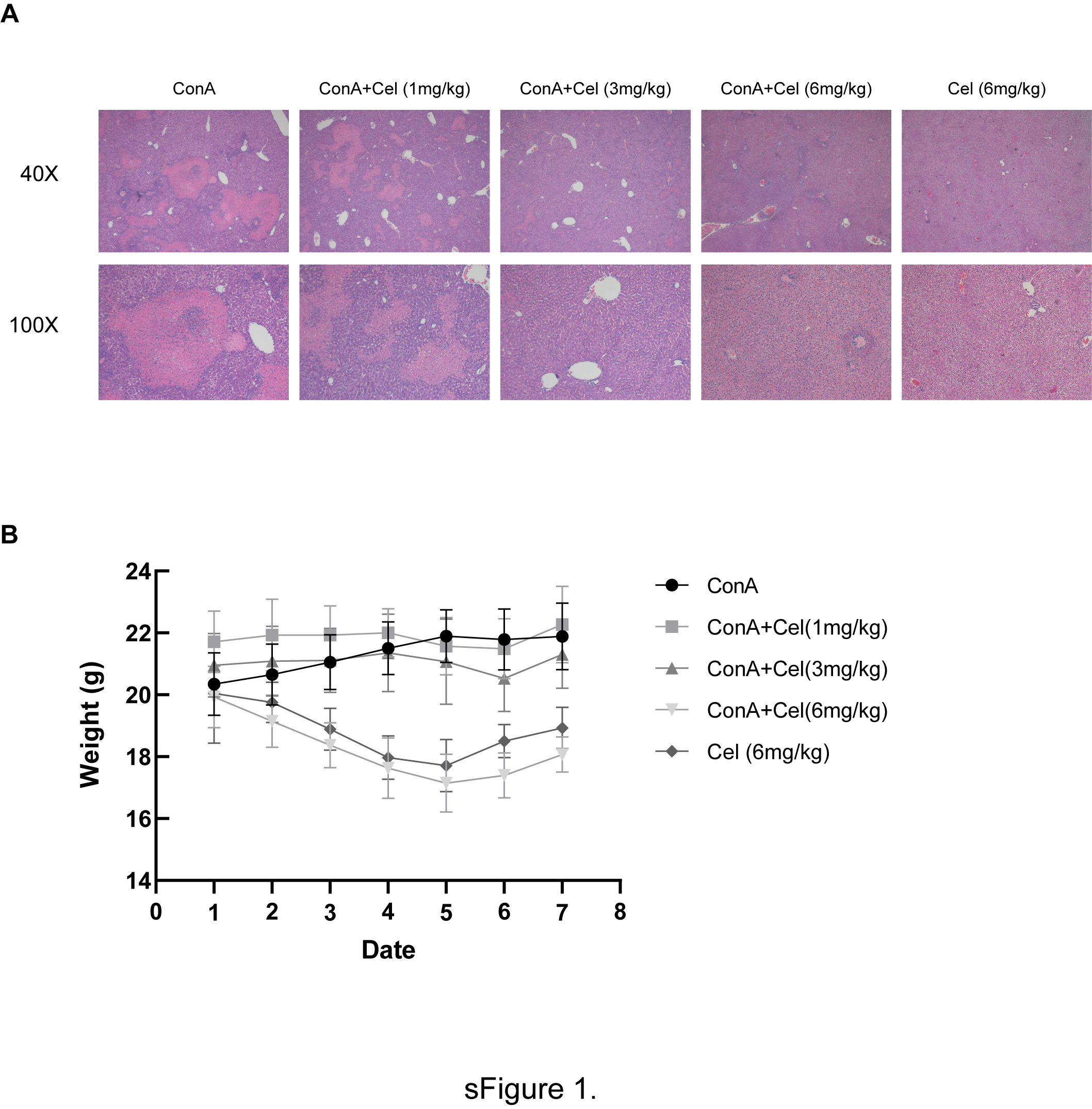

Supplement: Supplementary file 6 [file Image1.TIF]

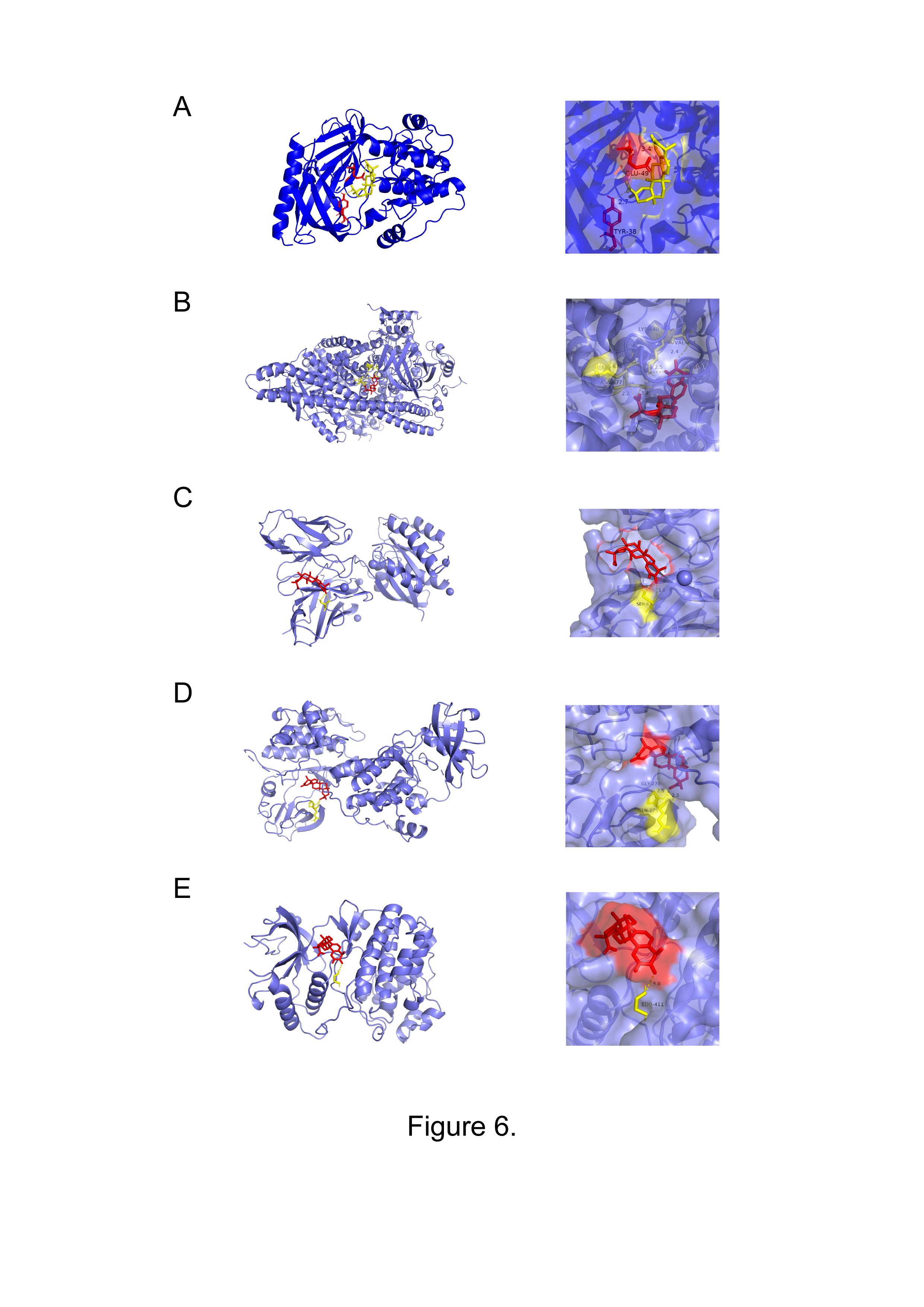

Supplement: Supplementary file 7 [file DataSheet2.ZIP › figure-2/Figures-06.tif]

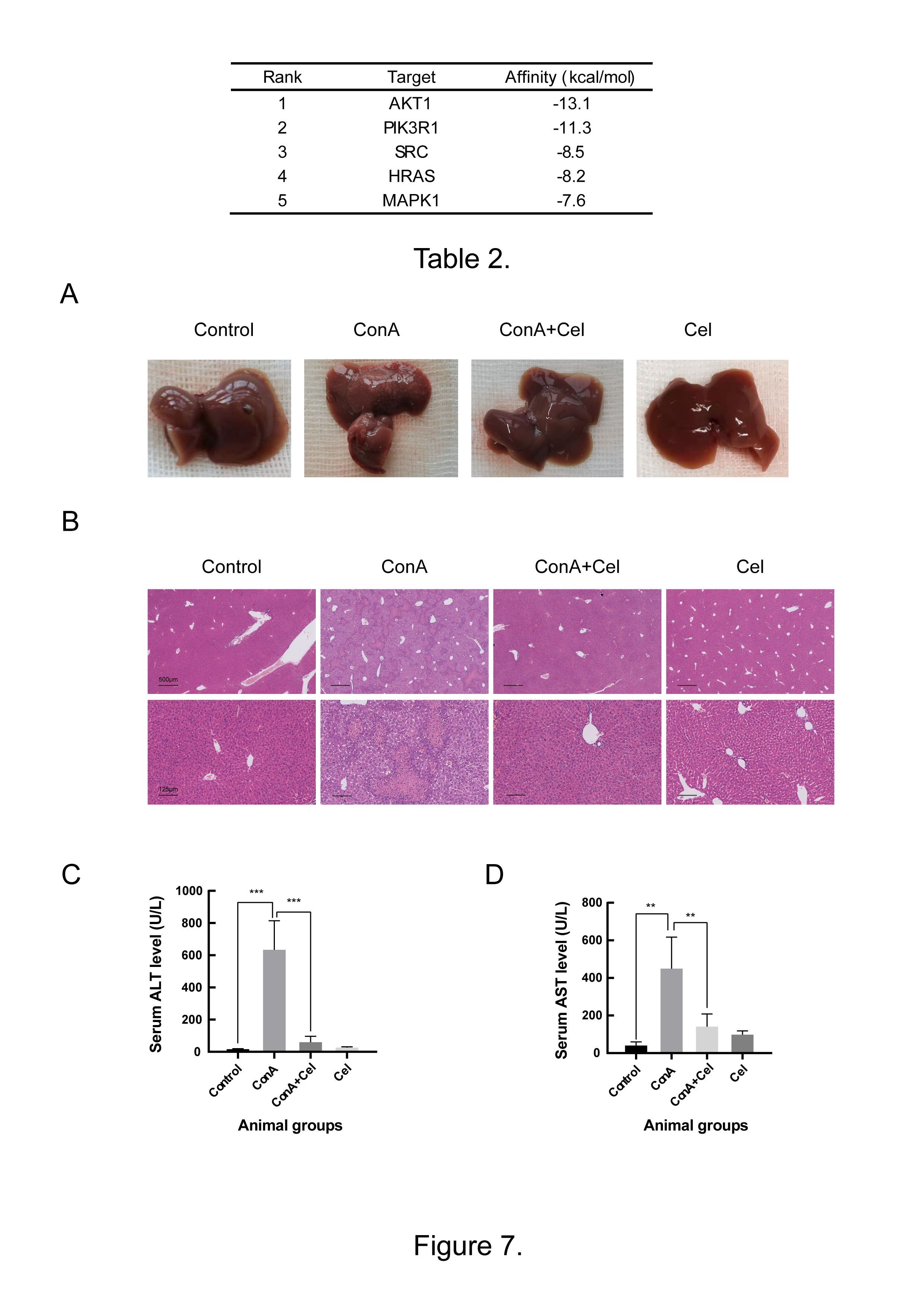

Supplement: Supplementary file 7 [file DataSheet2.ZIP › figure-2/Figures-07.tif]

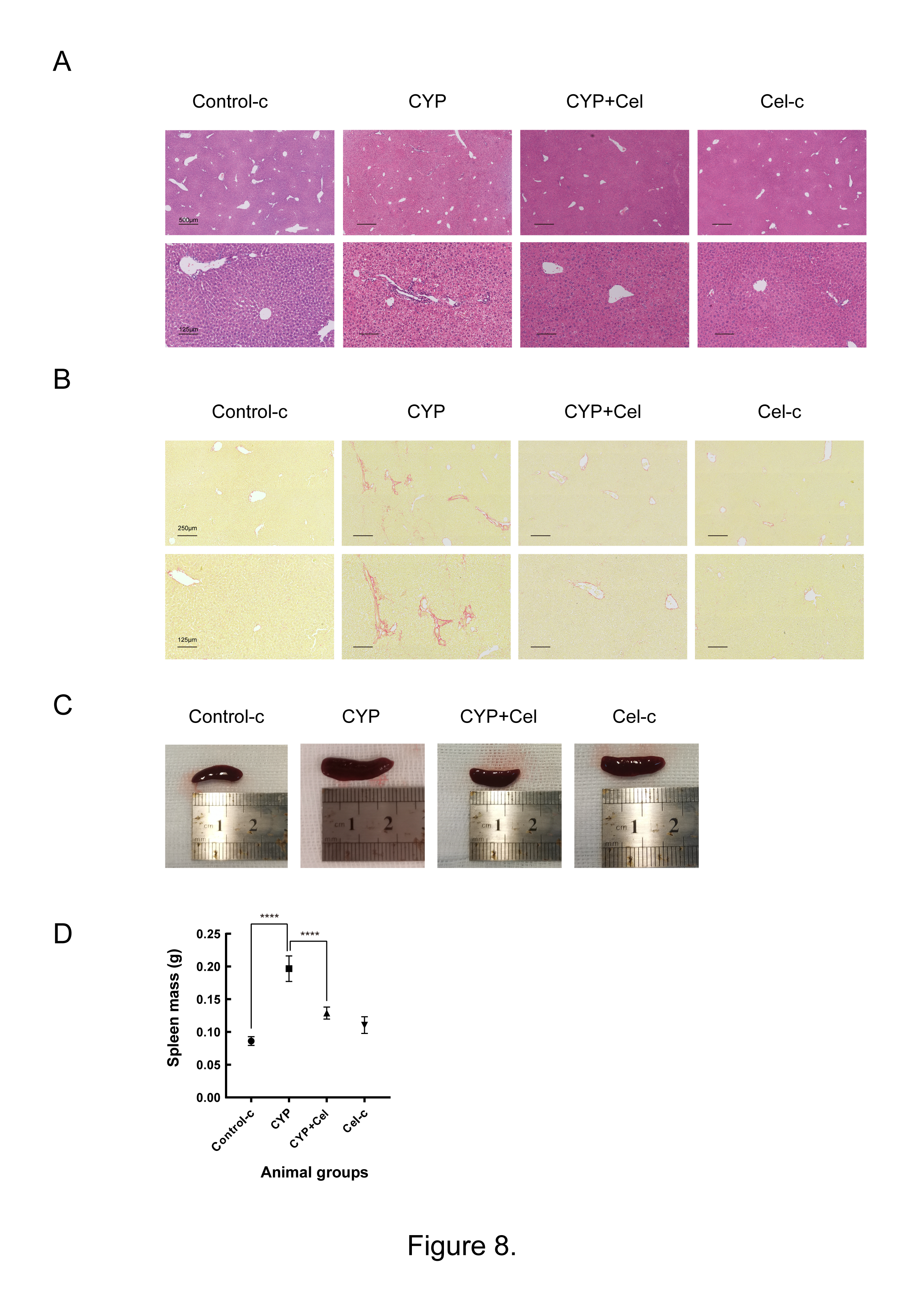

Supplement: Supplementary file 7 [file DataSheet2.ZIP › figure-2/Figures-08.tif]

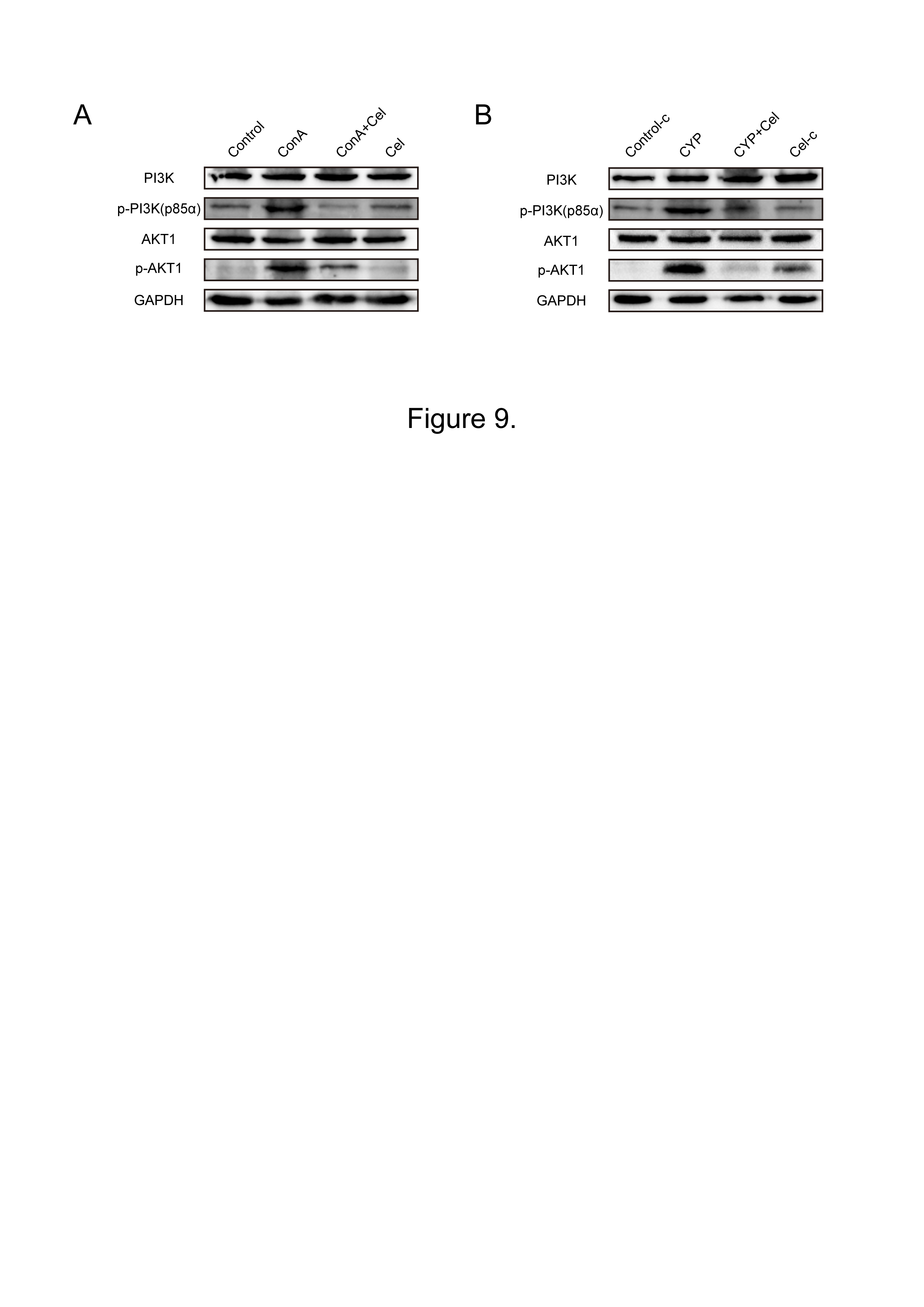

Supplement: Supplementary file 7 [file DataSheet2.ZIP › figure-2/Figures-09.tif]
